# Transcriptomics of long‐term, low oxygen storage coupled with ethylene signaling interference suggests neofunctionalization of hypoxia response pathways in apple ( *Malus domestica* )

**DOI:** 10.1002/pld3.70025

**Published:** 2024-12-20

**Authors:** John A. Hadish, Heidi L. Hargarten, Huiting Zhang, James P. Mattheis, Stephen P. Ficklin, Loren A. Honaas

**Affiliations:** ^1^ Molecular Plant Science Program Washington State University Pullman WA USA; ^2^ Department of Horticulture Washington State University Pullman WA USA; ^3^ USDA Agricultural Research Service Physiology and Pathology of Tree Fruits Research Wenatchee WA USA

**Keywords:** hypoxia, *M. domestica*, phylogenomics, postharvest, transcription factors

## Abstract

Research on how plants respond to hypoxia has concentrated on model organisms where tissues can only survive hypoxic conditions for a few hours to a few days. In contrast, hypoxic conditions are used commercially as a method to prolong the shelf life of *Malus domestica* (apple) fruit for up to a year of storage without substantial changes in fruit quality, not to mention a lack of tissue death. This ability of apples to withstand protracted hypoxic conditions is an interesting adaptation that has had limited molecular investigation despite its economic importance. Here, we investigate the long‐term apple hypoxia response using a time‐course RNA‐seq analysis of several postharvest storage conditions. We use phylogenetics, differential expression, and regulatory networks to identify genes that regulate and are regulated by the hypoxia response. We identify potential neofunctionalization of core‐hypoxia response genes in apples, including novel regulation of group VII ethylene response factor (ERF VII) and plant cysteine oxidase (PCO) family members.

## 1 INTRODUCTION

Groundbreaking work on the identification and revelation of the molecular mechanisms of group VII ethylene response factor (ERF VII) transcription factors (TFs) laid the foundation for our current understanding of plant responses to (and their recovery from) low oxygen environments (Gibbs et al., 2011; Hattori et al., 2009; Hinz et al., 2010; Licausi et al., 2010, 2011; Mustroph et al., 2010). Shortly after, plant cysteine oxidases (PCOs) were identified as critical enzymes which (in Arabidopsis) act in combination with constitutively expressed ERF VII proteins to regulate a “tunable oxygen‐sensing system” (Gasch et al., 2016; Weits et al., 2014). The activity of ERF VII genes is regulated at the protein level, where they are degraded via the n‐degron pathway in the presence of oxygen. PCOs require oxygen to oxidize the N‐terminal cysteine residue present on all ERF VII‐type proteins after the removal of Met by MAP (Giuntoli & Perata, 2018; Weits et al., 2014). This cysteine oxidation event flags ERF VII genes for degradation. However, in the absence of oxygen, PCO is unable to oxidize cysteine, which allows ERF VII TFs to bind to hypoxia‐responsive promoter element (HRPE) motifs of hypoxia‐responsive genes (HRGs) (Gasch et al., 2016), thereby activating the hypoxia response.

Model organisms such as Arabidopsis and *Oryza sativa* (rice) have been used to study transient responses to hypoxia during hypoxic stress response (flooding) and recovery over a matter of hours or days, generally in root tissue (Fu & Xu, 2023; León et al., 2021; Papdi et al., 2015). Nonmodel organisms, such as *Malus domestica* (apple), present excellent new opportunities to explore how core hypoxia response mechanisms have been adapted across land plants and to provide insights to the response that operate on much longer time scales. Especially relevant is that the response to low oxygen environments is rooted in ethylene signaling and perception; this presents the possibility for signaling pathway crosstalk in climacteric fruits such as apple that use ethylene signaling to regulate fruit maturity and ripening (Cukrov, 2018). Indeed, recent research has observed crosstalk among hypoxia, ethylene signaling, and fruit maturation pathways. In tomatoes, ERF VII orthologs were observed to be involved in regulating fruit ripening (Liu et al., 2016), including acting as negative regulators of carotenoid accumulation (Lee et al., 2012), indicating an important relationship between hypoxia signaling, ethylene response factors, and fruit ripening (Cukrov, 2018). In persimmon, hypoxia‐responsive ERFs were demonstrated to have dual roles in both regulation of low oxygen metabolism genes and deastringency associated with ripening (Min et al., 2012, 2014; Wang et al., 2017; Zhu et al., 2018). Furthermore, pre‐climacteric fruit (immature fruits) have been observed to be more tolerant to low oxygen stress compared to postclimacteric fruits (mature fruits) (Ke et al., 1994), and fruit of more advanced maturity are more likely to ferment in low oxygen conditions (Both et al., 2016).

In commercial packing houses, hypoxia (i.e. controlled atmosphere or CA) is used as a tool to inhibit fruit ripening and prolong storage in the postharvest period. Fruit is kept in low or ultralow oxygen conditions (often below 1% oxygen) for months, and sometimes as long as a year (Brizzolara et al., 2020). In apples (and other large storage organs), this prolonged exposure to low oxygen is not necessarily unnatural, as such storage organs have been shown to have constitutive hypoxic conditions internally (aka hypoxic niches; Geigenberger et al., 2000; Rolletschek et al., 2002; Ho et al., 2008, 2011; Licausi et al., 2011; Cukrov, 2018; Loreti & Perata, 2020). The nature of the success of low oxygen storage, coupled with the natural physiology of fruits, provides an opportunity to explore how pome fruits have evolved the ability to withstand such prolonged hypoxia.

In addition to low‐oxygen (CA) storage of apples, there are two other commonly used strategies for postharvest management of conventional apple fruit in storage: refrigeration and the application of the ethylene perception inhibitor 1‐Methylcyclopropene (1‐MCP). Together, these strategies are used to slow metabolic activity (chilling and CA) and interfere with the molecular mechanisms of the ripening process by inhibiting ethylene sensing and signaling (CA and MCP; Cukrov, 2018). By blocking ethylene perception, 1‐MCP generally inhibits respiration rates, reduces the rate of softening, prevents loss of greenness, inhibits greasiness, and has a mixed effect on titratable acidity, volatile content, soluble solid concentration, and physiological postharvest disorders (Watkins, 2006 and references therein; Lv et al., 2020). 1‐MCP treatment has also been demonstrated to reduce the accumulation of reactive oxygen species in fruit exposed to chilling stress (Sabban‐Amin et al., 2011). As an inhibitor of ethylene perception, 1‐MCP application may affect apple fruit's adaptive responses to low oxygen environments, either through enhancement (Mattheis et al., 2005; Rupasinghe et al., 2000; Watkins, 2006; Watkins et al., 2000) or impairment, as evidenced by certain cultivars (such as “Honeycrisp”) becoming more susceptible to low oxygen injury when treated with 1‐MCP (Chiu et al., 2015).

Exploration of the impacts of 1‐MCP in concert with CA on apple fruit has received interest with regard to fruit quality outcomes (DeEll et al., 2016; DeEll & Lum, 2017; Poirier et al., 2020; Watkins et al., 2015; Zanella, 2003), metabolomics (Bekele et al., 2014; Hatoum et al., 2016), and increasingly through the lens of transcriptomics (Cukrov et al., 2016; Johnson & Zhu, 2015; Li et al., 2022; Vittani et al., 2023). Understanding how ethylene perception inhibition affects transcriptomic responses to low oxygen will aid in our understanding how fruit quality is influenced by these long‐term storage methods and improve management tools for fruit quality. It is also likely that there are novel adaptations in gene function in apple fruit which allow them to survive for up to a year in hypoxic conditions (Brizzolara et al., 2017). In this paper, we use transcriptomics to (1) identify core genes associated with responses to long‐term hypoxic conditions in apple fruit; (2) gain insight into the role ethylene plays in these long‐term responses; (3) suggest apple homolog‐specific adaptations to the current hypoxia molecular model (Giuntoli & Perata, 2018). We identified potential neo‐functionalization of putative core‐hypoxia response genes in apples, including novel transcriptomic regulation of ERF VII and PCO family members. We also showed that apple fruit transcriptomic response to long‐term hypoxic storage can be loosely divided into two responses, the first is an early response very likely controlled by the n‐degron pathway, and the second response takes months in low‐oxygen storage to evolve and is likely controlled by ethylene sensing and signaling.

## 2 RESULTS

### 2.1 Over 600 putative hypoxia response genes identified in apple

**FIGURE 1:**
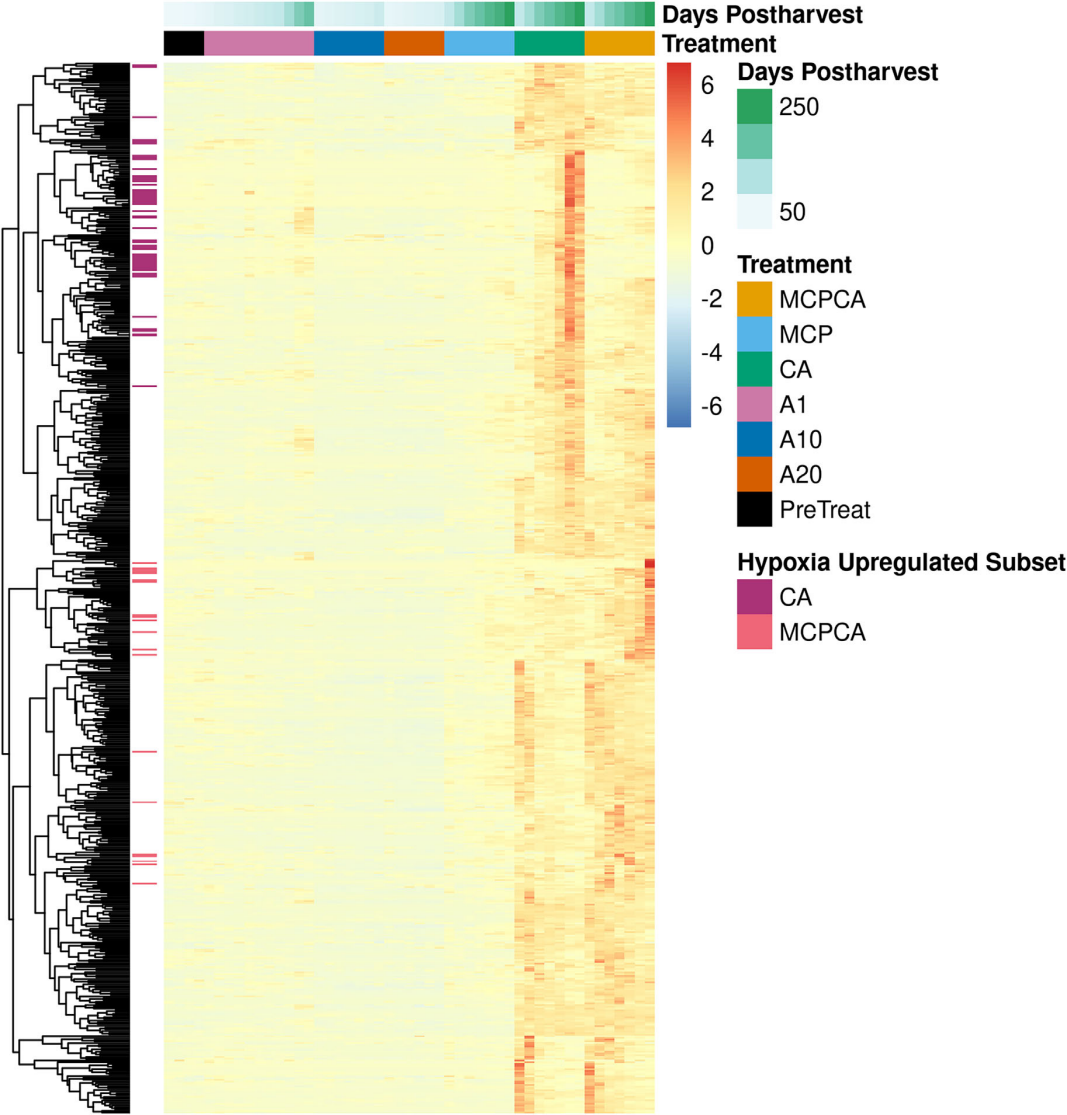
A total of 606 genes in the apple GDDH13 genome were differentially upregulated in hypoxic conditions. Genes (rows) were clustered based on expression pattern similarity. The *y*‐axis annotation between the dendrogram and heatmap, titled “Hypoxia Upregulated Subset,” highlights genes that are differentially upregulated under one of the hypoxia treatments (MCPCA (Controlled Atmosphere + 1‐MCP Treatment) and CA (Controlled Atmosphere)) and not the other. The *x*‐axis annotations above the heatmap indicate samples grouped by treatment and ordered by days postharvest (the earliest time point sample is first within each treatment). Each cell represents the average expression of three biological replicates.

A total of 606 genes were identified as differentially upregulated during the hypoxia response when performing pairwise comparisons of all normoxic treatment groups (PreTreat (Pre Treatment), A1 (Air, 1°C), A10 (Air, 10°C), A20 (Air, 20°C), MCP (Air, 1°C + 1‐MCP treatment) to each of the hypoxic treatment groups (CA (Controlled Atmosphere, 1°C) and MCPCA (Controlled Atmosphere + 1‐MCP Treatment, 1°C)) (Figure 1; Table S1). Within the group of 606 putative hypoxia response genes, 52 were identified as differentially upregulated only in the CA treatment (hypoxia upregulated CA only subset), and 20 were only upregulated in the MCPCA treatment (hypoxia upregulated MCPCA only subset) (Figure S1; Table S2). Arabidopsis homologs and gene annotations for the 606 putative hypoxia response genes are available in Tables S1.

Functional enrichment of the 606 putative hypoxia genes revealed highly significant functional groups (*p* < .00005) related to oxygen sensing and hypoxia (GO:0036293, GO:0070482, GO:0001666, GO:1901700) and energy management (GO:0015979, GO:0019684, GO:0009765, GO:0009055, and GO:0004022). A complete list of enriched GO terms is available in Table S3, and a hierarchical dendrogram of these terms is available as Figure S2. GO term enrichment analysis for the hypoxia upregulated CA and MCPCA subsets did not produce any significantly enriched terms, likely due to the small number of genes in each subset (52 and 20 respectively). However, the Arabidopsis homologs of apple genes within these two subsets are known to be upregulated in response to both ethylene and hypoxia, and include ACC Oxidase (ACO1; MD15G1205100/AT2G19590.1‐CA only subset), Ethylene responsive element binding factors, including ERF1 and ERF2 (CA only subset; Hartman et al., 2019), and Hypoxia Response Unknown Protein 26 (HUP26) (MCPCA only subset; Huh, 2021), and members of the Cytochrome P450 superfamily (both CA only and MCPCA only) (Blokhina & Fagerstedt, 2010; Safavi‐Rizi et al., 2020).

In Mustroph et al. (2009), 49 genes were reported in Arabidopsis as core‐induced genes during hypoxia in root tissue. These 49 Arabidopsis genes are orthologous to 87 genes from the GDDH13 apple genome (Table S4). Twenty seven of the apple orthologs were identified in our list of 606 differentially upregulated hypoxia genes (Figure S3), and two of those genes were isolated to the CA only subset. That only about 25% of the apple orthologs to the “core 49” Arabidopsis hypoxia genes is unsurprising for several reasons. Apple is highly heterozygous and has experienced whole genome duplications, making confident assignments of orthologs between these two evolutionarily distinct organisms challenging. Indeed, for four of the core 49 Arabidopsis hypoxia response genes, we were unable to assign apple orthologs from the GDDH13 apple genome due to low bootstrap support, absence of orthologs in appropriate clades, or absence of GDDH13 genes in the gene family. Two of the “core 49” Arabidopsis hypoxia response genes were not assigned to orthogroups based on the PlantTribes 26Gv2 scaffold. Within the group of 87 GDDH13 orthologs, 10 groups of potential tandem duplicates or poor gene models were identified (see Supplemental Methods for more details), potentially causing an artificial inflation in the number of apple orthologs. Additionally, our study focused on upregulated hypoxia response genes, while the “core 49” Arabidopsis hypoxia response genes include both upregulated and downregulated genes. Furthermore, the Arabidopsis gene expression data set was developed from root tissue, while our gene expression data was developed from fruit cortex tissue.

### 2.2 Early and delayed responses to low oxygen storage are controlled by two suites of genes that differ in their dependency on ethylene sensing and signaling

**FIGURE 2:**
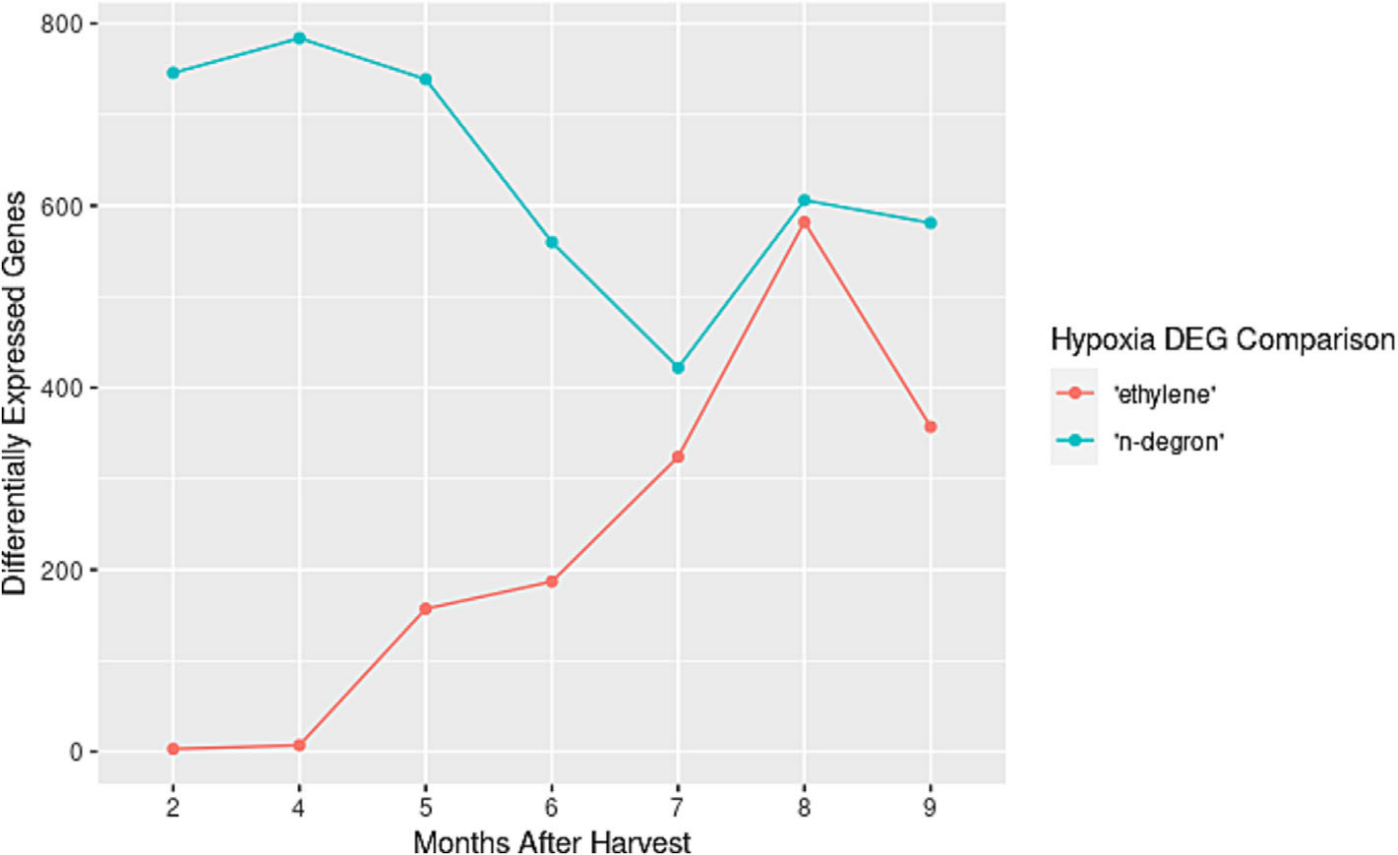
Different signaling pathways are engaged over the long‐term hypoxia response in “Gala” apple. Genes that were upregulated in CA and MCPCA when compared to MCP are those genes putatively involved in the n‐degron pathway (“n‐degron” teal line). Genes that were upregulated in CA when compared to MCPCA and MCP are those putatively mediated by ethylene sensing and signaling (“ethylene,” salmon line). Abbreviations: CA, controlled atmosphere; MCP, Air + 1‐MCP Treatment; MCPCA, controlled atmosphere + 1‐MCP Treatment.

A third pairwise differential expression analysis was performed between the long‐term fruit (MCP, CA, MCPCA) at each time point to assess trends in gene upregulation related to hypoxia response and the role of ethylene perception. Genes that were upregulated in hypoxic conditions both with and without the ethylene blocker 1‐MCP (CA and MCPCA) compared to the normoxic MCP treatment are defined as putative hypoxia “n‐degron”–related genes. This is because they are upregulated in response to hypoxia regardless of the fruit's ability to perceive ethylene. Because the n‐degron pathway is dependent on oxygen perception to detect hypoxia—and not ethylene perception—we refer to this group of genes as “n‐degron” related genes. This designation is putative based on our observational transcriptome experiments, and functional experiments would need to be done to verify that these genes are in fact regulated via the n‐degron pathway. Genes upregulated in CA compared to both MCPCA and MCP were considered “ethylene”‐related hypoxia genes because they are only upregulated in hypoxic conditions when ethylene is able to be perceived (CA condition only). This is in contrast to the “n‐degron”‐related genes which are upregulated in response to hypoxia with or without ethylene. The number of upregulated differentially expressed genes (DEGs) at each time point for these two groups shows opposite patterns (Figure 2). At least 400 DE “n‐degron” genes at each time point were observed during the 9‐month storage period and showed a decreasing trend over time (Figure 2; Table S5). In contrast, the number of DEGs classified as “ethylene” was only three genes after 2 months of storage but increased dramatically over the next 7 months (Figure 2; Table S6). Heatmaps showing the expression patterns of these gene sets are available in Figure S4 (“n‐degron”) and Figure S5 (“ethylene”).

Detailed GO functional enrichment results are available in Table S7. To summarize, the genes defined as “n‐degron” have top terms (*p*‐value < .00005) at each time point related to decreased oxygen and hypoxia response (GO:0036293, GO:0070482, GO:0015979, GO:0001666, and GO:1901700). Other terms included those related to energy metabolism (GO:0006091, GO:0019684, GO:0009767, GO:0009773, and GO:0009765) and stress (GO:0080135, GO:0006970, and GO:0009651). For ethylene, there were no terms enriched for the first two time points (2 and 4 months), but later time points were enriched for terms related to ethylene (GO:0009873 and GO:0071369), low oxygen response (GO:1901700), and a variety of other signaling pathways (Table S7).

### 2.3 Putative apple PCO gene family phylogeny and expression patterns

**FIGURE 3:**
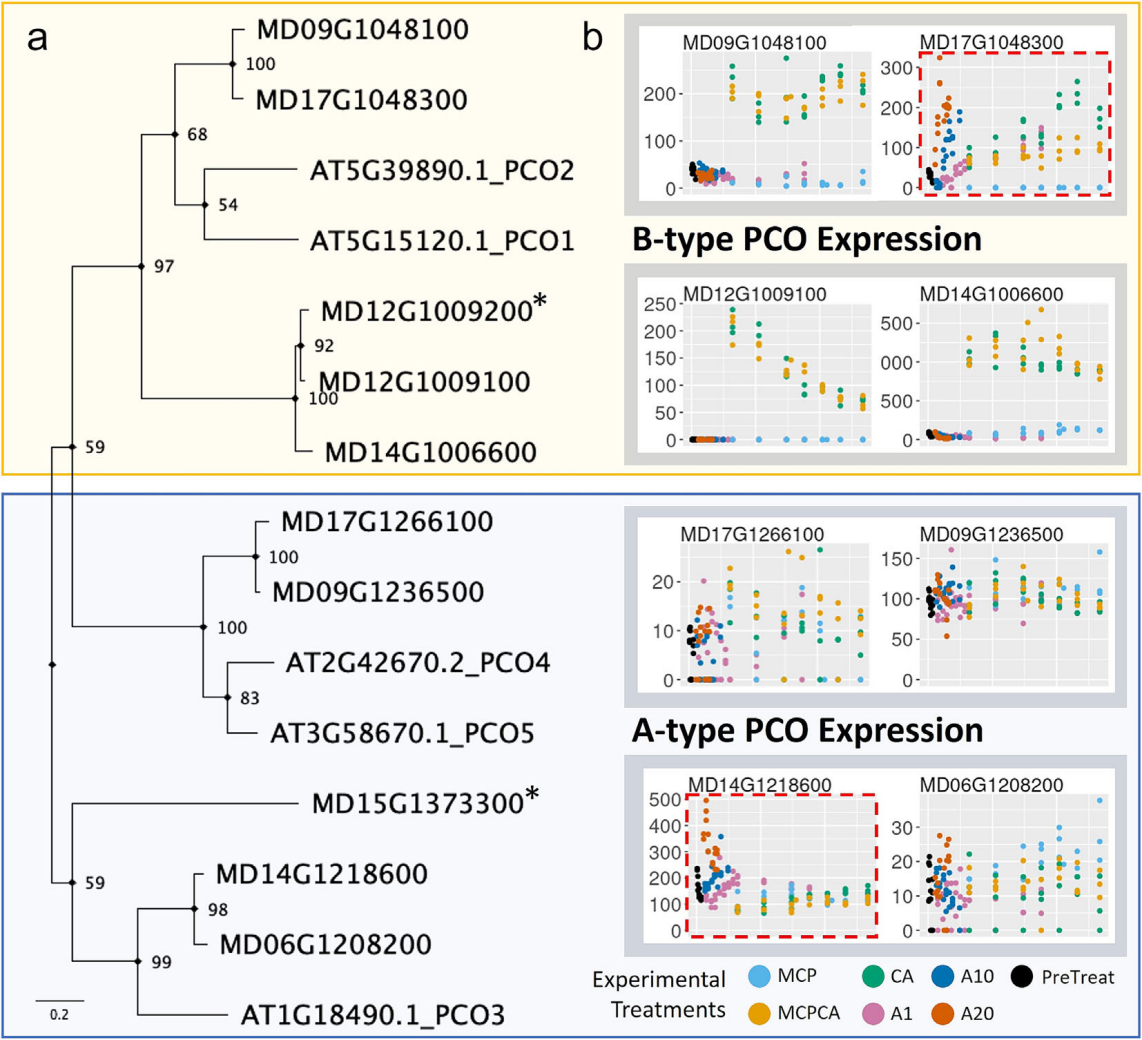
Plant cysteine oxidase (PCO) family genes in the GDDH13 apple genome can be grouped into A‐type (stable, non‐hypoxia inducible) and B‐type (hypoxia‐responsive) categories, as proposed by Weits et al. (2023). (a) A simplified gene phylogeny shows a nucleotide‐based grouping of *Arabidopsis thaliana* PCO genes and putative apple GDDH13 PCO genes. An expanded phylogeny is included in Figure 6 and includes PCO genes from 41 species. GDDH13 gene accession IDs noted with an asterisk (MD15G1373300 and MD12G1009200) had no reads mapped to them in our dataset, and thus were excluded from expression‐based analyses. (b) Gene expression of putative apple GDDH13 PCO genes across our full dataset. The *x*‐axis represents time in storage, and the *y*‐axis represents gene count, normalized using the DESeq2 package (Love et al., 2014). Putative in‐paralog pairs are surrounded by gray boxes. Paralogous pairs were also observed in other apple genomes and in another Malinae species (*Pyrus betufolia*) (Figure S6), consistent with the proposed whole genome duplication in the common ancestor of the Malinae subtribe (Li et al., 2019). Genes with unexpected transcriptomic levels based on PCO Type‐A/B classification (MD14G1218600 and MD17G1048300) are highlighted using a red dotted outline. For additional details regarding MD12G1009200, please consult the Supplemental Methods. Abbreviations: PreTreat, Pre Treatment; A1, Air, 1 °C; A10, Air, 10 °C; A20, Air, 20 °C; MCP, Air, 1 °C + 1‐MCP treatment; CA, Controlled Atmosphere 1 °C; and MCPCA, Controlled Atmosphere + 1‐MCP Treatment 1 °C.

In Arabidopsis, PCOs act in combination with constitutively expressed ERF VII proteins to regulate a “tunable oxygen‐sensing system” (Gasch et al., 2016; Weits et al., 2014), due to their ability to detect oxygen concentration and their role in the n‐degron pathway (Giuntoli & Perata, 2018). Using PlantTribes2 (Wafula et al., 2023), we identified 10 putative apple PCO homologs in the GDDH13 apple genome (Daccord et al., 2017) (Figure 3a; Figure S6). These apple PCO homologs were categorized into A‐type (not upregulated by hypoxia) and B‐type (hypoxia inducible), as proposed by Weits et al. (2023) based on phylogeny and HRPE elements (Figure 3a; Figure S6). Five apple homologs identified were characterized as A‐type PCOs (MD06G1208200, MD14G1218600, MD09G1236500, MD15G1373300, and MD17G1266100), and five were characterized as B‐type PCOs (MD14G1006600, MD12G1009100, MD09G1048100, MD12G1009200, and MD17G1048300) (Figure 3a). All B‐type apple PCO genes contained a *cis* HRPE motif in the 1000‐bp upstream of their translational start site, whereas none of the A‐type apple PCO genes contained this motif (Data S1), consistent with Weits et al. (2023). We further examined the PCO phylogeny (Figure S1) and, consistent with the recent Maleae (apple tribe) genome duplication event (Li et al., 2019), identified four likely paralogous pairs: MD06G1208200 with MD14G1218600 (A‐type) and MD12G1009100 with MD14G1006600 (B‐Type), and MD09G1236500 with MD17G1266100 (A‐type), and MD09G1048100 with MD17G1048300 (B‐type) (Figure 3b). Two of these genes showed no expression in our dataset (MD15G1373300 and MD12G1009200) and were omitted from further classification.

Gene expression of eight putative apple PCO homologs were visualized across time for all samples (*n* = 143) to assess similarities and differences in expression (Figure 3b). For A‐type PCO genes, three genes (MD06G1208200, MD09G1236500, and MD17G1266100) had relatively constant expression across all experimental conditions, whereas MD14G1218600 was upregulated in fruit stored in conditions that enhanced the speed of maturation (normoxic conditions, warm temperatures—A10 and A20 treatments). All four B‐types PCO genes (MD14G1006600, MD12G1009100, MD09G1048100, and MD17G1048300) were upregulated under hypoxic treatments (CA and MCPCA) when compared to all other treatments and exhibited complete elimination of expression in the MCP treatment (normoxic conditions and ethylene perception inhibition) (Figure 3b). Interestingly, in addition to the hypoxic treatments, the B‐type PCO gene MD17G1048300 was also upregulated in normoxic treatments designed to enhance maturation in storage (A1, A10, and A20). Additionally, MD17G1048300 also exhibited increasingly higher expression over time in the CA treatment (hypoxia) compared to the MCPCA treatment (hypoxia and ethylene perception inhibition). These patterns indicate MD17G1048300 may be partially regulated via direct ethylene sensing mechanisms—a novel observation in PCO gene regulation.

### 2.4 Putative apple ERF VII gene family phylogeny and expression

**FIGURE 4:**
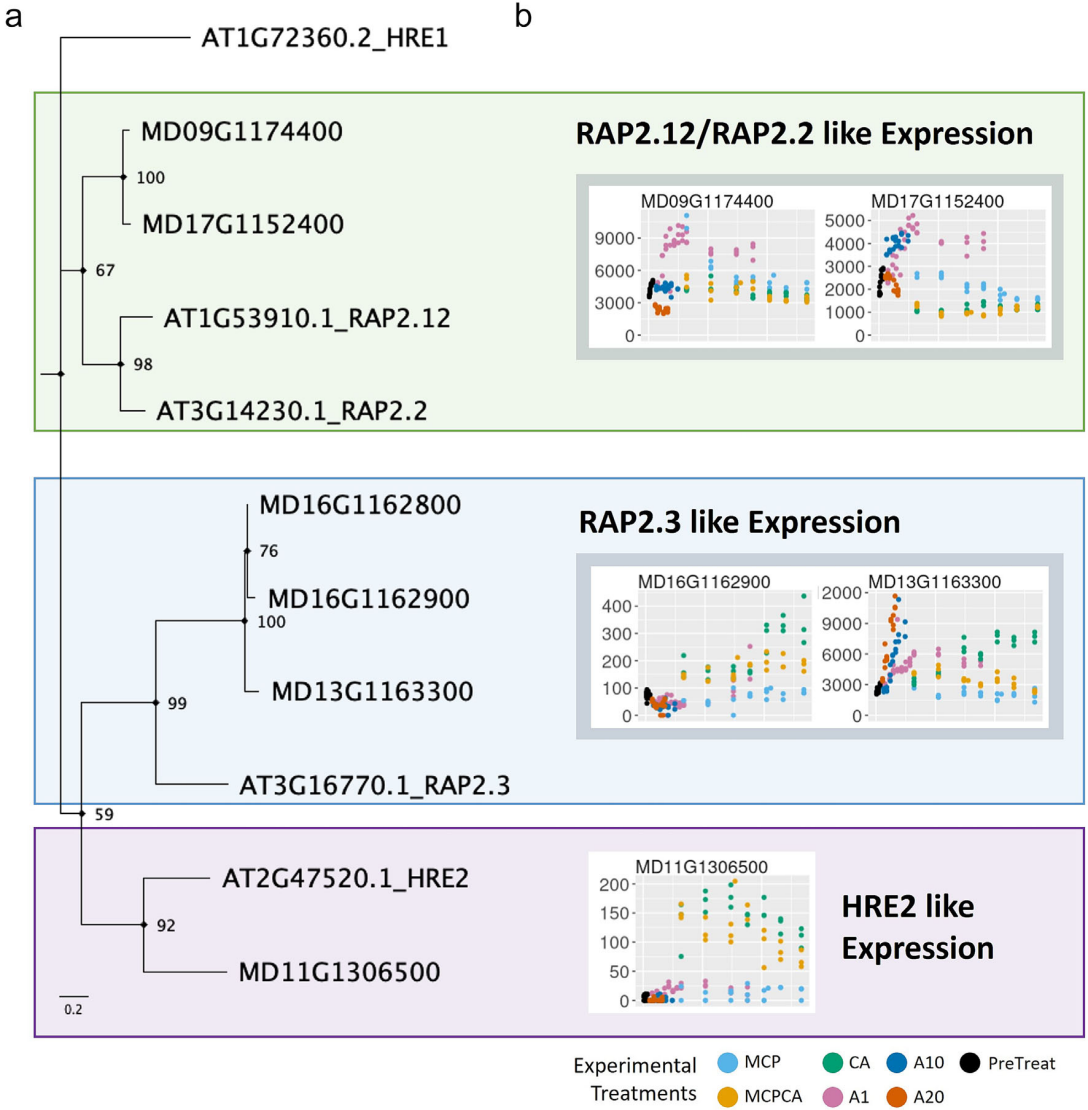
Five putative group VII ethylene response factor (ERF VII) genes were identified in the GDDH13 apple genome. (a) A simplified gene phylogeny of apple genes and their Arabidopsis homologs. Colored boxes break the phylogeny into three categories based on similarity to Arabidopsis homologs, expression patterns, and presence of *cis* HRPE motif (MD11G1306500 HRE2 like only). These are the only genes in the GDDH13 genome with the N‐terminal degron (n‐degron) motif MCGGAI/V. (b) Expression profiles of the five putative apple ERF VII genes across our entire sample set. The *x*‐axis represents time in storage, and the *y*‐axis represents gene count, normalized using the DESeq2 package (Love et al., 2014). Putative in‐paralog pairs are surrounded by gray boxes. Putative paralogous pairs were also observed in other apple genomes and in another Malinae species (*Pyrus betufolia*) (Figure S7), consistent with the proposed whole genome duplication in the common ancestor of the Malinae subtribe (Li et al., 2019). For additional details regarding MD16G1162800, please consult the Supplemental Methods. Abbreviations: PreTreat, Pre Treatment; A1, Air, 1 °C; A10, Air, 10 °C; A20, Air, 20 °C; MCP, Air, 1 °C + 1‐MCP treatment; CA, Controlled Atmosphere 1 °C; and MCPCA, Controlled Atmosphere + 1‐MCP Treatment 1 °C.

Five apple ERF VII TFs were identified in the GDDH13 genome using a phylogenetic approach (Figure 4a; Figure S7). These were categorized into three groups, RAP2.12/RAP2.2‐like, RAP2.3‐like, and HRE2‐like, based on gene family homology, expression patterns (constitutive expression or hypoxia responsive), and presence of cis HRPE motif. Two apple homologs were classified as RAP2.12/RAP2.2‐like (MD09G1174400 and MD17G1152400), two as RAP2.3‐like (MD13G1163300, MD16G1162900), and the remaining gene as HRE2‐like (MD11G1306500). These five putative apple ERF VII TFs were the only genes in the entire GDDH13 genome that contained the highly conserved N‐terminal degron (n‐degron) motif MCGGAI/V (Data S1). Specifically, this motif is used across the plant kingdom for degradation via the “type I” PRT6 cys/arg branch of the n‐degron pathway (Dissmeyer, 2019; Gibbs et al., 2011; Licausi et al., 2011).

Expression of these five putative ERF VII TFs is visualized across time for all samples in our dataset (Figure 4b). Apple RAP2.12/RAP2.2‐like (MD17G1152400; Figure 4b) showed dramatic changes in expression over time in short‐term fruit (A1, A10, and A20), with low temperature (A1) causing higher transcriptomic upregulation during prolonged storage than in hypoxic conditions (MCPCA and CA) which were also stored at the same temperature. Apple RAP2.3‐like gene MD16G1162900 showed modest increases in expression over time, while in hypoxic conditions, however, this response was muted by MCP as duration in CA continued (Figure 4b). Expression in the other RAP2.3‐like gene, MD13G1163300, increased in all conditions designed to expedite fruit maturation (A1, A10, and A20). In addition, MD13G1163300's response to hypoxia was inhibited by 1‐MCP application. RAP2.3 is a known, important regulator of ethylene signaling (Kim et al., 2018). Gene expression patterns in our experiment indicate putative apple RAP2.3 MD13G1163300 is indeed involved in ethylene sensing and signaling pathways related to both fruit ripening and response to hypoxia. The application of 1‐MCP appears to suppress expression of this particular gene, indicating that 1‐MCP interferes in ethylene mediated response to hypoxia. Interestingly, the other putative apple RAP2.3, MD16G1162900, appears to be involved specifically in ethylene signaling in response to hypoxia, but not ripening, and its activity does not appear to be inhibited by 1‐MCP application until very late in the postharvest storage period (> 6 months). Apple HRE2‐like gene MD11G1306500 (Figure 4b) was highly upregulated during hypoxia when compared to other treatments, although sustained exposure to hypoxia led to a decrease in this elevated expression over time.

### 2.5 TF networks reveal potential novel regulators of apple hypoxia response

**FIGURE 5:**
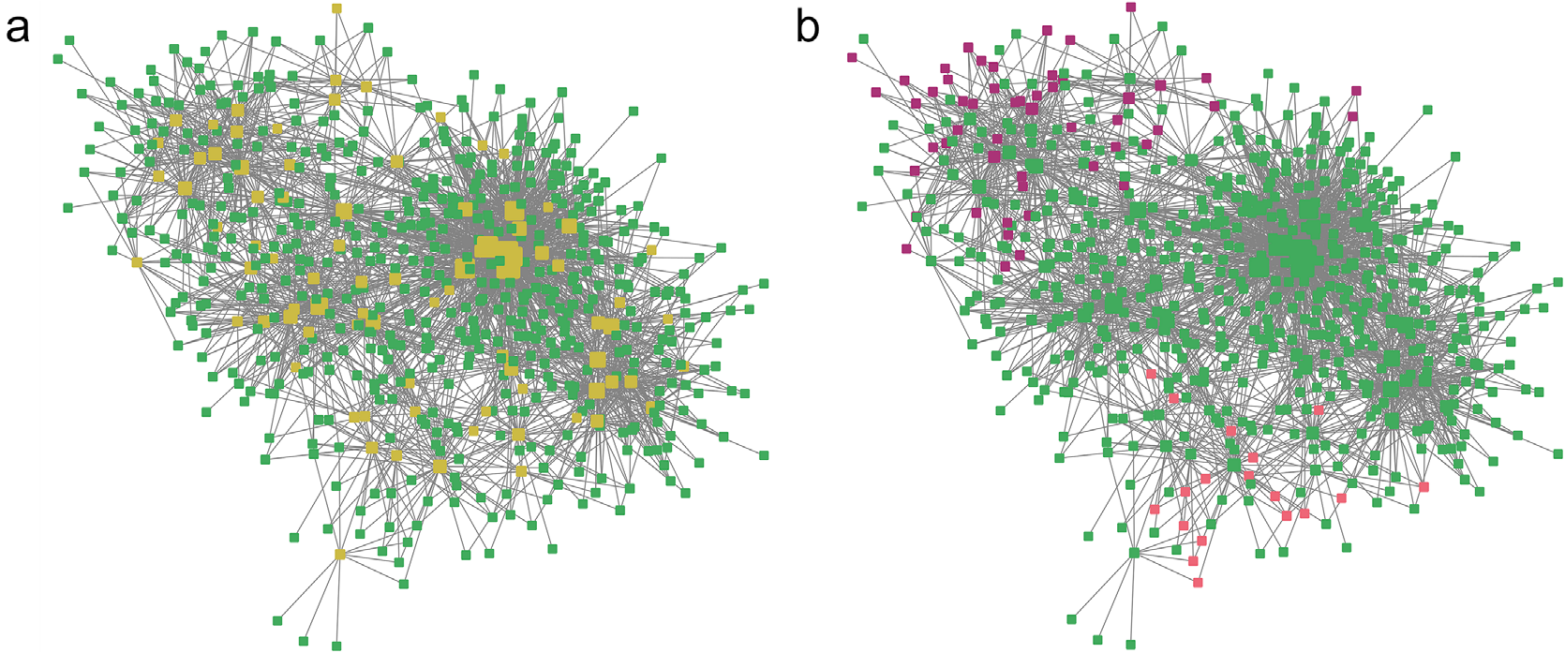
The transcription factor regulatory network of the putative apple hypoxia response genes. (a) Apple transcription factor regulatory network of the 606 putative hypoxia genes (green) and the 67 predicted transcription factors (TFs) within the network (yellow). Node size is relative to node degree (degree is the number of edges connected to the TF which indicates the number of hypoxia genes they are predicted to regulate). (b) The same base network as a with genes highlighted as those observed as up‐regulated only in the hypoxia upregulated CA only subset (purple) and the hypoxia upregulated MCPCA only subset (pink). CA, controlled atmosphere; MCPCA, controlled atmosphere + 1‐MCP Treatment.

A TF regulatory network was created using GENIE3 (Huynh‐Thu et al., 2010). Of the 1557 apple TFs with expression in the full network, 67 were present within the putative hypoxia regulatory network (Figure 5a; Table S8). In the putative hypoxia response network, genes from the hypoxia upregulated CA and MCPCA subsets formed respective modules in the regulatory network. This provides more evidence of coregulation among genes within each subset (Figure 5b).

**FIGURE 6:**
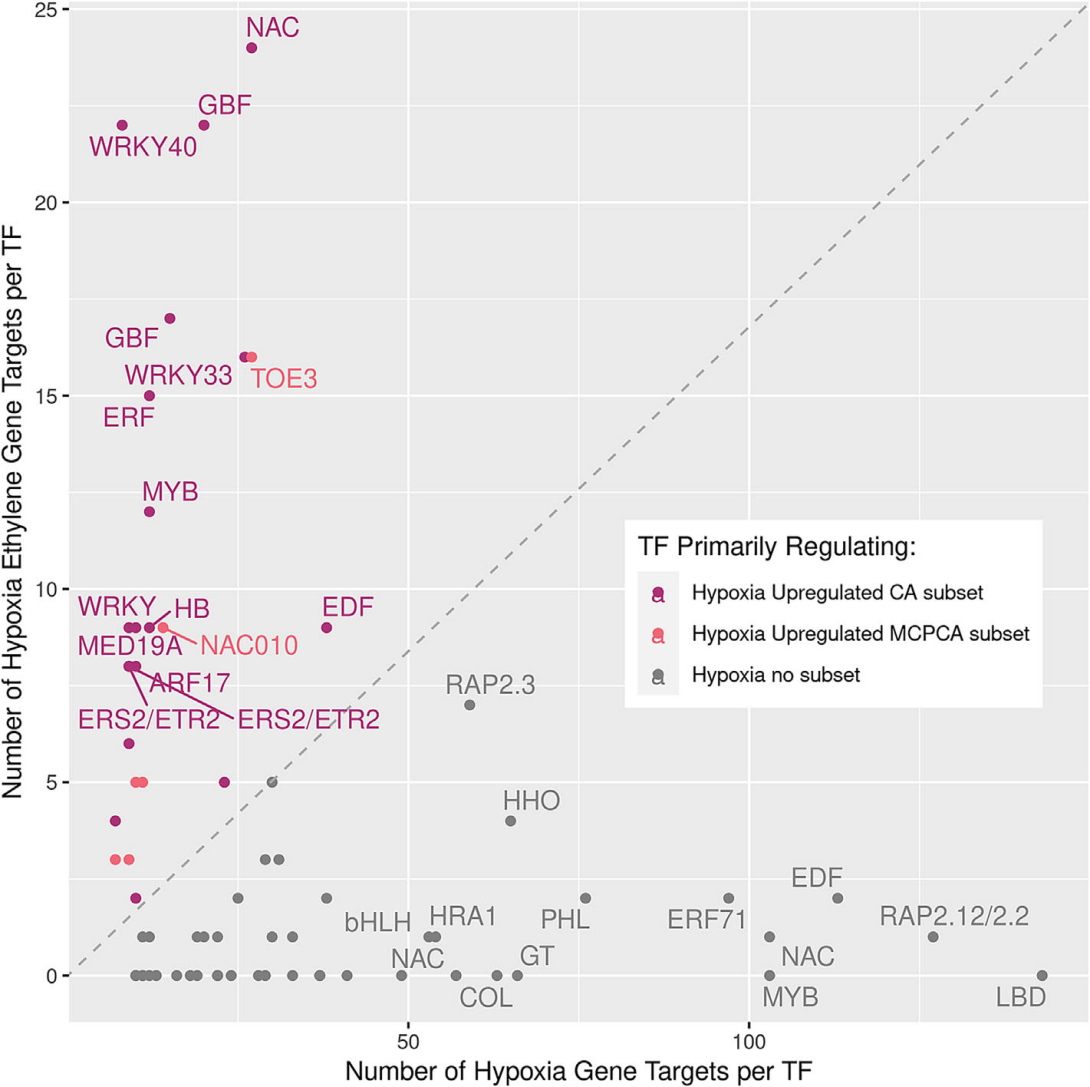
Number of gene targets for each of the 67 putative transcription factors. The number of gene targets for each of the 67 putative transcription factors (TFs) identified to regulate differentially expressed hypoxia genes. The *x*‐ and *y*‐axes are scaled to be the same length, and a dashed line is placed on the diagonal between them (line is *y* = 24/143 * *x*). A TF's position along the *x*‐axis is determined by how many of the 606 hypoxia each TF is expected to target, and position along the *y*‐axis is determined by how many of the hypoxia upregulated CA and MCPCA subsets they are expected to target. The hypoxia upregulated CA and MCPCA subsets are the genes which were found to be responsive to hypoxia, but only in either the CA or MCPCA treated fruit. TFs are considered to not have an ethylene component if they fall below the line (i.e., their MCPCA and CA hypoxia response is similar) and are expected to have an ethylene component if they fall above the dashed line. Each TF is colored based on the differential gene expression (DE) group it was upregulated in. TFs responsive to hypoxia, but not a member of either the hypoxia upregulated CA or MCPCA subsets, are colored gray. TFs predicted to regulate the hypoxia upregulated CA only subset are colored purple, and TFs predicted to regulate the hypoxia upregulated MCPCA only subset are colored pink. Arabidopsis homolog gene names are annotated for TFs regulating more than 50 “standard hypoxia” genes or more than seven “:ethylene mediated hypoxia” genes (hypoxia upregulated CA and MCPCA subsets). Note, in some instances, multiple apple homologs are present for an individual Arabidopsis gene name (i.e. WRKY33 and GBF3). See Table S9 for a detailed description of each of the 67 TFs in the plot. CA, controlled atmosphere; MCPCA, controlled atmosphere + 1‐MCP Treatment.

To visualize which TFs are responsible for regulating these different subsets of the hypoxia response, Figure 6 was created. Figure 6 is a scatter plot of the 67 TFs identified as regulating some aspect of the apple hypoxia response. Their position on the plot is based on how many genes belonging to each of the hypoxia subsets they putatively regulate. The *x*‐axis represents how many of the 606 hypoxia genes each of the TFs is predicted to regulate, and the *y*‐axis represents the TF factors which regulate the hypoxia upregulated CA and MCPCA subsets of genes. TFs which regulate primarily the subset are colored as purple, and those which primarily regulate the hypoxia upregulated MCPCA subset are colored as pink. TFs further to the right of Figure 6 are regulated similarly with or without ethylene, whereas TFs closer to the top regulate genes in the hypoxia upregulated CA and MCPCA subsets. Because the hypoxia upregulated CA and MCPCA subsets have an ethylene component, we suspected that the TFs regulating them would also have an ethylene component. Additional information about the TFs visualized in Figure 6, such as similarity to Arabidopsis genes, number of genes regulated, and putative function, can be found in Table S9.

Several of the TFs identified in the regulatory network have been previously implicated in the hypoxia response in Arabidopsis. Putative TFs regulating the full 606 DEG hypoxia gene set (Figure 5a) are associated with known hypoxia‐related TFs such as apple gene MD09G1088700, which is in the same gene family as Arabidopsis LOB domain containing proteins such as LBD41 (though no direct homology could be inferred between MD09G1088700 and any Arabidopsis genes in the gene family), RAV1 (MD13G1046100), and HRA1 (Giuntoli, Licausi, et al., 2017) (MD14G1094300). In addition, three of the ERF VII TFs previously mentioned were also identified: HRE2 (MD11G1306500), RAP2.12 (MD17G1152400), and RAP2.3 (MD16G1162900) (Giuntoli & Perata, 2018).

TFs that regulate hypoxia upregulated CA and MCPCA subsets (Figure 5b; Figure 6) included TFs known to be involved in cross‐talk between ethylene and stress‐related pathways such as potential apple homologs of ERF1 (MD10G1184800) and ETR2 (MD13G1209700) (Hartman et al., 2019; Zhao et al., 2012), TEM1 (MD16G1047700), and TOE3 (an AP2 domain containing protein) (MD15G1286400) (Licausi et al., 2013), as well as WRKY33 (MD04G1167700 and MD12G1181000) (Tang et al., 2021). Interestingly, one TF, MD13G1209700 (ETR2 homolog), was predicted to regulated members of both the hypoxia upregulated CA subset (8 targets) and the MCPCA subset (two targets). Additionally, several additional putative regulators not extensively implicated in the hypoxia response were identified in our network, including members of the NAC family proteins which have been implicated in plant stress responses (Bian et al., 2020) and fruit ripening and softening (Forlani et al., 2021; Qi et al., 2022; Watts et al., 2023), including ripening and softening processes that are independent of ethylene sensing and signaling (Migicovsky et al., 2021). Other putative regulators include members of the MYB family which are known to be involved in cell morphology and primary and secondary metabolism (Cao, Li, et al., 2020), and additional members of the WRKY family which are involved in stress responses and developmental processes (Chen et al., 2017). These additional TFs may be mediators of the hypoxia response in apple fruit which are potentially working in conjunction with currently identified mechanisms.

### 2.6 TF networks reveal in‐paralogs of putative apple ERF VII family genes have separate regulatory networks and functional roles

**FIGURE 7:**
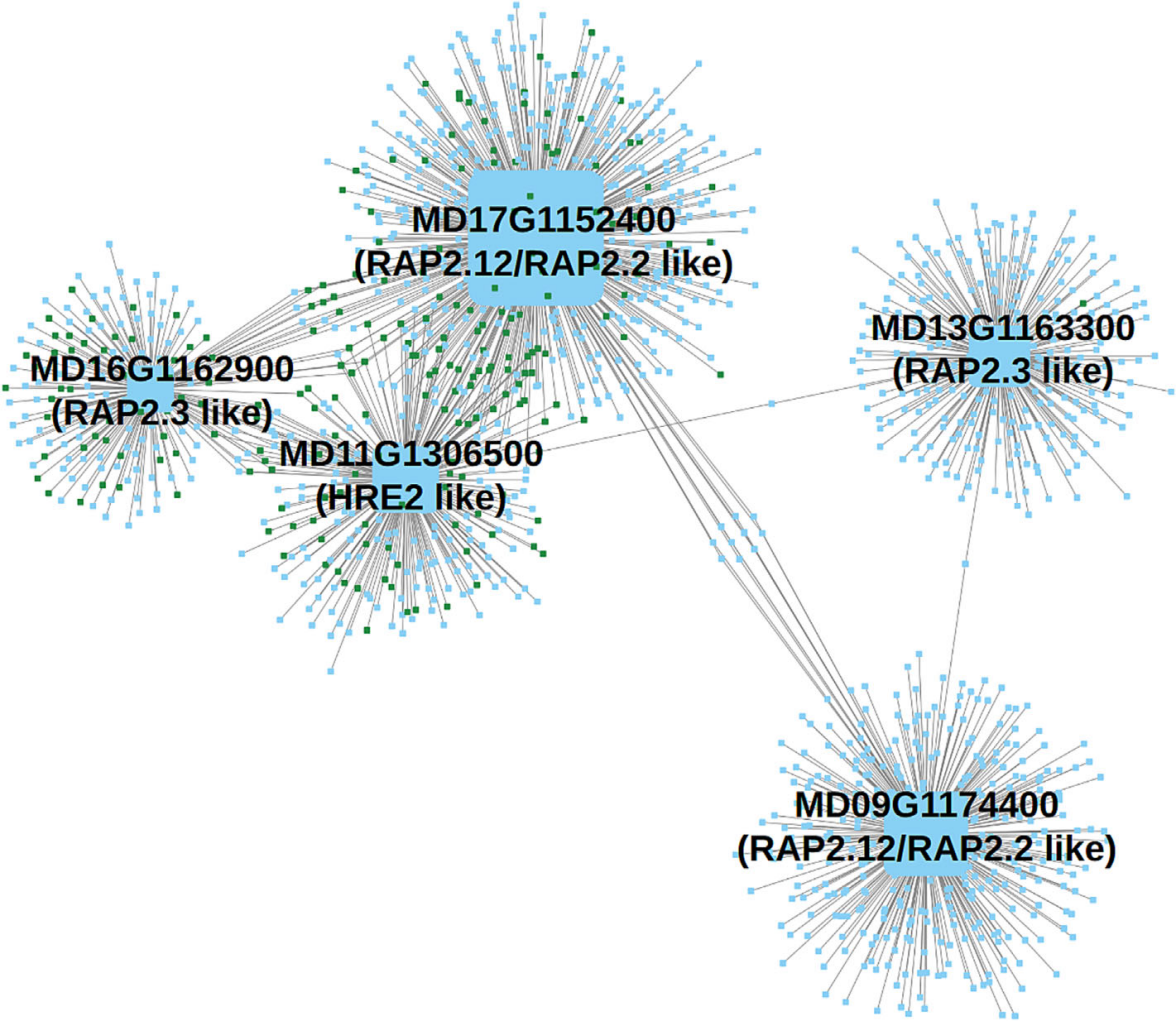
Putative apple ERF VII transcription family network indicates each ERF VII TF regulates different pools of genes with minor overlap. ERF VII genes are sized by total degree and annotated. Green‐colored nodes are those also identified in the DEG 606 hypoxia set. This subgraph highlights two major results: there is little overlap between genes putatively regulated by each ERF VII TF, and three of these TFs are predicted to control genes from the 606 hypoxia set, while the other two duplicates do not. ERF VII, group VII ethylene response factor; TF, transcription factor.

The varied responses of apple ERF VII TF expression patterns seen in Figure 4 suggest they may regulate different gene sets. To explore these gene sets of apple ERF VII TFs, a reduced network was extracted from the full regulatory network (Figure 7; Table S10 **)**. The five apple ERF VII TFs were among the most active TFs in the full network, with 356 genes predicted to be regulated by MD09G1174400, 588 by MD17G1152400, 253 by MD13G1163300, 184 by MD16G1162900, and 273 by MD11G1306500. In contrast, the other TFs in the network only regulated 77 genes on average.

However, our experiment showed that each ERF VII gene is putatively regulating a different pool of hypoxia response genes which have limited overlap between them. There was minor overlap in the predicted targets for three of the ERF VII TFs—MD17G1152400 (RAP2.12/2.2‐like), MD11G1306500 (HRE2‐like), and MD16G1162900 (RAP2.3‐like), and even less overlap for those genes identified as being putative hypoxia response genes (Figure 7, green nodes). Only five genes were shared across all three of the aforementioned TFs, with two of the five also being a part of the hypoxia response DEG subset. There were 69 genes regulated by both MD11G1306500 and MD17G1152400 (only two were a part of the hypoxia response subset), 18 by MD11G1306500 and MD16G1162900, and 12 by MD16G1162900 and MD17G1152400 (Table S10). The putative RAP2.12/2.2‐like in‐paralogs (MD09G1174400 and MD17G1152400, Figure 4a) and RAP2.3‐like in‐paralogs (MD13G1163300 and MD16G1162900) had little to no functional overlap between the in‐paralogs. Notably, these in‐paralogs also did not have any putative targets identified that were a part of the 606 putative hypoxia response genes. This small amount of overlap between ERF VII genes is interesting, as it indicates apple ERF VII genes are potentially acting on different gene subsets in response to hypoxia and ethylene.

In addition to there being little to no overlap in targets between ERF VII in‐paralogs, there was little functional overlap observed in the GO enrichment analysis (Table S11). The two pools of genes regulated by the putative in‐paralog RAP2.2/RAP2.12 TFs were enriched for different functional terms, with MD09G1174400 enriched for terms related to cellular components (i.e. “envelope,” “organelle part,” “chloroplast envelope”), while MD17G1152400 was enriched for oxygen‐related terms and stress responses (i.e., response to “abiotic stimuli,” “response to hypoxia,” and “response to chemical”). Genes regulated by putative RAP2.3 TF MD13G1163300 were enriched for only one term—organ morphogenesis (GO:0009887). Genes regulated by putative RAP2.3 TF MD16G1162900 had no enriched terms. The putative HRE2‐related TF MD11G1306500 was enriched for three low oxygen‐related terms (GO:0070482, GO:0036293, and GO:0001666).

## 3 DISCUSSION

Postharvest treatments such as chilling, CA, and 1‐MCP are important tools used for long‐term storage of apples. However, the molecular mechanisms for why these methods are effective are not well understood in apple. This study investigated the time‐series transcriptomic response of apples under six different postharvest storage regimes. We sought to identify the core hypoxic response in apples, gain insight into how ethylene perception can impact the hypoxia response, and suggest homolog‐specific adaptations to the current hypoxia molecular model (Giuntoli & Perata, 2018).

### 3.1 Evidence for neo‐functionalization of core hypoxia pathway genes in apple fruit

**FIGURE 8:**
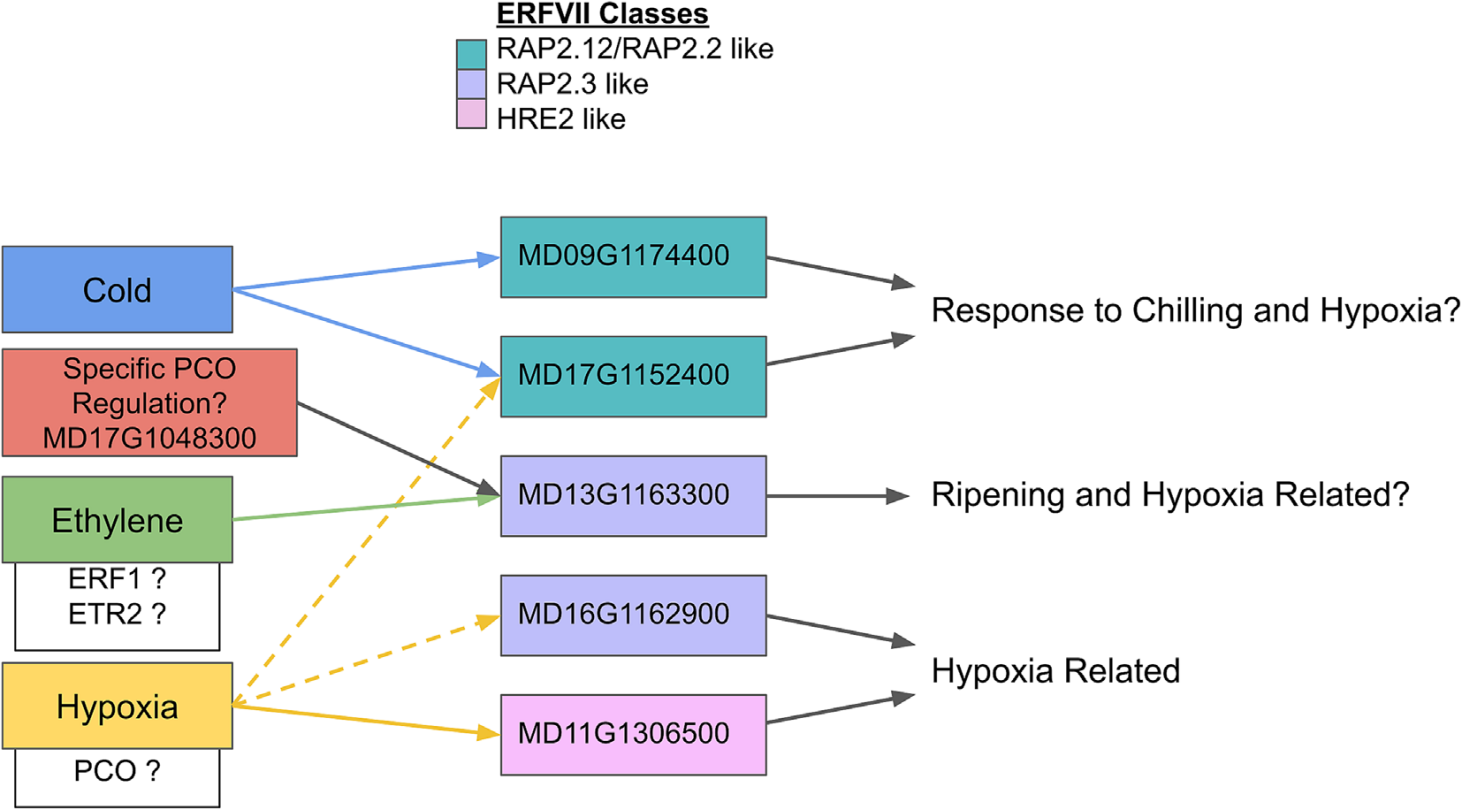
Putative diagram of transcriptomic regulation of ERF VII transcription factors in apple. Regulators of transcription are cold, PCO, ethylene, and hypoxia. While PCO acts post‐translationally on ERF VII, upregulation of PCO's at the transcription level by ERF VII results in a transcriptional feedback loop. ERF VII, group VII ethylene response factor; PCO, plant cysteine oxidase.

We observed apparent neo‐functionalization of the core n‐degron pathway genes, specifically ERF VII and PCO, at the transcriptomic level during postharvest storage. We chose to focus on the n‐degron pathway genes due to their importance in regulating downstream hypoxia TFs (ERF VII family) and the direct sensing of oxygen (PCO family). The putative apple PCO family classified here consists of eight active homologs of the five Arabidopsis PCOs. These eight genes consist of four in‐paralog pairs that are likely the result of the Malinae duplication event (Li et al., 2019), with two pairs belonging to the A‐type PCOs and two pairs belonging to the B‐type PCOs (Weits et al., 2023). Previous work has shown that transcriptomic levels of B‐type PCO genes are directly induced by ERF VII proteins binding the *cis* HRPE motif during hypoxic conditions, whereas A‐type PCO genes are constitutively expressed at low levels (Gasch et al., 2016; Weits et al., 2023). The induction of the B‐type PCO on exposure to hypoxic conditions “primes” a negative feedback loop where the increased levels of B‐type PCO proteins can rapidly reduce the levels of ERF VII TFs once the plant returns to normoxic conditions. This rapid attenuation of the hypoxia response is important to ensure that the expensive anaerobic metabolism is substituted for the more efficient aerobic metabolism (van Dongen & Licausi, 2015). Our data verified this response for three of the four active B‐type apple PCO genes (MD14G1006600, MD12G1009100, and MD09G1048100), but the response of the fourth gene (MD17G1048300) was unexpected. MD17G1048300 showed a response to hypoxia, but its ability to respond to hypoxic conditions was hindered by 1‐MCP. Such an impact indicates that MD17G1048300 utilizes ethylene sensing as a part of its response to hypoxia, which is a novel observation in PCO gene regulation. This observation suggests a potential neo‐functionalization of B‐type PCO‐like genes in apple, which likely arose after the Malinae duplication event and is supported by our phylogenetic analysis (Li et al., 2019; Figure S6). Previous studies of Arabidopsis PCOs have shown preferential selectivity of different ERF VII TFs and differences in expression patterns indicating the groups are not completely homogenous in function (White et al., 2018). For apple putative B‐type PCO MD17G1048300, the presence of the *cis* HRPE binding motif, similarities in expression patterns to one of the ERF VII RAP2.3 orthologues (MD13G1163300), and putative regulatory control support the hypothesis that these two genes' response to hypoxia includes an ethylene sensing component (Figure 8).

MD14G1218600, an A‐type PCO, also showed unexpected expression patterns compared to previously described A‐type PCOs (Weits et al., 2023). It is the most highly expressed A‐Type PCO gene in our dataset and is upregulated in fruit stored in air and in warmer conditions (A10 and A20), and to a lesser extent, fruit stored in air in cold storage (A1). The pattern of gene activity across all three treatments follows that of ripening stages. In the A10 condition, expression increases over time as fruit gradually ripen. In the A1 condition, we see a similar pattern, except that towards the end of the time course, expression appears to taper and begin to fall. This is congruent with climacteric ripening patterns, where ripening progresses to climacteric ethylene burst, followed by a cascade of molecular processes (Giovannoni, 2001; Payasi & Sanwal, 2010; Prasanna et al., 2007). Such a switch is most apparent in the A20 treatment, where gene expression starts out very high, and then slowly decreases as fruit progress through later stages of ripening. Importantly, in conditions where ethylene signaling and ripening processes are impeded (MCP, CA, MCPCA), there is a notable lack of upregulation of this gene. All together, these observations indicate MD14G1218600 may have an ethylene sensing and signaling component associated with fruit ripening, a novel role and regulatory feature of A‐type PCOs. This gene shares homology with putative A‐type PCO MD06G1208200, likely as a result of the Malinae genome duplication event. It is possible that this duplication led to neo‐functionalization of this putative A‐type PCO for ripening related processes.

The apple ERF VII TF gene family has a wide variety of responses to postharvest treatments at the transcriptomic level (Figure 4). Upregulation occurs during chilling for RAP2.12 and RAP2.2‐like apple genes (MD09G1174400 and MD17G1152400) whereas one of the two RAP2.3‐like genes (MD13G1163300) was upregulated during ripening and hypoxia conditions. The other RAP2.3‐like gene (MD16G1162900) and the HRE‐like gene (MD11G1306500) showed a more typical hypoxia upregulation response. The behavior of RAP2.12, RAP2.2, and RAP2.3‐like responding to chilling and ripening has not been recorded in apples before this study. However, transcriptomic control of ERF VII genes during stress and developmental conditions has been recorded and verified in Arabidopsis models (Giuntoli, Shukla, et al., 2017; Giuntoli & Perata, 2018). Confirmed Arabidopsis responses include RAP2.2 being upregulated by ethylene to induce Botrytis resistance (Giuntoli & Perata, 2018; Zhao et al., 2012), and RAP2.3 being upregulated by ethylene and downregulated by DELLA to mediate apical hook development (Marín‐de la Rosa et al., 2014). In both instances, the ERF VII TFs act to integrate signals from multiple inputs.

Each of the five putative apple ERF VII TFs are predicted to regulate different sets of genes with minor overlap (Figure 7). Three of the genes (MD11G1306500—HRE2‐like, MD17G1152400—RAP2.12/2.2 like, and MD16G1162900—RAP2.3‐like) were observed in our predicted regulatory network to regulate the majority of the hypoxia response genes, with some overlap in targets. In‐paralogs of RAP2.12/2.2‐like genes (MD09G1174400 and MD17G1152400) and RAP 2.3‐like genes (MD13G1163300 and MD16G1162900), however, have little to no overlap with the other three ERF VII TFs and are predicted to regulate dramatically different, nonhypoxia‐related gene targets. While this transcriptomic data is suggestive of novel new roles of ERF VII and PCO apple genes, further investigation will be needed to verify that they are functionally active at the protein level and at relevant time scales, for which feasible tools do not yet exist for this study system.

### 3.2 Expansion of the downstream hypoxia response gene set

Our DE analysis identified 606 genes being upregulated in response to hypoxic conditions (Figure 1). These genes had a variety of functional classifications, with the most significantly represented GO terms related to oxygen sensing, rerouting of metabolism, and energy management (Table S3). This is consistent with previous studies (Cukrov et al., 2016; Mustroph et al., 2009), with our DEG set covering half of the genes identified as “core hypoxia” in Arabidopsis from Mustroph et al. (2009) (Figure S3 **)**. In addition, the MCPCA treatment allowed us to look at the set of hypoxia related DEGs upregulated with respect to ethylene sensing and signaling by comparing to the CA only treatment. This list of DE genes was much smaller (72 in total), but consisted of genes that are responsive to ethylene in other experiments such as ACC OXIDASE (ACO1)ACC SYNTHASE (ACS10) (Cukrov et al., 2016; Ireland et al., 2014), ERF1 and ERF2 (Hartman et al., 2019), and HYPOXIA RESPONSE UNKNOWN PROTEIN 26 (HUP26) (Table S2).

The TF regulatory network was thresholded using the set of 606 differentially expressed hypoxia genes to identify potential TFs, with a total of 67 TF being identified (Figure 5a). Several of these TFs are known regulators of aspects of the plant hypoxia response. This included ERF VII genes which directly induce a hypoxia response, and genes that co‐regulate with or are induced by the ERF VII genes, to initiate downstream hypoxia responses (Table S9). Examples include MD09G1088700, which is in the same gene family as LBD41 (AT3G02550), an anaerobic metabolism regulator (Mustroph et al., 2010); HRA1 (MD14G1094300), a mediator of the hypoxia response downstream of ERF VII proteins (Giuntoli et al., 2014; Giuntoli, Shukla, et al., 2017); and MD13G1046100 and MD16G1047700 which are both orthologous to RAV1 (AT1G13260), a regulator of plant growth during stress conditions (Sengupta et al., 2020). The hypoxia upregulated CA subset of DEGs (gene set of 72) had predicted TF regulators which are induced by ethylene, such as members of the AP2/ERF family, including ERF 1 and EDF3 ( Licausi et al., 2013, Müller & Munné‐Bosch, 2015), WRKY member TFs (Jing et al., 2021), and MYBs (Cao, Chen, et al., 2020; Zhang et al., 2019). These TFs are also implicated in cross regulation of hormones for many stress responses in Arabidopsis (Zhao et al., 2012; Marín‐de la Rosa et al., 2014; Waadt et al., 2022), which could help explain why some ERF VII TFs normally associated with the hypoxia response also showed a response to cold stress and prolonged storage duration (Figure 2). The regulatory networks presented here identified genes and TFs known to be involved in the hypoxia response using only RNA‐seq data. This ability to identify relevant genes without previous knowledge of function or role demonstrates how RNA‐seq networks can be a valuable tool for exploring pathways in non‐model organisms. These networks guide hypotheses for additional TFs involved in the apple hypoxia response.

### 3.3 Ethylene sensing and signaling impacts the fruit transcriptome response to hypoxic conditions many months into postharvest storage

**FIGURE 9:**
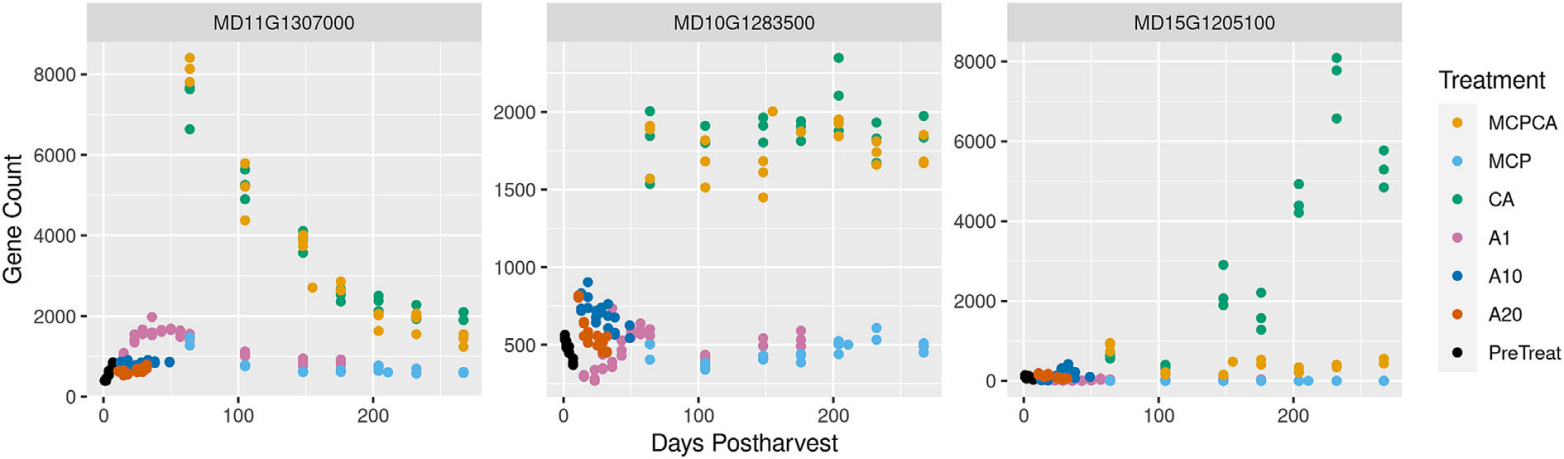
Representative model of three categories of hypoxia response genes in apple. A and B are included in the “n‐degron” response group because they are induced by hypoxia (MCPCA or CA) regardless of the ability to perceive ethylene, whereas the C is referred to as “ethylene” as it is induced in CA but not in treatments using MCP. A is rapidly induced then decreases with time, B is constitutively expressed, and C is upregulated late in the treatment, but only in CA fruit. Example genes shown in this figure are MD11G1307000 an ortholog of AT4G02280 (SUS3), a sucrose synthase; MD10G1283500, no Arabidopsis ortholog based on phylogeny; and MD15G1205100, an ortholog of AT2G19590 with sequence similarity to ACC oxidase 1 (ACO1), a critical enzyme in ethylene biosynthesis which requires oxygen availability. Abbreviations: PreTreat, Pre Treatment; A1, Air, 1 °C; A10, Air, 10 °C; A20, Air, 20 °C; MCP, Air, 1 °C + 1‐MCP treatment; CA, Controlled Atmosphere 1 °C; and MCPCA, Controlled Atmosphere + 1‐MCP Treatment 1 °C.

Hypoxia experiments performed in model organisms such as Arabidopsis and rice have focused on short‐term low‐oxygen environments from a few hours to a few days (Ellis et al., 1999; Klecker et al., 2014; Mustroph et al., 2009), because longer exposure results in tissue death. Apple fruit, in contrast, can be stored for periods of up to a year in low oxygen conditions (Gapper et al., 2022). The ability to withstand months of hypoxia exposure may stem from novel adaptations during fleshy fruit evolution for maintaining desirability for seed dispersers (Dardick & Callahan, 2014; Liu et al., 2020; Xiang et al., 2017) and compensating for natural hypoxic niches that develop in large storage organs (Geigenberger et al., 2000; Rolletschek et al., 2002; Ho et al., 2008, 2011; Licausi et al., 2011; Cukrov, 2018; Loreti & Perata, 2020). Our results indicate that during long‐term, low‐oxygen storage, apple fruit transcriptome responses can be split into at least two categories: (1) an early response which is putatively controlled by the n‐degron pathway (Figure 2; Figure 9a,b), where fewer genes are upregulated as time in storage progresses, and (2) a late response which is putatively controlled by ethylene sensing and signaling (Figure 2; Figure 9c), where an increasing number of genes are upregulated as fruit spend more time in hypoxic conditions. The early response can be further split into two subcategories, one where expression is sustained over the course of long‐term CA storage (Figure 9b) or one where expression decreases after initial induction (Figure 9a). These patterns can also be seen in the heatmap clusters in Figure 1.

In conclusion, transcriptome regulation response to hypoxic conditions is dynamic in fruit stored in low oxygen conditions for long periods of time (up to 9 months in this work). Hundreds of genes are upregulated in “Gala” apple in response to hypoxic conditions, and differences in transcriptome profiles in response to long‐term storage conditions, including the use of the ethylene perception inhibitor 1‐MCP, suggests ethylene pathway signaling interference. The recent whole genome duplication in apple may have resulted in duplicated genes that were retained due to novel functional roles associated with hypoxia response regulatory pathways and cross talk with ethylene sensing and signaling pathways. These results shed light on where future research should be focused to understand how apple fruit quality, response to hypoxia, and ethylene perception are related to improve postharvest management practices.

## 4 EXPERIMENTAL PROCEDURES

### 4.1 Plant material, experimental design, and fruit texture

“Gala” apples were collected and sorted as detailed in Hadish et al. (2024). Briefly, the fruit was received from a commercial facility in Quincy, WA, on August 21st, 2018. Upon arrival at the USDA‐ARS Tree Fruit Research Laboratory in Wenatchee, WA, apples were randomly sorted by hand and stored in air at 1°C for 7 days. After this 7‐day conditioning period, apples were randomly assigned into six treatment condition categories (A1, A10, A20, MCP, CA, MCPCA). Fruit in the MCP and MCPCA treatments were treated with 1μL/L SmartFresh™ (AgroFresh Solutions, Inc., Philadelphia, PA USA), also known as 1‐MCP, overnight and then stored at 1°C in either air (MCP) or CA (MCPCA, 2% O_2_, 1% CO_2_). 1‐MCP was applied at 1°C and in accordance with SmartFresh™ product recommendations. The fruit not treated with 1‐MCP were stored at 1°C in a CA (2% O_2_, 1% CO_2_), at 1°C in air conditions (A1), at 10°C in air conditions (A10), or at 20°C in air conditions (A20). Postharvest sampling was done at condition‐relevant time intervals, as untreated fruit stored in air (A1, A10, and A20 treatments) were expected to lose fruit quality (and thus expire) faster than fruit treated with long‐term storage preservation techniques (MCP, CA, MCPCA). Fruit texture was assessed at each postharvest sampling time point using a DigiTest‐2 penetrometer (Mohr Test and Measurement LLC, Richland, WA, USA) to confirm fruit texture was maintained as expected in our experimental treatments to ensure this study's relevancy to commercial apple fruit. Consult Hadish et al. (2024) for a detailed description of experimental conditions, treatments, sampling time points, and fruit quality assessment.

### 4.2 Tissue collection, RNA extraction, and quality control

Tissue collection was performed as described in Hadish et al. (2024). Briefly, fruit were kept at treatment respective temperatures until tissue harvest. Fruit stored in CA was removed prior to tissue harvest (in air for ~30 min from removal to completion of tissue collection). Three slices of cortex from six “Gala” apples each were pooled to create a biological replicate, and three biological replicates were collected (18 apples total) for each treatment. Cortex slices were coarsely diced and immediately flash‐frozen in liquid nitrogen and stored at −80°C.

RNA was extracted using a CTAB/Chloroform protocol modified for use on pome fruit tissue in the postharvest period (Honaas & Kahn, 2017). Extracted RNA was analyzed for purity using the NanodropOne (Thermo Fisher Scientific, Waltham, MA USA), for integrity on the Agilent Bioanalzyer (Agilent, Santa Clara, CA USA, Agilent‐RNA Pico Kit, cat#: 5067‐1513), and quantity using the Invitrogen™ Qubit™3 (Thermo Fisher Scientific, Waltham, MA USA, Qubit™ RNA HS Assay Kit, cat#: Q32852). Only RNA that met the following standards was used for downstream analysis: A260/A280 ≈ 2.0, RNA Integrity Number (RIN) ≥ 8.0.

### 4.3 Transcriptome sequencing, quality control, and reference genome selection

Transcriptome sequencing, quality control, and reference genome selection were performed as described in Hadish et al. (2024). Briefly, libraries using Lexogen's QuantSeq 3′ mRNA‐Seq Library Prep Kit FWD (Cat# 015; www.lexogen.com) were prepared at the Penn State Genomics Core Facility (University Park, PA, USA) per (Honaas et al., 2019). Libraries were sequenced on a 150‐bp single‐end protocol to a target volume of ∼20 million reads per biological replicate on Illumina's HiSeq 2,500 in Rapid Mode. Raw read data are publicly available at the NCBI Sequencing Read Archive (SRA ‐ BioProject PRJNA938164).

RNA‐seq reads were preprocessed with Trimmomatic (Bolger et al., 2014) prior to genome alignment, per (Lexogen, 2020) recommendations. These reads were then processed using the GEMmaker Workflow (Hadish et al., 2022) running Hisat2 (Kim et al., 2015), using default settings to create a Gene Expression Matrix (GEM). The “Golden Delicious” doubled‐haploid genome (GDDH13) (Daccord et al., 2017) was downloaded from the Genome Database for Rosaceae (GDR) (Jung et al., 2019) and used for alignment. The GDDH13 “Golden Delicious” genome was selected over the “Gala” genome (Sun et al., 2020) due to superior UTR annotation quality (Waite et al., 2023). The GEM was normalized using DEseq2's (Love et al., 2014) median of ratio normalization (Anders & Huber, 2010). Samples with less than 20% alignment (*n* = 4) and genes with zero RNA‐Seq reads across the sample set (*n* = 14,256) were removed prior to downstream analyses. Both the GEM and MultiQC reports can be found in Hadish et al. (2024).

### 4.4 Differential expression and GO enrichment analyses

The DESeq2 package (Love et al., 2014) was used to identify DEGs. Three different DEG analyses were performed. For the first two analyses, samples were grouped by treatment. The first analysis sought to identify all genes which were upregulated during hypoxia. We chose to concentrate on upregulated genes to limit our analysis to the active hypoxia response. To be classified as upregulated during hypoxia, we set a threshold for both the log2 fold change (>1) and the Bonferroni adjusted *p*‐value (*p*adj < .05) in MCPCA and/or in CA (hypoxic conditions) compared to each of the other non‐hypoxic conditions (PreTreat, A1, A10, A20, MCP). Using these thresholds, a total of 606 genes were identified as differentially upregulated in the CA or the MCPCA treatments (Table S1). We also compared our DEG gene lists with the proposed plant core hypoxia response genes identified by Mustroph et al. (2009) (Table S4).

In the second DEG analysis, we identified which of the 606 hypoxia response genes were differentially regulated based on the ability to perceive ethylene. This consisted of two subsets: the hypoxia upregulated CA only subset, where CA was upregulated compared to all other treatments (PreTreat, A1, A10, A20, MCP, and MCPCA), and the hypoxia upregulated MCPCA only subset, where fruits in the MCPCA treatment were upregulated compared to all other treatments (PreTreat, A1, A10, A20, MCP, and CA). A gene was classified as differentially upregulated if it had > 1 log2 fold change and a *p*adj of < .05. A total of 52 and 20 genes were identified for CA and MCPCA upregulation, respectively.

A third DEG analysis focused on long‐term fruit (CA, MCP, and MCPCA fruit) over the course of the postharvest storage experiment (2, 4, 5, 6, 7, 8, and 9 months postharvest). At each time point, differential expression was performed for CA treatment (hypoxia) versus MCP (normoxia and ethylene perception inhibition) and MCPCA (hypoxia and ethylene perception inhibition), and for MCPCA and CA versus MCP. This resulted in two gene sets we define as “ethylene” and “n‐degron.” The “ethylene” gene set identifies when genes in CA fruit are upregulated compared to MCP and MCPCA fruit. This set indicates which genes responding to hypoxia are also mediated by ethylene sensing and signaling at each time point. The “n‐degron” gene set identifies when genes in MCPCA and CA are upregulated compared to MCP fruit. This set indicates which genes are upregulated in response to hypoxia at each postharvest time point, irrespective of ethylene perception.

Heatmap visualization and clustering of DEG was performed using the package “pretty heatmaps” (pheatmap) version 1.0.12 (Kolde, 2018) in R version 4.1.3 (R Core Team, 2022). Visualization of DEG counts was done using ggplot2 (Wickham, 2009) and dplyr (Wickham et al., 2023) of the tidyverse package (Wickham et al., 2019) as well as ggrepel for intelligent labeling (Slowikowski, 2023). Gene Ontology (GO) functional enrichment analysis was performed on all gene sets using AgriGO v2 (Tian et al., 2017) “Malus x domestica Singular Enrichment Analysis” (Da et al., 2019) (http://bioinformatics.cau.edu.cn/AppleMDO/ ; accessed May 2023). The “GDDH13 V1.1 homology with Arabidopsis reference” was selected (Du et al., 2010; Tian et al., 2017). A dendrogram of the GO hierarchy (biological processes) for the 606 putative hypoxia genes was generated using the “Graphical Result” tool available after the AgriGO v2 GO analysis tool.

### 4.5 Phylogenetic analysis

#### 4.5.1 Identification of orthogroups of interest and construction of phylogenetic trees

To identify orthologous genes between apple and model organisms (e.g., *Arabidopsis thaliana* and *O. sativa*), and to better understand the evolutionary history of gene families of interest, a phylogenetic approach was taken. Our genes of interest come from three pools—the 606 hypoxia responsive apple genes, the 49 core Arabidopsis hypoxia response genes identified by Mustroph et al. (2009), and the 67 putative apple TFs.

First, genes of interest, from both apple and Arabidopsis, were classified into orthogroups (OGs) using PlantTribes2 (Wafula et al., 2023), with the 26Gv2.0 scaffold and the “both BLAST and HMM” option implemented in the GeneFamilyClassifier tool. Orthogroup IDs and genes of interest (GOIs) classified into the corresponding OGs are listed in Tables S1 (606 apple genes), S4 (49 Arabidopsis genes), and S9 (67 TFs). Next, phylogenetic analyses were performed on the OGs of interest following the same method presented in Zhang et al. (2022). Following Zhang et al. (2022), genes from additional Rosaceae genomes classified into OGs of interest were identified and merged with the 26Gv2.0 scaffold—16 additional Rosaceae genomes were used for phylogenetic analysis of the 49 “core hypoxia” Arabidopsis genes, and five were used for the 606 apple hypoxia response genes and 67 TFs. For a detailed description of the selected genomes, please consult the Supplemental Methods (Data S4).

#### 4.5.2 Orthology inference between apple and Arabidopsis

Homologs between apple and Arabidopsis were inferred manually using phylogenetic trees. The bootstrap support value of the node connecting the apple and Arabidopsis genes or a deeper node were recorded as the confidence level of the putative orthology. The method of determining ortholog and confidence level, as well as examples, are detailed in the Supplemental Methods (Data S4).

#### 4.5.3 ERF gene family phylogeny inference

Members of the ERF gene families were classified into multiple orthogroups. To construct a gene family tree containing all the ERFs, the SuperOrthogroup classification from PlantTribes2 was used. First, orthogroups belonging to the same SuperOrthogroup as OG7 (under MCL inflation parameter 3.0 from GeneFamilyClassifier output, Table S12), which contains most of the known Arabidopsis ERF genes, were extracted, resulting in six orthogroups—OG7, OG2171, OG7665, OG14248, OG16955, and OG17668. Two orthogroups were excluded in further analyses due to the following reasons: OG14248 was removed because the PlantTribes2 functional annotations indicate that proteins in this orthogroup are involved in response to dehydration rather than ethylene; OG7665 was removed because *M. domestica* genes in this orthogroup share low sequence similarity to other ERF genes and further InterProScan analyses via the InterPro webserver with default parameters (https://www.ebi.ac.uk/interpro/search/sequence/ accessed: Dec 4th, 2023) indicate that they lack the AP2 domain. For the other four orthogroups, sequences from the seven genomes used for OG1‐10 plus *M. domestica* Golden Delicious v1.0 (Velasco et al., 2010) were integrated with the 26Gv2.0 scaffold. The same method as described above was used for multiple sequence alignment and phylogenetic tree construction with the integrated gene sequences. Phylogenetic trees created by RAxML suffer from low bootstrap issues; therefore, a second tree inference method was deployed using IQ‐TREE version 2.0.3 (Nguyen et al., 2015) with 2000 ultrafast bootstrap replicates (Hoang et al., 2018) and ‐bnni for bootstrap optimization.

#### 4.5.4 Construction of ERF‐VII and PCO sub‐tree

The phylogenetic tree of the two focal gene families, ERF‐VII and PCO, are too large to be visualized easily. To create a simplified phylogenetic tree for those two gene families and highlight orthology between apple and Arabidopsis genes, subtrees were created. The angiosperm‐wide monophyletic ERF‐VII clade was identified from the full gene family tree (Figure S2). Apple (GDDH13) and Arabidopsis genes housed in this clade were extracted. The PCO gene family is relatively smaller; all apple (GDDH13) and Arabidopsis genes from this gene family were extracted. Then, the same methods deployed to construct the full gene family tree were utilized to construct the subtrees. Close examination of PCO and ERF‐VII phylogenies and expression data from our experiment revealed issues with the 3′ UTR annotations for one gene from each family—PCO MD12G1009200 and ERF‐VII MD16G1162800. These genes were dropped from downstream expression analyses. Additional details can be found in the Supplementary Methods (Data S4).

### 4.6 *Cis*‐motif annotation

For *cis*‐motif identification, the 1000‐bp nucleotide genomic sequence of all genes was extracted from the GDDH13 genome file using the provided annotations (Daccord et al., 2017) (accessed using the Genome Database for Rosaceae [GDR; Jung et al., 2019]) and the tool seqkit v2.1.0 (Shen et al., 2016) (Data S1). Annotated 5′‐untranslated regions were included in these 1000‐bp sequences. The presence of the *cis*‐motif HRPE (GCCVCYGGTTTY) (Gasch et al., 2016) was detected in these 1000 nucleotide genomic sequences using the FIMO package (Grant et al., 2011) of Meme‐suite v 5.5.2 (Bailey et al., 2015; https://meme-suite.org/meme/tools/fimo).

### 4.7 GENIE3 transcription factor network construction and analysis

A GENIE3 (Huynh‐Thu et al., 2010) style network was constructed using the sklearn python implementation (Pedregosa et al., 2011). Preprocessing was performed to remove genes that did not have at least 10 samples with at least an expression value of 3 (DESeq2 normalized expression), resulting in a matrix with 143 samples and 23,813 genes. Removal of low expression value genes was performed to reduce runtime and eliminate low expression genes from consideration. Next, a list of apple TFs identified using iTAK (Zheng et al., 2016) was retrieved from the AppleMDO database (http://bioinformatics.cau.edu.cn/AppleMDO/gene_family/; Da et al., 2019). This list consisted of 2965 putative TFs in the GDDH13 apple genome, 1553 of which had detectable levels of gene expression (at least three samples with a count > 10) in our dataset. GENIE3 (Huynh‐Thu et al., 2010) was used to predict the putative gene targets of these 1557 detected TFs in our dataset. Settings for GENIE3 Random Forest were set at *max_features* = sqrt (number of TFs) and *n_estimators* = 1000. The completed regulatory network, with relationships thresholded at five potential TF per gene, is available in Table S13. *r* ^2^ metrics were used to evaluate how well TFs were able to predict the expression values of each gene and can be found in Table S14 and a histogram of the *r* ^2^ values in Figure S8. The number of genes putatively regulated by each of the 1553 TFs genes is graphed as a histogram in Figure S9.

After the genome‐wide TF regulatory network construction, the 606 genes identified as differentially expressed in hypoxic conditions and their putative TFs were selected from the network to form subgraphs and visualized using Cytoscape v3.9.1 (Shannon et al., 2003). Thresholding of the network was performed by selecting the top 5 TFs based on importance value (Breiman, 2001) for each gene target in the 606 hypoxia gene set. This resulting subgraph was then reduced by excluding TFs which did not regulate at least 10 of the hypoxia genes. Removing TFs predicted to regulate fewer than 10 hypoxia genes was an ad hoc threshold intended to remove TFs with minimal relationship to the hypoxia pathway, allowing us to concentrate on those more likely involved in hypoxia regulation. The network analysis feature within Cytoscape was used to generate node degree. Node degree is a measure of how many genes a TF is predicted to regulate (the number of edges attached to that TF in the network). This is visualized as the size of each TF in the network images. TFs were classified as targeting “ethylene‐mediated hypoxia” genes if the majority of the genes they targeted belonged to either the hypoxia CA upregulated only (52 DE genes) or the hypoxia MCPCA upregulated only (20 DE genes) subsets; otherwise, they were classified as targeting “standard hypoxia” genes if the majority of the genes they targeted were in the remaining portion 606 hypoxia gene set (534 genes). For this classification, the “majority” of targeted genes was normalized based on the maximum number of genes targeted by a TF in either category to account for the different number of genes in each category, with the majority for “ethylene‐mediated hypoxia” being defined by TF falling above the line *y* = 24/143 * *x*, whereas TFs targeting “standard hypoxia” genes were classified as anything below this line (Figure 6). Finally, a subgraph of the above regulatory network was also created that included only the ERF VII genes and their putative targets. An ERF VII gene was classified as a putative regulator of a target if it was in the top 5 TF regulating that target.

## CONFLICT OF INTEREST STATEMENT

To the best of our knowledge, the authors have no conflict of interest, financial or otherwise.

## DATA AVAILABILITY

Apple GDDH13 PCO and ERF VII genes referred to in this paper are available as Data S5. RNA‐Seq data used in the paper is available on NCBI as BioProject PRJNA938164.

## PEER REVIEW

The peer review history for this article is available in the Supporting Information for this article.

## ACCESSION NUMBERS

None.

## Supporting information


**Data S1:** Genomic sequences of the 10 putative apple PCO homologs, and the five ERF‐VII homologs in the GDDH13 apple genome.

**Data S2:** Homologs of all 606 hypoxia response genes identified in this study across 42 representative species of land plants).

**Data S3:** Sequence alignments of MD16G1162800 and MD16G1162900 with all other Malus and Pyrus homologs.

**Data S4:** Supporting Information.

**Data S5:** Putative apple GDDH13 PCO and ERFV VII coding sequences.

**Data S6.** Peer Review.

**Figure S1:** Heatmap of gene expression of the Hypoxia CA and Hypoxia MCPCA DE subsets.

**Figure S2:** GO Term hierarchical Pclustering ‐ 606 putative hypoxia response genes.

**Figure S3:** Heatmap of gene expression of the apple homologs from the Mustroph et al. (2009) core Arabidopsis hypoxia response genes.

**Figure S4:** Heatmap of differentially expressed ‘n‐degron’ genes among fruit in long term storage.

**Figure S5:** Heatmap of differentially expressed ‘ethylene’ genes among fruit in long term storage.

**Figure S6:** Putative Plant Cysteine Oxidase (PCO) Gene phylogeny.

**Figure S7:** Putative group VII ethylene response factor (ERF VII) Transcription Factor family gene phylogeny.

**Figure S8:** Histogram distribution of r^2^ of transcription factor predictability in the full apple transcription factor network.

**Figure S9:** Histogram distribution of the size of the regulatory network of apple transcription factors.

**Figure S10:** MD16G1162800 and MD16G1162900 *Malus* and *Pyrus* gene phylogeny.

**Table S1:** Differential Expression Results ‐ upregulated genes in hypoxic conditions.

**Table S2:** Differential Expression Results ‐ upregulated genes in CA only or MCPCA only.

**Table S3:** GO Enrichment Results ‐ 606 significantly upregulated genes in hypoxic conditions.

**Table S4:** Comparison Mustroph et al. (2009) core hypoxia genes (*Arabidopsis)* with *Malus domestica* hypoxia response.

**Table S5:** Differential Expression Results ‐ upregulated genes in hypoxic conditions, n‐degron.

**Table S6:** Differential Expression Results ‐ upregulated genes in hypoxic conditions, ethylene.

**Table S7:** GO Enrichment Results ‐ n‐degron and ethylene hypoxia genes.

**Table S8:** Putative Apple Hypoxia Transcription Factor regulatory network.

**Table S9:** Hypoxia CA and Hypoxia MCPCA Transcription Factors.

**Table S10:** Putative Apple ERFV VII TF regulatory network.

**Table S11:** GO Enrichment Results ‐ putative targets for each Apple ERFV VII TF.

**Table S12:** GDDH13 ERF SuperOrthogroup classifications.

**Table S13:** Full Apple transcription factor regulatory network.

**Table S14:** Support metrics for each gene in the hypoxia transcription factor regulatory network.
