# Supplementary material for: Transcriptomics of long‐term, low oxygen storage coupled with ethylene signaling interference suggests neofunctionalization of hypoxia response pathways in apple ( Malus domestica )
Source: Plant Direct. 2024 Dec 20;8(12):e70025. doi: 10.1002/pld3.70025 (PMC11660084; doi:10.1002/pld3.70025)
Supplement: Supplementary file 4 — Data S4: Supporting Information. [file PLD3-8-e70025-s003.docx]

Supplemental Methods – Phylogenetic Analysis

*Phylogenetic Analysis*

Genes of interest, from both apple and Arabidopsis, were classified into orthogroups (OGs) using PlantTribes2 (Wafula et al., 2022), with the 26Gv2.0 scaffold and the ‘*both BLAST and HMM’* option implemented in the GeneFamilyClassifier tool. Orthogroup IDs and GOIs classified into the corresponding OGs are listed in Tables S1 (606 apple genes), S4 (49 Arabidopsis genes), and S9 (67 transcription factors). Next, phylogenetic analyses were performed on the OGs of interest following the same method presented in Zhang et al. (2022). For the OGs identified based on the 49 Arabidopsis genes, all genes classified into the same orthogroup were identified from 16 Rosaceae genomes [15 from [(Zhang et al., 2022)](https://paperpile.com/c/TpvKtl/QImDS) plus *Malus baccata* [(Chen et al., 2019)](https://paperpile.com/c/TpvKtl/01tfA) which was classified using the same method as the first 15 genomes] and were merged with sequences from the 26Gv2.0 scaffolding species following methods from [(Zhang et al., 2022)](https://paperpile.com/c/TpvKtl/QImDS). For the OGs identified based on the 606 apple genes and the 67 transcription factors, only five representative Rosaceae genomes [*Malus domestica* GDDH13 and ‘Honeycrisp’ [(Khan et al., 2022)](https://paperpile.com/c/TpvKtl/YT5d); *Pyrus betulifolia* [*(Dong et al., 2020)*](https://paperpile.com/c/TpvKtl/0kXn); *Fragaria vesca* v4.0a2 [(Shulaev et al., 2011)](https://paperpile.com/c/TpvKtl/3IQv); Rosa chinensis Old Blush v2.0 (Raymond et al., 2018)] were selected, thus a total of 31genomes, for the following reasons: 1) The 606 hypoxia GOIs belong to 442 OGs, performing phylogenetic analysis on all those OGs with 42 species will take an excessive amount of resource; 2) Some OGs from this list contains over 10,000 sequences from all the 42 species, infeasible for phylogenetic analysis. These resulting files (which contain homologs of all three sets of our GOIs across representative species of land plants) are available in Data S2 and were used as input for gene family alignment and phylogeny. Some of the GOIs belong to large orthogroups (e.g. OG1 contains 8,275 sequences from the 31 sampled genomes) and the number of sequences in these orthogroups exceeded the input sequence limit of the alignment software used by PlantTribes2, MAFFT, and available computational resources. To maximize the number of taxonomically diverse genomes included in the phylogeny while not exceeding the aforementioned limits, a subset of seven genomes were used for OG1-OG12 [*Malus domestica* GDDH13 and ‘Honeycrisp’ [(Khan et al., 2022)](https://paperpile.com/c/TpvKtl/YT5d); *Pyrus betulifolia* [*(Dong et al., 2020)*](https://paperpile.com/c/TpvKtl/0kXn); *Fragaria vesca* v4.0a2 [(Shulaev et al., 2011)](https://paperpile.com/c/TpvKtl/3IQv); *Arabidopsis thaliana* TAIR10 [(Lamesch et al., 2012)](https://paperpile.com/c/TpvKtl/7oNn); *Vitis vinifera* v2.1 [(Jaillon et al., 2007)](https://paperpile.com/c/TpvKtl/ziil); *Oryza sativa* v7.0 [(Ouyang et al., 2007)](https://paperpile.com/c/TpvKtl/4WIy)], and a subset of 13 genomes were used for OG13-34 [the seven mentioned above plus *Rosa chinensis* v2 [(Raymond et al., 2018)](https://paperpile.com/c/TpvKtl/acnD); *Populus trichocarpa* v3.0 [(Tuskan et al., 2006)](https://paperpile.com/c/TpvKtl/FDcC); *Theobroma cacao* v1.1 [(Motamayor et al., 2013)](https://paperpile.com/c/TpvKtl/iWLB); *Solanum lycopersicum* v2.4 [(Tomato Genome Consortium, 2012)](https://paperpile.com/c/TpvKtl/7DmW); *Nelumbo nucifera* v1.0 [(Ming et al., 2013)](https://paperpile.com/c/TpvKtl/zzqL); *Amborella trichopoda* v1.0 [(Amborella Genome Project, 2013)](https://paperpile.com/c/TpvKtl/kZ7C)]. Orthogroup multiple sequence alignment, phylogenetic tree estimation, homology inference, and gene model evaluation were performed following methods from [(Zhang et al., 2022)](https://paperpile.com/c/TpvKtl/QImDS). Phylogenetic trees were visualized using Dendroscope (version 3.8.8) [(Huson & Scornavacca, 2012)](https://paperpile.com/c/TpvKtl/9GC9q).

*Ortholog inference between apple and Arabidopsis*

First, the smallest monophyletic clade (i.e. the target clade) containing the apple and Arabidopsis genes of interest were determined and the node connecting the target clade is denoted as node 1. If all the sequences in the target clade are from expected taxa (i.e. the majority of the sampled rosids except *Vitas and Eucalyptus*) and node 1 has a strong bootstrap support (i.e. >=50), the bootstrap value of node 1 is recorded (see example 1). If node 1 does not have a strong bootstrap support and/or the target clade contains unexpected taxa sequences (e.g. non-rosid sequences), deeper nodes and broader clades were examined (see example 2). The first broader (rosid-wide, core eudicot-wide, eudicot-wide, angiosperm-wide) clade with expected taxa supported by a node with bootstrap value >=50 were denoted as node 2. If none of the broader clade has >=50 bootstrap support, then the node with the highest support were denoted as node 2. The bootstrap support of node 2 was recorded with a note indicating the breadth of the clade sampling. If bootstrap support values for all the possible node 2 are lower (or similar) than node 1 or node 2 does not exist (see example 3), then the bootstrap value of node 1 is recorded. For the genes of interest lacking putative orthologs (see example 4), homologs (putative orthologs or paralogs) were identified if possible. The examples below are from phylogenetic trees constructed to identify apple ortholog of the 49 core Arabidopsis hypoxia genes, the same principle was used to infer Arabidopsis ortholog of the 606 apple hypoxia response genes.


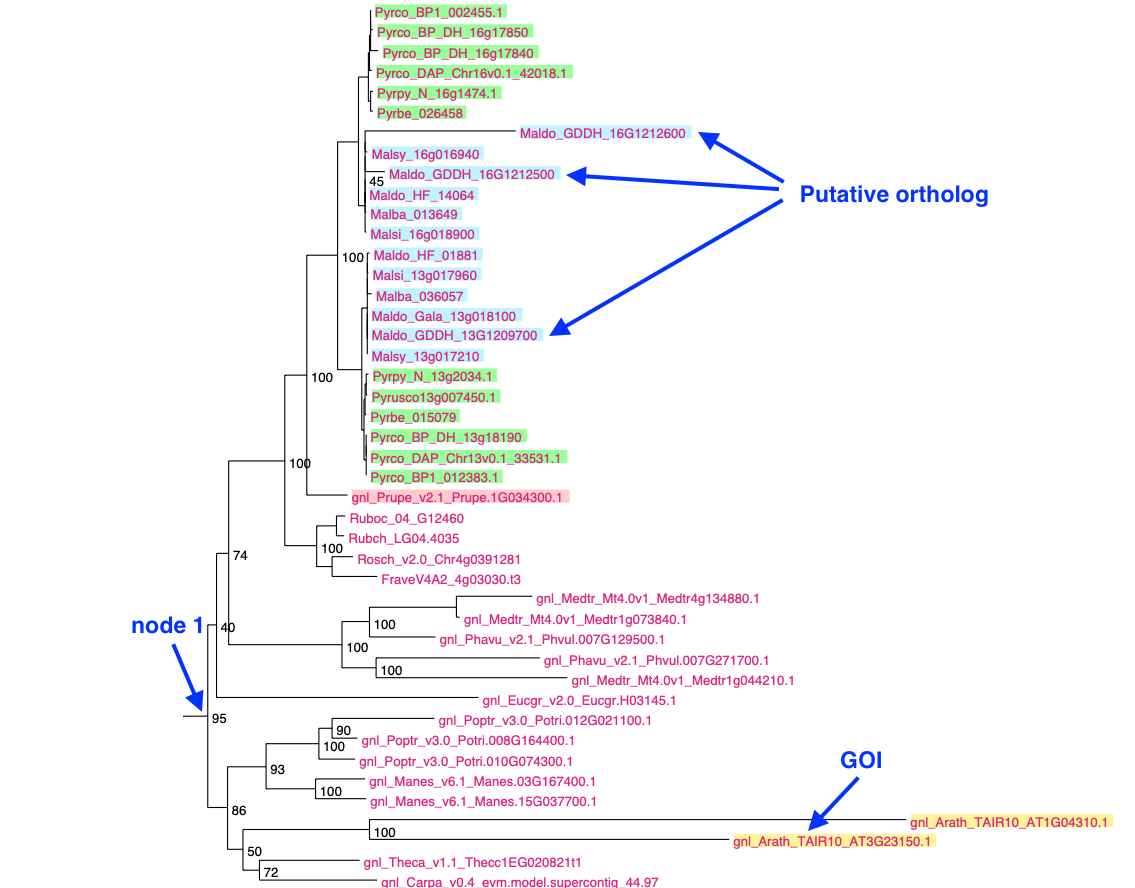


Example 1 (OG1711): The Arabidopsis gene of interest and three putative apple orthologs are housed in a monophyletic clade with sequences from only expected taxa and a strong bootstrap support (bs) value of 95 (node 1). Bs of node 1 (bs=95) was recorded.


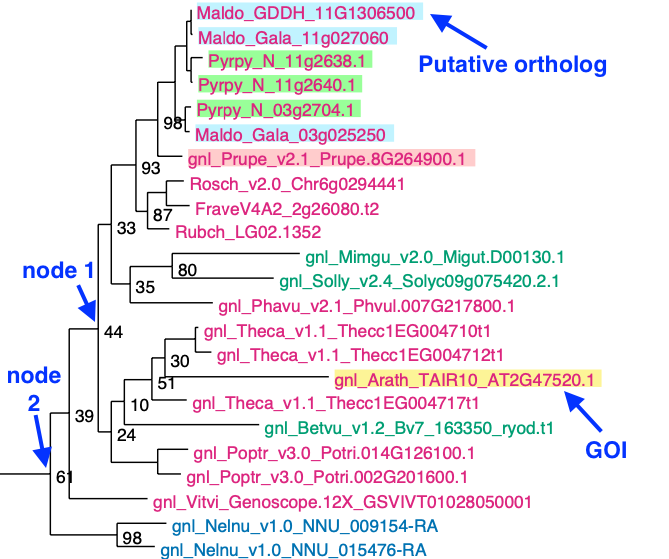


Example 2 (OG7): The GOI and its putative ortholog are grouped in a clade with a bootstrap support of 44 (node 1). There are sequences from unexpected taxa, i.e. asterid sequences (colored in green) within a rosid clade. A deeper node (node 2 with bootstrap support of 61), which identifies a eudicot-wide clade was selected as supporting evidence of the inferred orthology. Bs of node 2 (bs=61) was recorded and a note was added indicating that the recorded bs is bs of a eudicot-wide clade housing the apple and Arabidopsis genes.


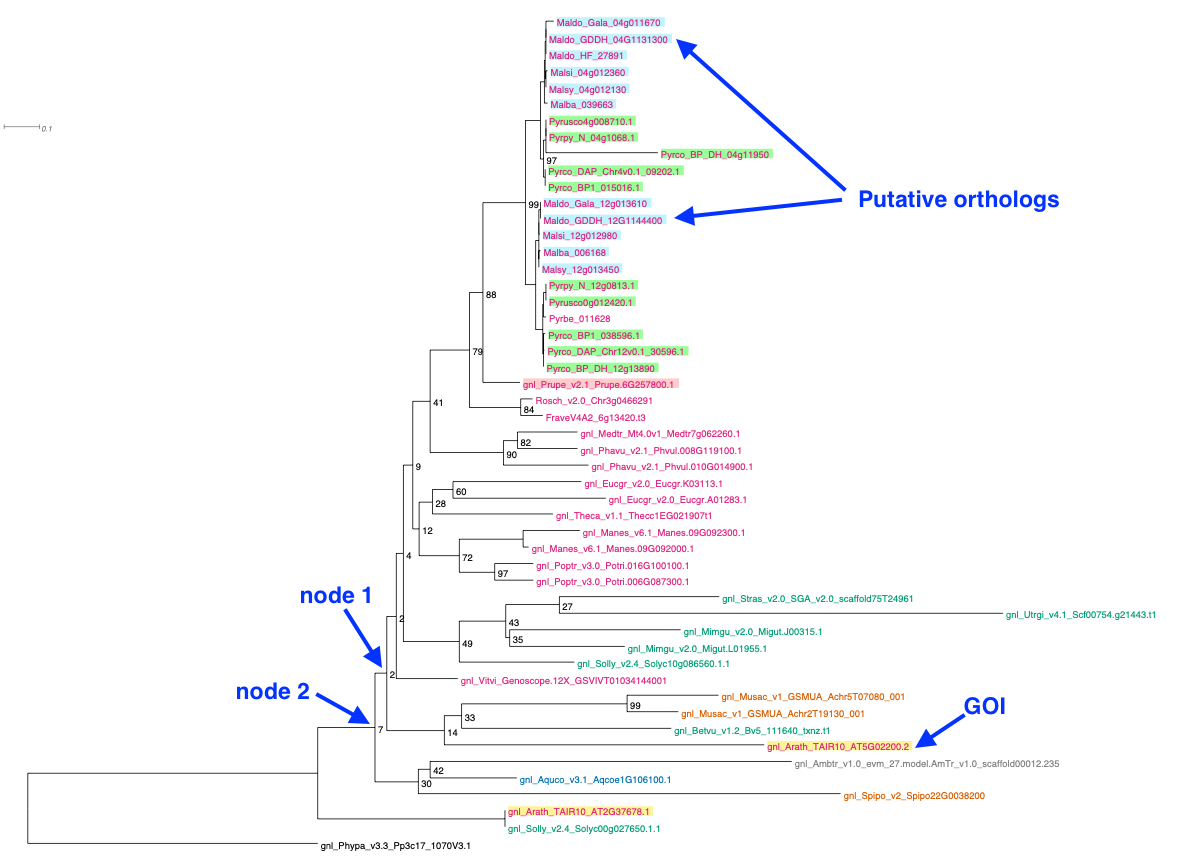


Example 3 (OG8162): The figure below showed the complete tree of OG8162. Although the clade grouping the apple and Arabidopsis genes is weakly supported (bs of node 1 = 2, bs of node 2 =7), a single origin of the ancestral gene can be inferred, thus supporting the orthology between the apple and Arabidopsis genes. Bs of node 1 (bs=2), was recorded.


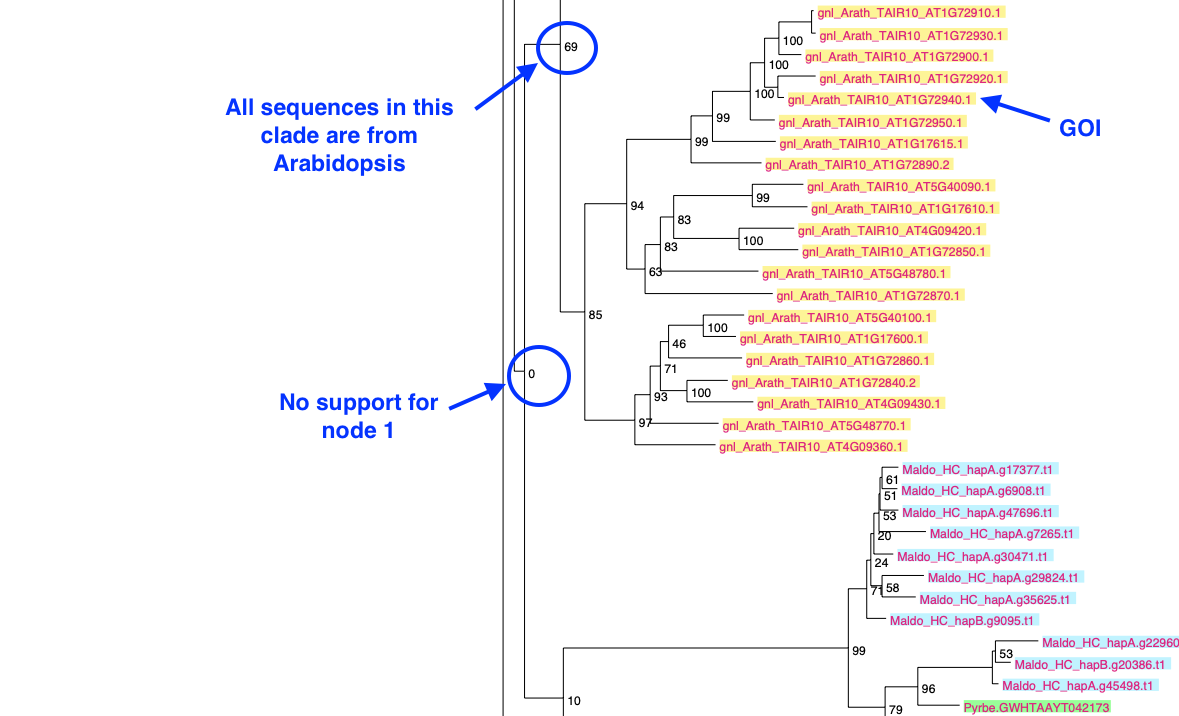


Example 4(OG4): This tree shows rapid evolution in Arabidopsis. The tree has very low to no support for its topology, thus infeasible to infer orthology.

*Construction of ERF-VII and PCO sub-tree*

Upon completion of the PCO and ERF-VII phylogenies (Figure S6 and S7 respectively), Malus and Arabidopsis GOIs were extracted to construct smaller gene phylogeny subsets (Figure 3A and Figure 4A respectively). Upon closer examination of the phylogenies and the corresponding expression patterns for our genes of interest, two genes in the PCO phylogeny (MD12G1009100 and MD12G1009200) and two genes in the ERF-VII phylogeny (MD16G1162800 and MD16G1162900) needed additional scrutiny. The nucleotide sequences of these two gene pairs were highly similar to each other. The two PCO genes on the other hand had disparate expression patterns, where one gene (MD12G1009100) had many read counts and the other (MD12G1009200) had no reads assigned. The two ERF-VII genes shared very similar expression patterns across all samples but had different read counts (10’s vs 100’s respectively). This can be due to 1) gene fragmentation caused by assembly or annotation errors, where two genes appear in the assembly but are really fragments of the same gene (an example can be found in Figure 8 from Wafula et al., 2022), or 2) there are actually two full lengths genes annotated from the assembly but both genes are present for one of two reasons, a very recent duplication event, or the same genomic fragment was assembled twice in the genome. To unveil the nature of these genes, we compared them to their homologs in other Rosaceae species using the phylogenetic trees constructed in the precious step. OG442 (PCO) is a relatively small orthogroups, therefore the tree (Figure S6) was constructed with sequences from the 42 plant species listed in the main methods section (See Phylogenetic Analysis above). OG7 (ERF), however, is one of the gene families constructed with a subset of the 42 species. To gain a more comprehensive view of the GDDH13 orthologs in OG7, we created sequence alignment of OG7 using the full collection of sequences from the 42 species with MAFFT, and constructed phylogenetic tree using the FastTree algorithm, also implemented in PlantTribes2. FastTree was used to construct the phylogenetic tree of OG7 due to the large number of sequences to be analyzed from the 42 genomes (Figure S10). The topology and sequences in the ERF-VII clade from the FastTree derived phylogeny was compared to that from the RAxML derived sub-sampled phylogeny to ensure robust support of the ERF-VII clade in the FastTree phylogeny. Upon examining the sequence alignments and the phylogenies (Figure S7 and S10) more closely, we observed that the GDDH13 genome is the only genome (among all apple and pear genomes available and used in the phylogenies) that has two copies of these genes. All other *Malus* and *Pyru*s genomes, including the ‘Gala’ genome, have only one copy (Data S3). Close examination of the two PCOs reveal that MD12G1009200 has no annotated 3’ UTR in the GDDH13 genome, which is likely why no reads were assigned to that gene in our experiment. Thus, MD12G1009200 was excluded from downstream expression analyses. Close examination of the two ERF-VII genes shows both copies in the GDDH13 genome and their homologs have similar gene lengths. Therefore, this is unlikely a case of erroneous gene fragmentation. Closer examination of the sequence alignments for the two ERF-VII GDDH13 gene sequences, the ‘Gala’ genome gene sequence had 100% alignment agreement between it and MD16G1162800, and 3 SNPs between it and MD16G1162900. The three SNPs in MD16G1162900 produce a slightly different protein sequence. MD16G1162800 and the ‘Gala’ gene sequence had higher alignment agreement to the ‘Honeycrisp’ and ‘Hanfu’ sequences than to MD16G1162900. However, when examining read pileups for both MD16G1162800 and MD16G1162900, we found that MD16G1162800 had a shorter 3’ UTR region compared to MD16G1162900, which may be the reason why more reads were assigned to MD16G1162900. A conclusive resolution of which gene is the ‘true’ gene is outside the scope of this paper. We proceeded to use only MD16G1162900 for downstream expression analyses, as it was the sequence with a complete 3’ UTR region, and we used 3’Quant Seq for generating our transcript data.

**Supplemental Figure Captions**

**Figure S1**: Heatmap of the hypoxia MCPCA and CA DE subset.

**Figure S2:** GO Enrichment hierarchical Pclustering for the 606 DEG genes. Downloaded from AgriGO v2.0, generated using biological processes

**Figure S3:**  Heat map of the 59 apple homologs of the 49 core hypoxia response genes identified in Arabidopsis by Mustroph et al. (2009) (expression value over 3 for at least 10 samples using the DESeq2 normalized dataset). The plot is split based on if the gene was shown to be differentially expressed during hypoxic conditions in the apple dataset (DEG Hypoxia - top 27 rows) or if it was not (Not DEG Hypoxia - bottom 32 rows).

**Figure S4:** Heatmap of differentially expressed genes in long term stored fruit, upregulated in at least one time point in the MCPCA and CA treatments compared to the MCP treatment. Genes are defined here as ‘n-degron’ DEGs. Rows (genes) were clustered based on expression pattern similarity. The x-axis annotations above the heatmap indicate samples grouped by treatment and ordered by days postharvest (the earliest time point sample is first within each treatment). Each cell represents the average expression of 3 biological replicates.

**Figure S5:** Heatmap of differentially expressed genes in long term stored fruit, upregulated in at least one time point in the CA treatments compared to the MCP and MCPCA treatments. Genes are defined here as ‘ethylene’ DEGs. Rows (genes) were clustered based on expression pattern similarity. The x-axis annotations above the heatmap indicate samples grouped by treatment and ordered by days postharvest (the earliest timepoint sample is first within each treatment). Each cell represents the average expression of 3 biological replicates.

**Figure S6:** Putative Plant Cysteine Oxidase (PCO) gene phylogeny, generated using PlantTribes2 (Wafula et al., 2021).

**Figure S7:** Putative Ethylene Response Factor group VII (ERFVII) Transcription Factor family phylogeny, generated using PlantTribes2 (Wafula et al., 2021).

**Figure S8:** *r^2^* histogram distribution of the 23,813 genes in the putative GDDH13 regulatory network based on our sample set. Pre-processing was performed to remove genes that did not have at least 10 samples with an expression value at least of 3 (Deseq2 normalized expression), resulting in 143 samples and 23,813 genes being considered as part of our regulatory network. Removal of lowly expressed genes was done to reduce runtime and eliminate low expression genes from consideration.

**Figure S9:** Histogram of the number of genes each of the 1,553 identified transcription factor is predicted to regulate in the full regulatory network (all GDDH13 genes - Table S13), thresholded at 5 potential transcription factors per targeted gene. The x-axis has bins of TFs arranged according to the number of genes regulated.

**Figure S10:** MD16G1162800 and MD16G1162900 phylogeny with all other Malus and Pyrus homologs included. Only in the GDDH13 genome do two copies of this gene appear.

**References**

Amborella Genome Project. (2013). The Amborella genome and the evolution of flowering plants. *Science*, 342(6165), 1241089.

Berardini, T. Z., Reiser, L., Li, D., Mezheritsky, Y., Muller, R., Strait, E., & Huala, E. (2015). The Arabidopsis information resource: Making and mining the “gold standard” annotated reference plant genome. Genesis, 53(8), 474–485.

Berardini, T. Z., Reiser, L., Li, D., Mezheritsky, Y., Muller, R., Strait, E., & Huala, E. (2015). The Arabidopsis information resource: Making and mining the “gold standard” annotated reference plant genome. Genesis, 53(8), 474–485.

Dolgikh, V. A., Pukhovaya, E. M., & Zemlyanskaya, E. V. (2019). Shaping ethylene response: The role of EIN3/EIL1 transcription factors. Frontiers in Plant Science, 10, 1030.

Dong, X., Wang, Z., Tian, L., Zhang, Y., Qi, D., Huo, H., Xu, J., Li, Z., Liao, R., Shi, M., Wahocho, S. A., Liu, C., Zhang, S., Tian, Z., & Cao, Y. (2020). De novo assembly of a wild pear (Pyrus betuleafolia) genome. Plant Biotechnology Journal, 18(2), 581–595.

Huson, D. H., & Scornavacca, C. (2012). Dendroscope 3: An interactive tool for rooted phylogenetic trees and networks. Systematic Biology, 61(6), 1061–1067.

Jaillon, O., Aury, J.‐M., Noel, B., Policriti, A., Clepet, C., Casagrande, A., Choisne, N., Aubourg, S., Vitulo, N., Jubin, C., Vezzi, A., Legeai, F., Hugueney, P., Dasilva, C., Horner, D., Mica, E., Jublot, D., Poulain, J., Bruyère, C., … Wincker, P. (2007). The grapevine genome sequence suggests ancestral hexaploidization in major angiosperm phyla. Nature, 449(7161), 463–467.

Khan, A., Carey, S. B., Serrano, A., Zhang, H., Hargarten, H., Hale, H., Harkess, A., & Honaas, L. (2022). A phased, chromosome‐scale genome of “Honeycrisp” apple (Malus domestica). GigaByte, 2022, gigabyte69. <https://doi.org/10.46471/gigabyte.69>

Lamesch, P., Berardini, T. Z., Li, D., Swarbreck, D., Wilks, C., Sasidharan, R., Muller, R., Dreher, K., Alexander, D. L., Garcia‐Hernandez, M., Karthikeyan, A. S., Lee, C. H., Nelson, W. D., Ploetz, L., Singh, S., Wensel, A., & Huala, E. (2012). The Arabidopsis Information Resource (TAIR): Improved gene annotation and new tools. Nucleic Acids Research, 40, D1202–D1210.

Ming, R., VanBuren, R., Liu, Y., Yang, M., Han, Y., Li, L.‐T., Zhang, Q., Kim, M.‐J., Schatz, M. C., Campbell, M., Li, J., Bowers, J. E., Tang, H., Lyons, E., Ferguson, A. A., Narzisi, G., Nelson, D. R., Blaby‐Haas, C. E., Gschwend, A. R., … Shen‐Miller, J. (2013). Genome of the long‐living sacred lotus (Nelumbo nucifera Gaertn.). Genome Biology, 14(5), R41.

Motamayor, J. C., Mockaitis, K., Schmutz, J., Haiminen, N., Livingstone, D. 3rd, Cornejo, O., Findley, S. D., Zheng, P., Utro, F., Royaert, S., Saski, C., Jenkins, J., Podicheti, R., Zhao, M., Scheffler, B. E., Stack, J. C., Feltus, F. A., Mustiga, G. M., Amores, F., & Kuhn, D. N. (2013). The genome sequence of the most widely cultivated cacao type and its use to identify candidate genes regulating pod color. Genome Biology, 14(6), r53.

Ouyang, S., Zhu, W., Hamilton, J., Lin, H., Campbell, M., Childs, K., Thibaud‐Nissen, F., Malek, R. L., Lee, Y., Zheng, L., Orvis, J., Haas, B., Wortman, J., & Buell, C. R. (2007). The TIGR Rice genome annotation resource: Improvements and new features. Nucleic Acids Research, 35, D883–D887.

Raymond, O., Gouzy, J., Just, J., Badouin, H., Verdenaud, M., Lemainque, A., Vergne, P., Moja, S., Choisne, N., Pont, C., Carrère, S., Caissard, J.‐C., Couloux, A., Cottret, L., Aury, J.‐M., Szécsi, J., Latrasse, D., Madoui, M.‐A., François, L., … Bendahmane, M. (2018). The Rosa genome provides new insights into the domestication of modern roses. Nature Genetics, 50(6), 772–777.

Shulaev, V., Sargent, D. J., Crowhurst, R. N., Mockler, T. C., Folkerts, O., Delcher, A. L., Jaiswal, P., Mockaitis, K., Liston, A., Mane, S. P., Burns, P., Davis, T. M., Slovin, J. P., Bassil, N., Hellens, R. P., Evans, C., Harkins, T., Kodira, C., Desany, B., … Folta, K. M. (2011). The genome of woodland strawberry (Fragaria vesca). Nature Genetics, 43(2), 109–116.

Tomato Genome Consortium. (2012). The tomato genome sequence provides insights into fleshy fruit evolution. Nature, 485(7400), 635–641.

Tuskan, G. A., Difazio, S., Jansson, S., Bohlmann, J., Grigoriev, I., Hellsten, U., Putnam, N., Ralph, S., Rombauts, S., Salamov, A., Schein, J., Sterck, L., Aerts, A., Bhalerao, R. R., Bhalerao, R. P., Blaudez, D., Boerjan, W., Brun, A., Brunner, A., … Rokhsar, D. (2006). The genome of black cottonwood, Populus trichocarpa (Torr. & Gray). Science, 313(5793), 1596–1604.
