# Supplementary figures and images for: Transcriptomics of long‐term, low oxygen storage coupled with ethylene signaling interference suggests neofunctionalization of hypoxia response pathways in apple ( Malus domestica )
Source: Plant Direct. 2024 Dec 20;8(12):e70025. doi: 10.1002/pld3.70025 (PMC11660084; doi:10.1002/pld3.70025)

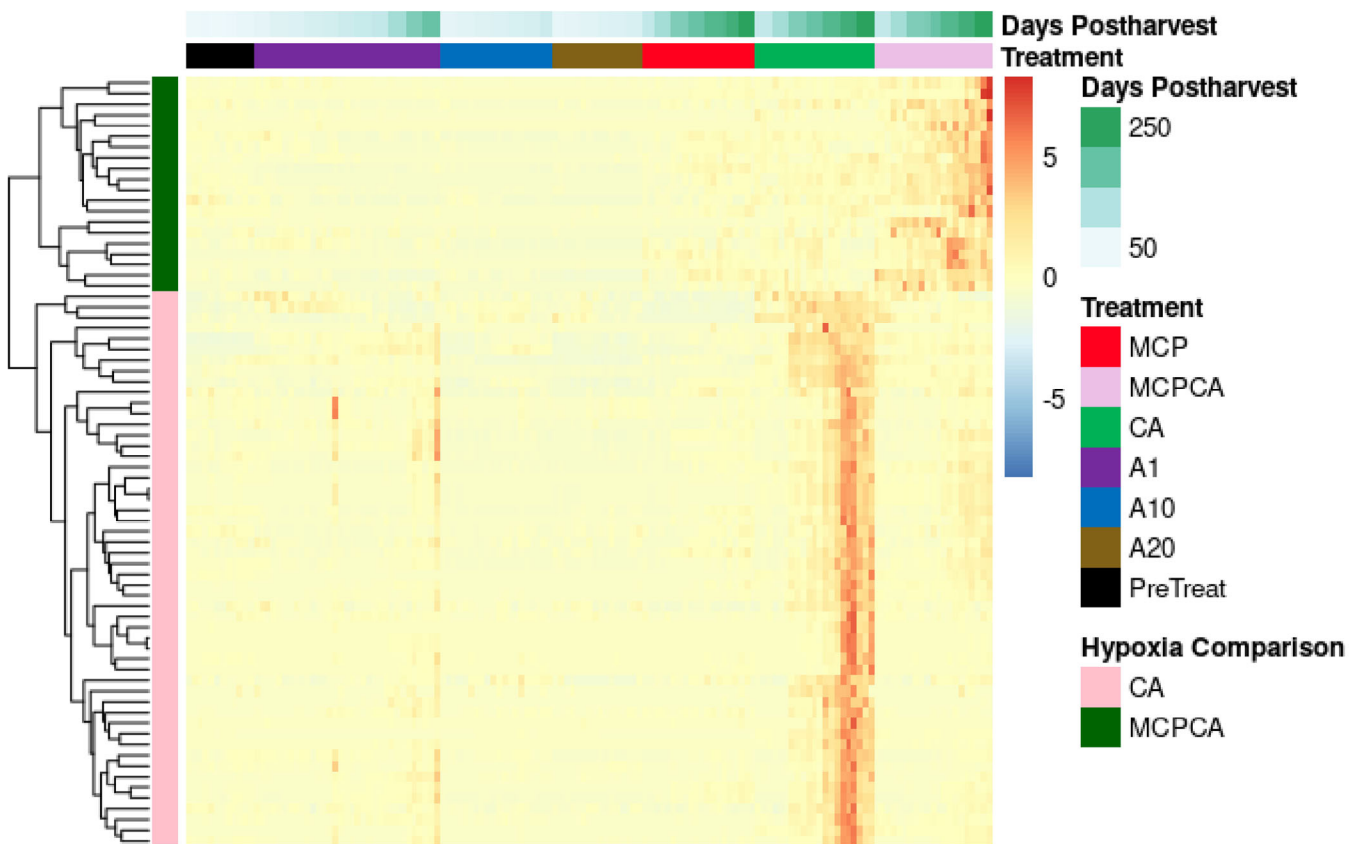

Supplement: Supplementary file 7 — Figure S1: Heatmap of gene expression of the Hypoxia CA and Hypoxia MCPCA DE subsets. [file PLD3-8-e70025-s030.pdf]

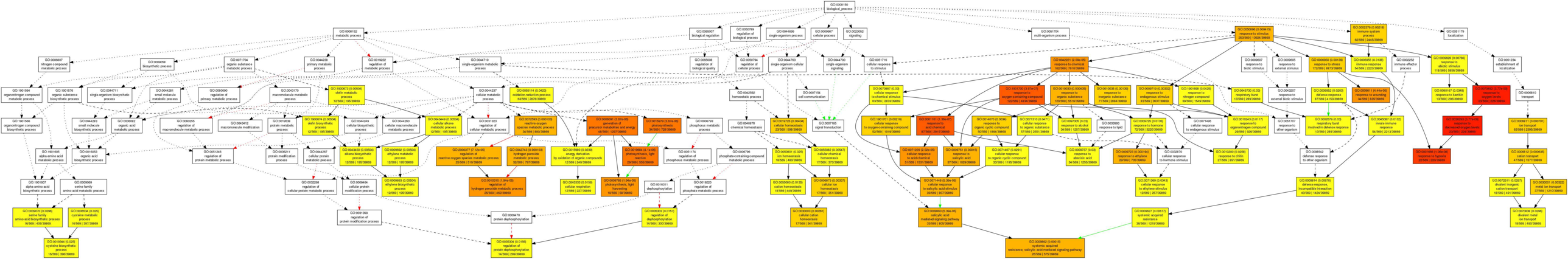

Supplement: Supplementary file 8 — Figure S2: GO Term hierarchical Pclustering ‐ 606 putative hypoxia response genes. [file PLD3-8-e70025-s010.pdf]

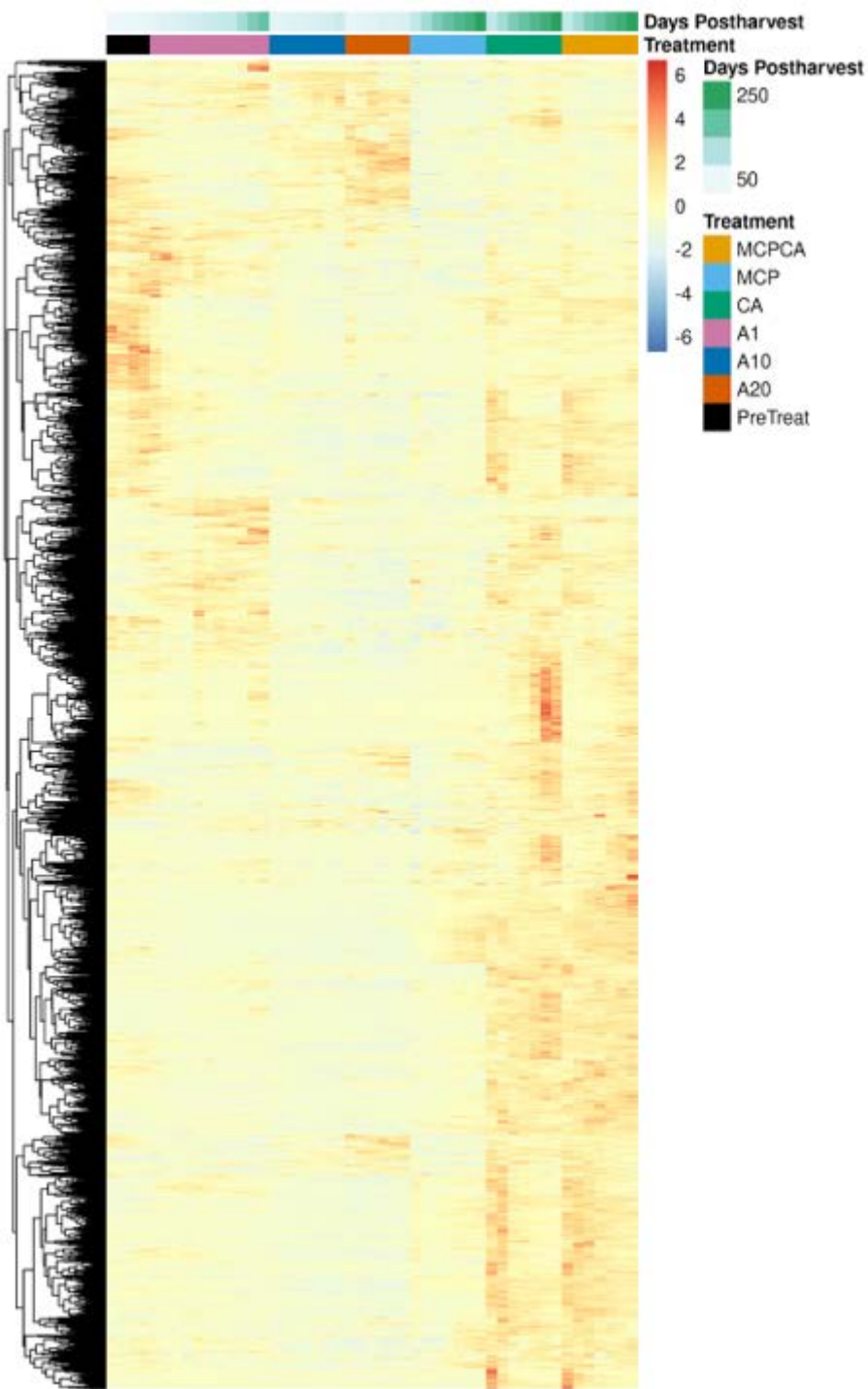

Supplement: Supplementary file 10 — Figure S4: Heatmap of differentially expressed ‘n‐degron’ genes among fruit in long term storage. [file PLD3-8-e70025-s020.pdf]

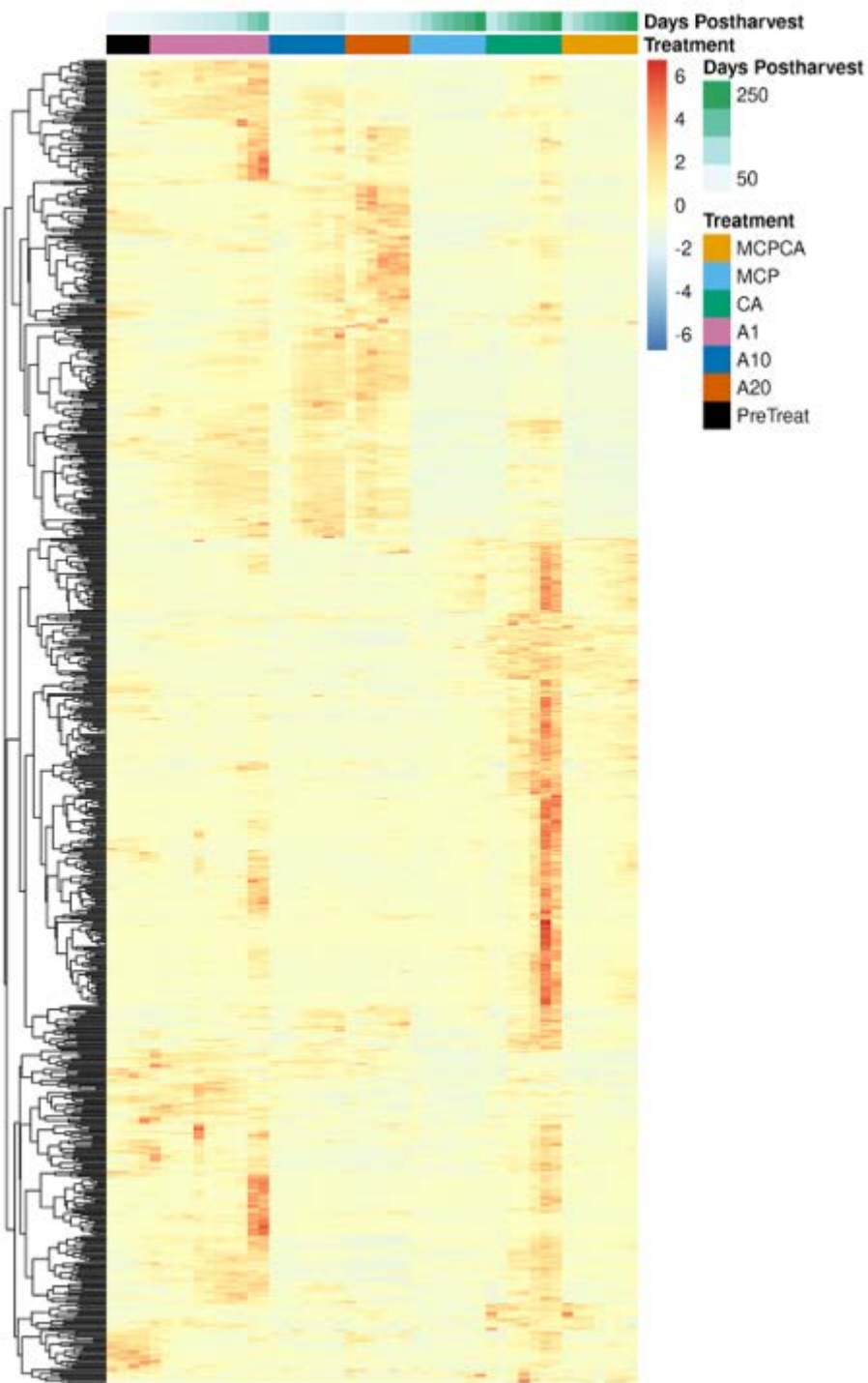

Supplement: Supplementary file 11 — Figure S5: Heatmap of differentially expressed ‘ethylene’ genes among fruit in long term storage. [file PLD3-8-e70025-s016.pdf]

Gene Count

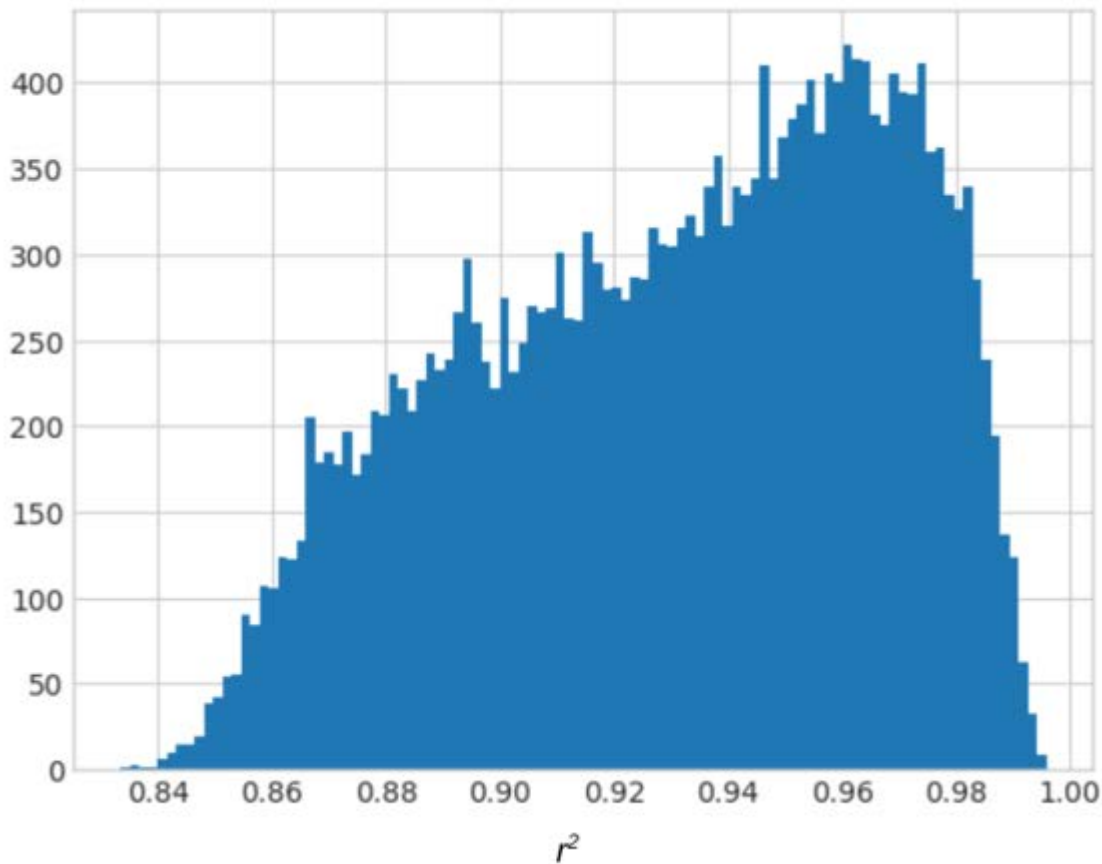

Supplement: Supplementary file 14 — Figure S8: Histogram distribution of r2 of transcription factor predictability in the full apple transcription factor network. [file PLD3-8-e70025-s006.pdf]

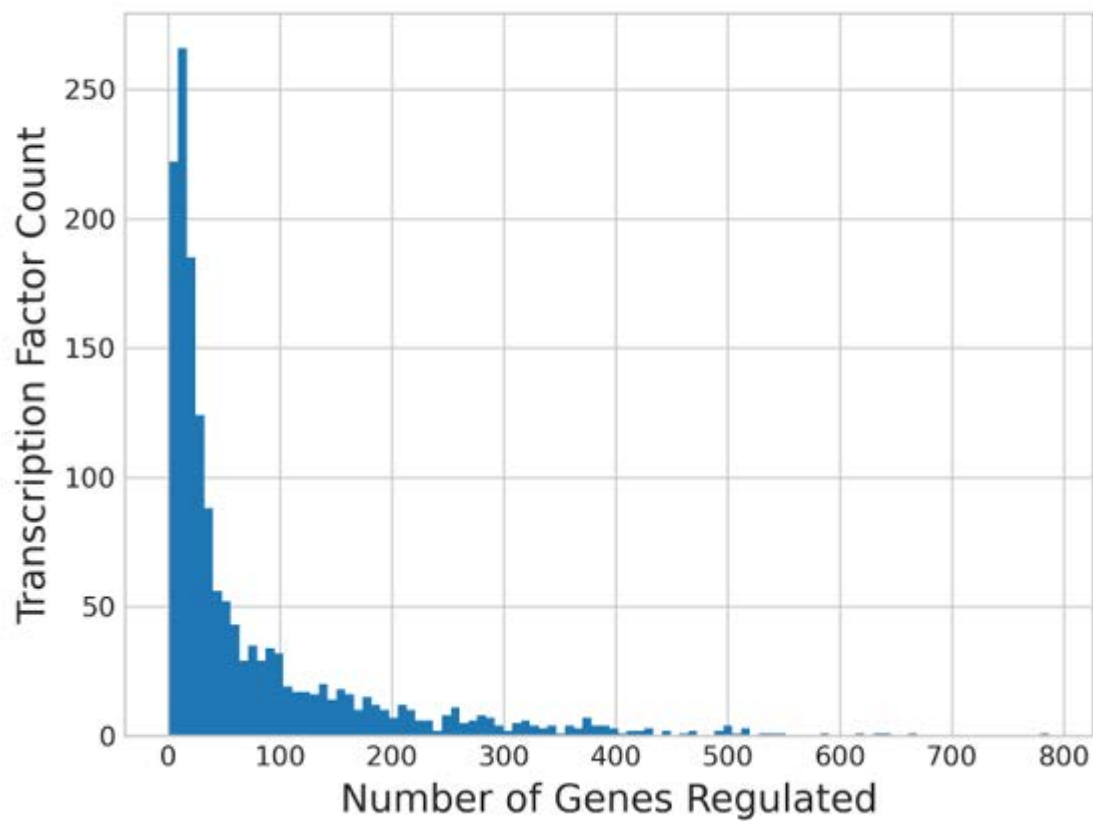

Supplement: Supplementary file 15 — Figure S9: Histogram distribution of the size of the regulatory network of apple transcription factors. [file PLD3-8-e70025-s026.pdf]
