# Supplementary material for: Transcriptomics of long‐term, low oxygen storage coupled with ethylene signaling interference suggests neofunctionalization of hypoxia response pathways in apple ( Malus domestica )
Source: Plant Direct. 2024 Dec 20;8(12):e70025. doi: 10.1002/pld3.70025 (PMC11660084; doi:10.1002/pld3.70025)
Supplement: Supplementary file 9 — Figure S3: Heatmap of gene expression of the apple homologs from the Mustroph et al. (2009) core Arabidopsis hypoxia response genes. [file PLD3-8-e70025-s002.pdf]

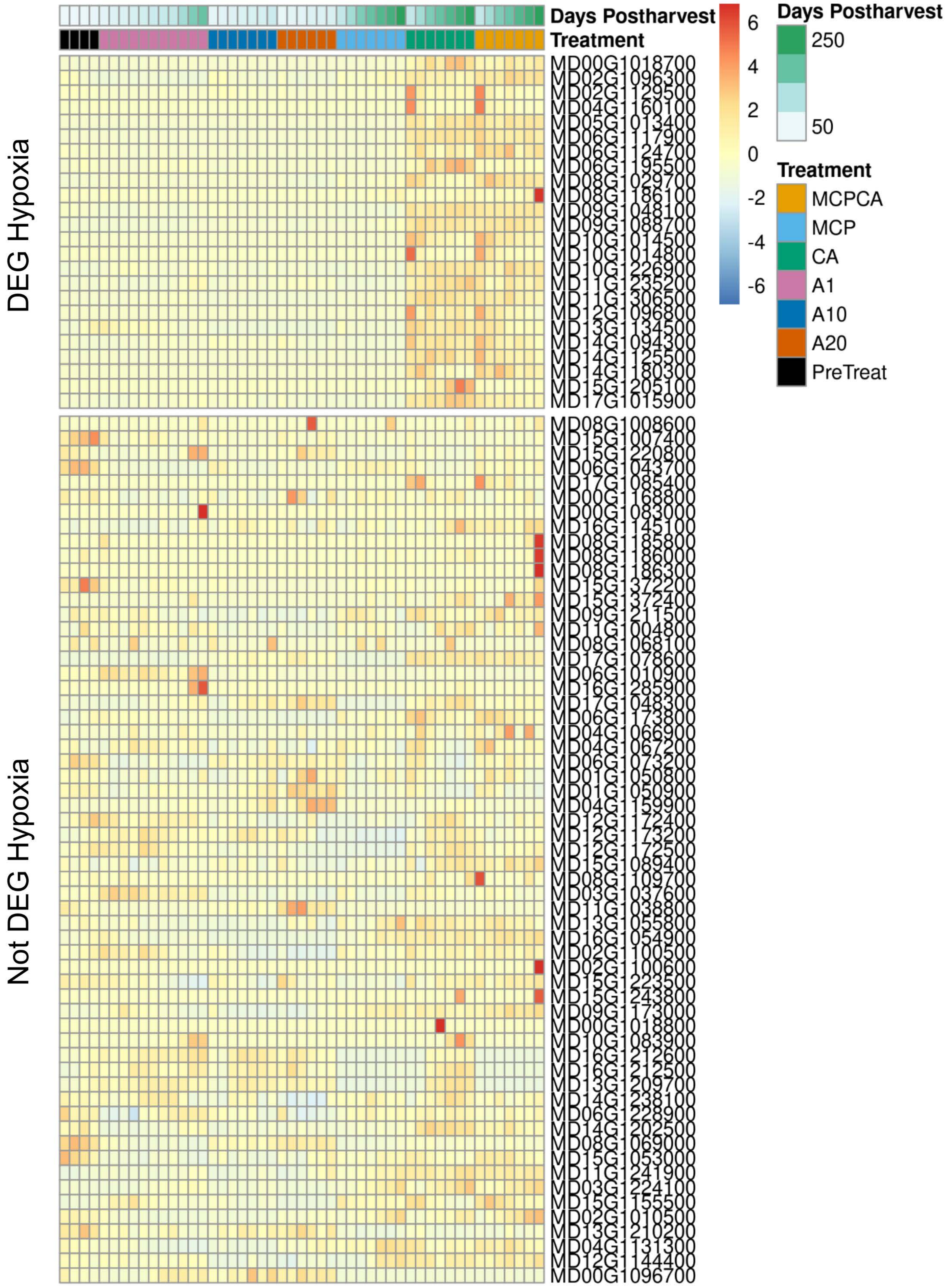

Days Postharvest
Treatment
DEG Hypoxia
Not DEG Hypoxia
MD00G1018700
MD02G1096300
MD02G1129500
MD04G1160100
MD05G1013400
MD06G1117900
MD06G1124700
MD06G1195500
MD08G1029700
MD08G1186100
MD09G1048100
MD09G1088700
MD10G1014500
MD10G1014800
MD10G1226900
MD11G1235200
MD11G1306500
MD12G1096800
MD13G1134500
MD14G1094300
MD14G1125500
MD14G1180300
MD15G1205100
MD17G1015900
MD08G1008600
MD15G1007400
MD15G1220800
MD06G1043700
MD17G1085400
MD00G1168800
MD00G1083000
MD16G1145100
MD08G1185800
MD08G1186000
MD08G1186300
MD15G1372200
MD15G1372400
MD09G1211500
MD11G1004800
MD08G1068100
MD17G1078600
MD06G1010900
MD16G1285900
MD17G1048300
MD06G1173800
MD04G1066900
MD04G1067200
MD06G1073200
MD01G1050800
MD01G1050900
MD04G1159900
MD12G1172400
MD12G1173200
MD12G1172500
MD15G1089400
MD08G1109700
MD03G1037600
MD11G1038800
MD13G1055800
MD16G1054900
MD02G1100500
MD02G1100600
MD15G1223500
MD15G1243800
MD09G1173000
MD00G1018800
MD10G1083900
MD16G1212600
MD16G1212500
MD13G1209700
MD14G1238100
MD06G1228900
MD14G1202500
MD08G1069000
MD15G1053000
MD11G1241900
MD03G1224100
MD15G1155500
MD02G1010500
MD13G1210200
MD04G1131300
MD12G1144400
MD00G1096700
6
4
2
0
-2
-4
-6
Days Postharvest
250
50
Treatment
MCPCA
MCP
CA
A1
A10
A20
PreTreat
