# Supplementary material for: Transcriptomics of long‐term, low oxygen storage coupled with ethylene signaling interference suggests neofunctionalization of hypoxia response pathways in apple ( Malus domestica )
Source: Plant Direct. 2024 Dec 20;8(12):e70025. doi: 10.1002/pld3.70025 (PMC11660084; doi:10.1002/pld3.70025)

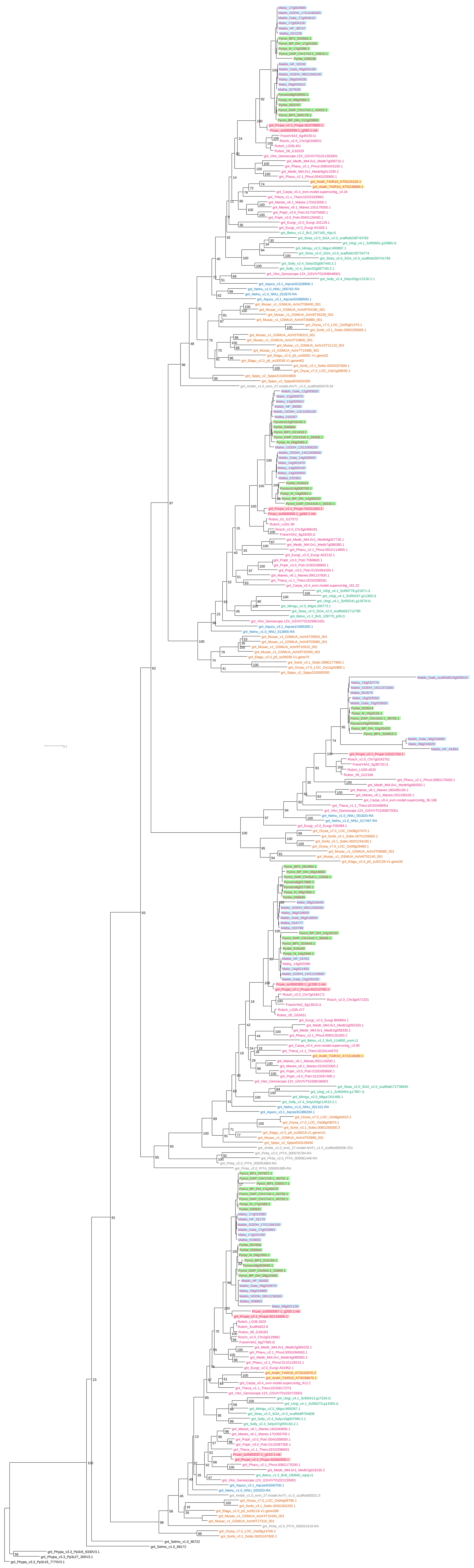

Supplement: Supplementary file 12 — Figure S6: Putative Plant Cysteine Oxidase (PCO) Gene phylogeny. [file PLD3-8-e70025-s024.pdf]
