# Supplementary material for: Transcriptomics of long‐term, low oxygen storage coupled with ethylene signaling interference suggests neofunctionalization of hypoxia response pathways in apple ( Malus domestica )
Source: Plant Direct. 2024 Dec 20;8(12):e70025. doi: 10.1002/pld3.70025 (PMC11660084; doi:10.1002/pld3.70025)
Supplement: Supplementary file 13 — Figure S7: Putative group VII ethylene response factor (ERF VII) Transcription Factor family gene phylogeny. [file PLD3-8-e70025-s009.pdf]

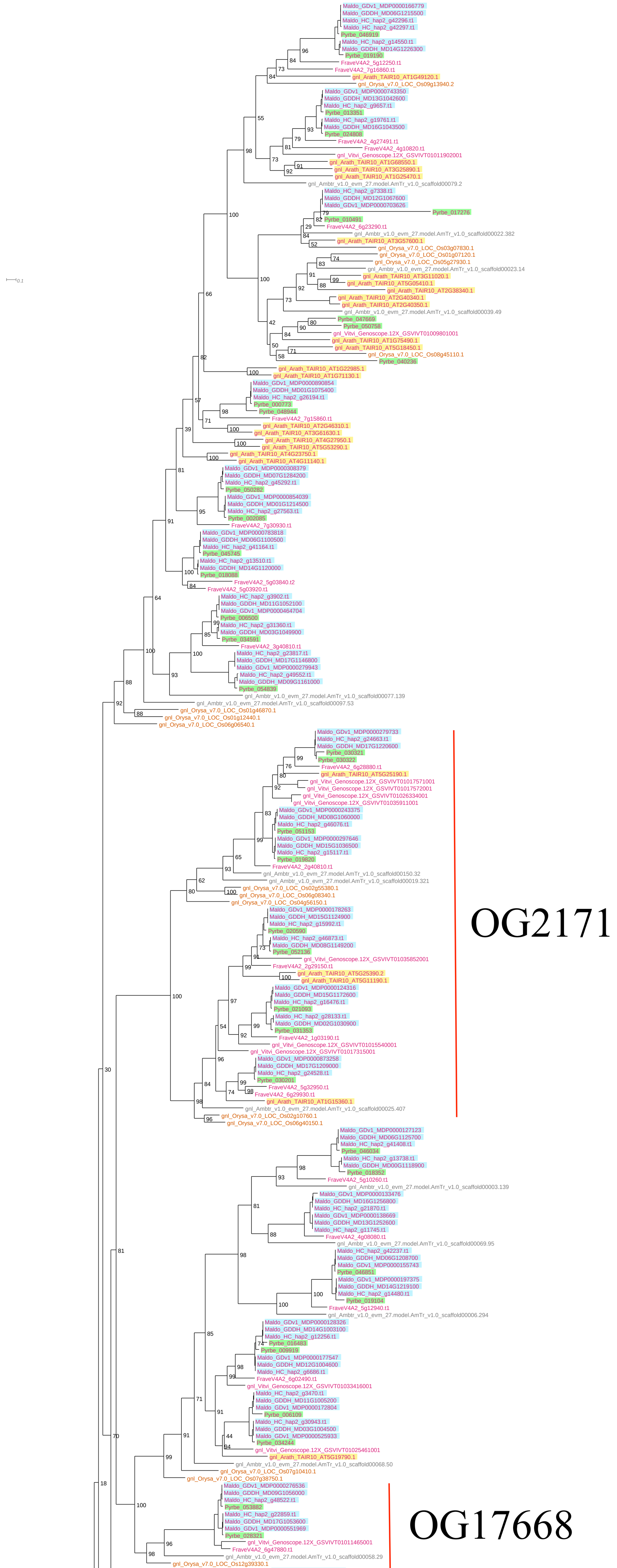
0.2
OG2171
OG17668

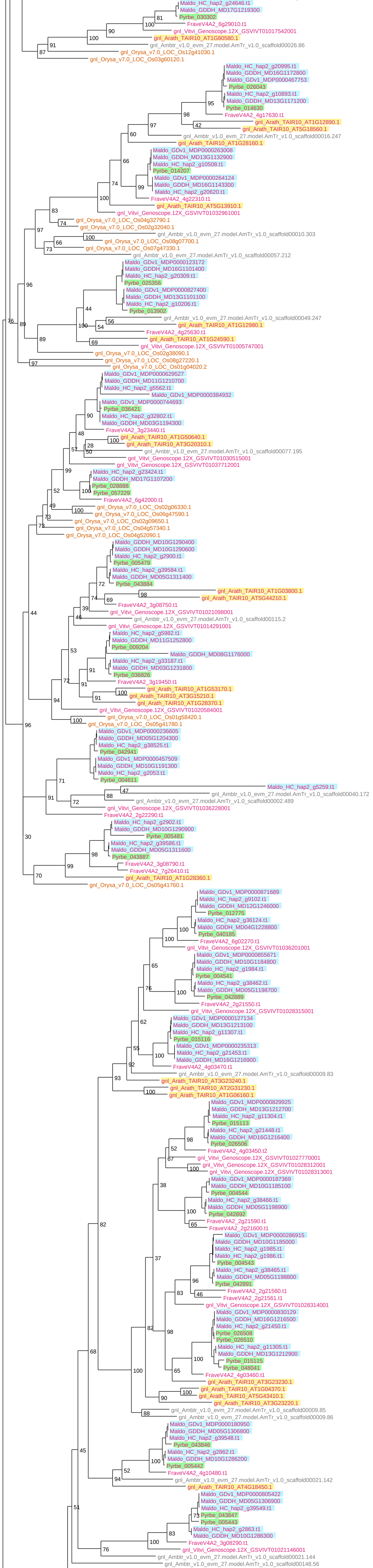

Maldo_HC_hap2_g24646.t1
Maldo_GDDH_MD17G1219300
Pyrbe_030302
FraveV4A2_6g29010.t1
gnl_Vitvi_Genoscope.12X_GSVIVT01017542001
gnl_Arath_TAIR10_AT1G80580.1
gnl_Amborella_v1.0_evm_27.model.AmTr_v1.0_scaffold00026.86
gnl_Orysa_v7.0_LOC_Os12g41030.1
gnl_Orysa_v7.0_LOC_Os03g60120.1
Maldo_HC_hap2_g20995.t1
Maldo_GDDH_MD16G1172800
Maldo_GDv1_MDP0000467753
Pyrbe_026043
Maldo_HC_hap2_g10893.t1
Maldo_GDDH_MD13G1171200
Pyrbe_014630
FraveV4A2_4g17630.t1
gnl_Arath_TAIR10_AT1G12890.1
gnl_Arath_TAIR10_AT5G18560.1
gnl_Amborella_v1.0_evm_27.model.AmTr_v1.0_scaffold00016.247
gnl_Arath_TAIR10_AT1G28160.1
Maldo_GDv1_MDP0000263008
Maldo_GDDH_MD13G1132900
Maldo_HC_hap2_g10508.t1
Pyrbe_014207
Maldo_GDv1_MDP0000264124
Maldo_GDDH_MD16G1143300
Maldo_HC_hap2_g20620.t1
FraveV4A2_4g22310.t1
gnl_Arath_TAIR10_AT5G13910.1
gnl_Vitvi_Genoscope.12X_GSVIVT01032961001
gnl_Orysa_v7.0_LOC_Os04g32790.1
gnl_Orysa_v7.0_LOC_Os02g32040.1
gnl_Amborella_v1.0_evm_27.model.AmTr_v1.0_scaffold00010.303
gnl_Orysa_v7.0_LOC_Os08g07700.1
gnl_Orysa_v7.0_LOC_Os07g47330.1
gnl_Amborella_v1.0_evm_27.model.AmTr_v1.0_scaffold00057.212
Maldo_GDv1_MDP0000123172
Maldo_GDDH_MD16G1101400
Maldo_HC_hap2_g20309.t1
Pyrbe_025356
Maldo_GDv1_MDP0000827400
Maldo_GDDH_MD13G1101100
Maldo_HC_hap2_g10206.t1
Pyrbe_013902
gnl_Amborella_v1.0_evm_27.model.AmTr_v1.0_scaffold00049.247
gnl_Arath_TAIR10_AT1G12980.1
FraveV4A2_4g25630.t1
gnl_Arath_TAIR10_AT1G24590.1
gnl_Vitvi_Genoscope.12X_GSVIVT01005747001
gnl_Orysa_v7.0_LOC_Os02g38090.1
gnl_Orysa_v7.0_LOC_Os08g27220.1
gnl_Orysa_v7.0_LOC_Os01g04020.2
Maldo_GDv1_MDP0000629527
Maldo_GDDH_MD11G1210700
Maldo_HC_hap2_g5562.t1
Maldo_GDv1_MDP0000384932
Maldo_GDv1_MDP0000744693
Pyrbe_036421
Maldo_HC_hap2_g32802.t1
Maldo_GDDH_MD03G1194300
FraveV4A2_3g23440.t1
gnl_Arath_TAIR10_AT1G50640.1
gnl_Arath_TAIR10_AT3G20310.1
gnl_Amborella_v1.0_evm_27.model.AmTr_v1.0_scaffold00077.195
gnl_Vitvi_Genoscope.12X_GSVIVT01030515001
gnl_Vitvi_Genoscope.12X_GSVIVT01037712001
Maldo_HC_hap2_g23424.t1
Maldo_GDDH_MD17G1107200
Pyrbe_028888
Pyrbe_057229
FraveV4A2_6g42000.t1
gnl_Orysa_v7.0_LOC_Os02g06330.1
gnl_Orysa_v7.0_LOC_Os06g47590.1
gnl_Orysa_v7.0_LOC_Os02g09650.1
gnl_Orysa_v7.0_LOC_Os04g57340.1
gnl_Orysa_v7.0_LOC_Os04g52090.1
Maldo_GDDH_MD10G1290400
Maldo_GDDH_MD10G1290600
Maldo_HC_hap2_g2900.t1
Pyrbe_005479
Maldo_HC_hap2_g39584.t1
Maldo_GDDH_MD05G1311400
Pyrbe_043884
gnl_Arath_TAIR10_AT1G03800.1
gnl_Arath_TAIR10_AT5G44210.1
FraveV4A2_3g08750.t1
gnl_Vitvi_Genoscope.12X_GSVIVT01021098001
gnl_Amborella_v1.0_evm_27.model.AmTr_v1.0_scaffold00115.2
gnl_Vitvi_Genoscope.12X_GSVIVT01014291001
Maldo_HC_hap2_g5982.t1
Maldo_GDDH_MD11G1252800
Pyrbe_009204
Maldo_GDDH_MD08G1176000
Maldo_HC_hap2_g33187.t1
Maldo_GDDH_MD03G1231800
Pyrbe_036826
FraveV4A2_3g19450.t1
gnl_Arath_TAIR10_AT1G53170.1
gnl_Arath_TAIR10_AT3G15210.1
gnl_Arath_TAIR10_AT1G28370.1
gnl_Vitvi_Genoscope.12X_GSVIVT01020584001
gnl_Orysa_v7.0_LOC_Os01g58420.1
gnl_Orysa_v7.0_LOC_Os05g41780.1
Maldo_GDv1_MDP0000236605
Maldo_GDDH_MD05G1204300
Maldo_HC_hap2_g38525.t1
Pyrbe_042941
Maldo_GDv1_MDP0000457509
Maldo_GDDH_MD10G1191300
Maldo_HC_hap2_g2053.t1
Pyrbe_004611
Maldo_HC_hap2_g5259.t1
gnl_Amborella_v1.0_evm_27.model.AmTr_v1.0_scaffold00040.172
gnl_Amborella_v1.0_evm_27.model.AmTr_v1.0_scaffold00002.489
gnl_Vitvi_Genoscope.12X_GSVIVT01036228001
FraveV4A2_2g22290.t1
Maldo_HC_hap2_g2902.t1
Maldo_GDDH_MD10G1290900
Pyrbe_005481
Maldo_HC_hap2_g39586.t1
Maldo_GDDH_MD05G1311600
Pyrbe_043887
FraveV4A2_3g08790.t1
FraveV4A2_7g26410.t1
gnl_Arath_TAIR10_AT1G28360.1
gnl_Orysa_v7.0_LOC_Os05g41760.1
Maldo_GDv1_MDP0000871689
Maldo_HC_hap2_g9102.t1
Maldo_GDDH_MD12G1246000
Pyrbe_012775
Maldo_HC_hap2_g36124.t1
Maldo_GDDH_MD04G1228800
Pyrbe_040185
FraveV4A2_6g02270.t1
gnl_Vitvi_Genoscope.12X_GSVIVT01036201001
Maldo_GDv1_MDP0000855671
Maldo_GDDH_MD10G1184800
Maldo_HC_hap2_g1984.t1
Pyrbe_004541
Maldo_HC_hap2_g38462.t1
Maldo_GDDH_MD05G1198700
Pyrbe_042889
FraveV4A2_2g21550.t1
gnl_Vitvi_Genoscope.12X_GSVIVT01028315001
Maldo_GDv1_MDP0000127134
Maldo_GDDH_MD13G1213100
Maldo_HC_hap2_g11307.t1
Pyrbe_015116
Maldo_GDv1_MDP0000235313
Maldo_HC_hap2_g21453.t1
Maldo_GDDH_MD16G1216900
FraveV4A2_4g03470.t1
gnl_Amborella_v1.0_evm_27.model.AmTr_v1.0_scaffold00009.83
gnl_Arath_TAIR10_AT3G23240.1
gnl_Arath_TAIR10_AT2G31230.1
gnl_Arath_TAIR10_AT1G06160.1
Maldo_GDv1_MDP0000829925
Maldo_GDDH_MD13G1212700
Maldo_HC_hap2_g11304.t1
Pyrbe_015113
Maldo_HC_hap2_g21448.t1
Maldo_GDDH_MD16G1216400
Pyrbe_026506
FraveV4A2_4g03450.t2
gnl_Vitvi_Genoscope.12X_GSVIVT01027770001
gnl_Vitvi_Genoscope.12X_GSVIVT01028312001
gnl_Vitvi_Genoscope.12X_GSVIVT01028313001
Maldo_GDv1_MDP0000187369
Maldo_GDDH_MD10G1185100
Pyrbe_004544
Maldo_HC_hap2_g38466.t1
Maldo_GDDH_MD05G1198900
Pyrbe_042892
FraveV4A2_2g21590.t1
FraveV4A2_2g21600.t1
Maldo_GDv1_MDP0000286915
Maldo_GDDH_MD10G1185000
Maldo_HC_hap2_g1985.t1
Maldo_HC_hap2_g1986.t1
Pyrbe_004543
Maldo_HC_hap2_g38465.t1
Maldo_GDDH_MD05G1198800
Pyrbe_042891
FraveV4A2_2g21560.t1
FraveV4A2_2g21561.t1
gnl_Vitvi_Genoscope.12X_GSVIVT01028314001
Maldo_GDv1_MDP0000830129
Maldo_GDDH_MD16G1216500
Maldo_HC_hap2_g21450.t1
Pyrbe_026508
Pyrbe_026510
Maldo_HC_hap2_g11305.t1
Maldo_GDDH_MD13G1212900
Pyrbe_015115
Pyrbe_048041
FraveV4A2_4g03460.t1
gnl_Arath_TAIR10_AT3G23230.1
gnl_Arath_TAIR10_AT1G04370.1
gnl_Arath_TAIR10_AT5G43410.1
gnl_Arath_TAIR10_AT3G23220.1
gnl_Amborella_v1.0_evm_27.model.AmTr_v1.0_scaffold00009.85
gnl_Amborella_v1.0_evm_27.model.AmTr_v1.0_scaffold00009.86
Maldo_GDv1_MDP0000180950
Maldo_GDDH_MD05G1306800
Maldo_HC_hap2_g39548.t1
Pyrbe_043846
Maldo_HC_hap2_g2862.t1
Maldo_GDDH_MD10G1286200
Pyrbe_005442
FraveV4A2_4g10480.t1
gnl_Amborella_v1.0_evm_27.model.AmTr_v1.0_scaffold00021.142
gnl_Arath_TAIR10_AT4G18450.1
Maldo_GDv1_MDP0000805422
Maldo_GDDH_MD05G1306900
Maldo_HC_hap2_g39549.t1
Pyrbe_043847
Pyrbe_005443
Maldo_HC_hap2_g2863.t1
Maldo_GDDH_MD10G1286300
FraveV4A2_3g08290.t1
gnl_Vitvi_Genoscope.12X_GSVIVT01021146001
gnl_Amborella_v1.0_evm_27.model.AmTr_v1.0_scaffold00021.144
gnl_Amborella_v1.0_evm_27.model.AmTr_v1.0_scaffold00148.56

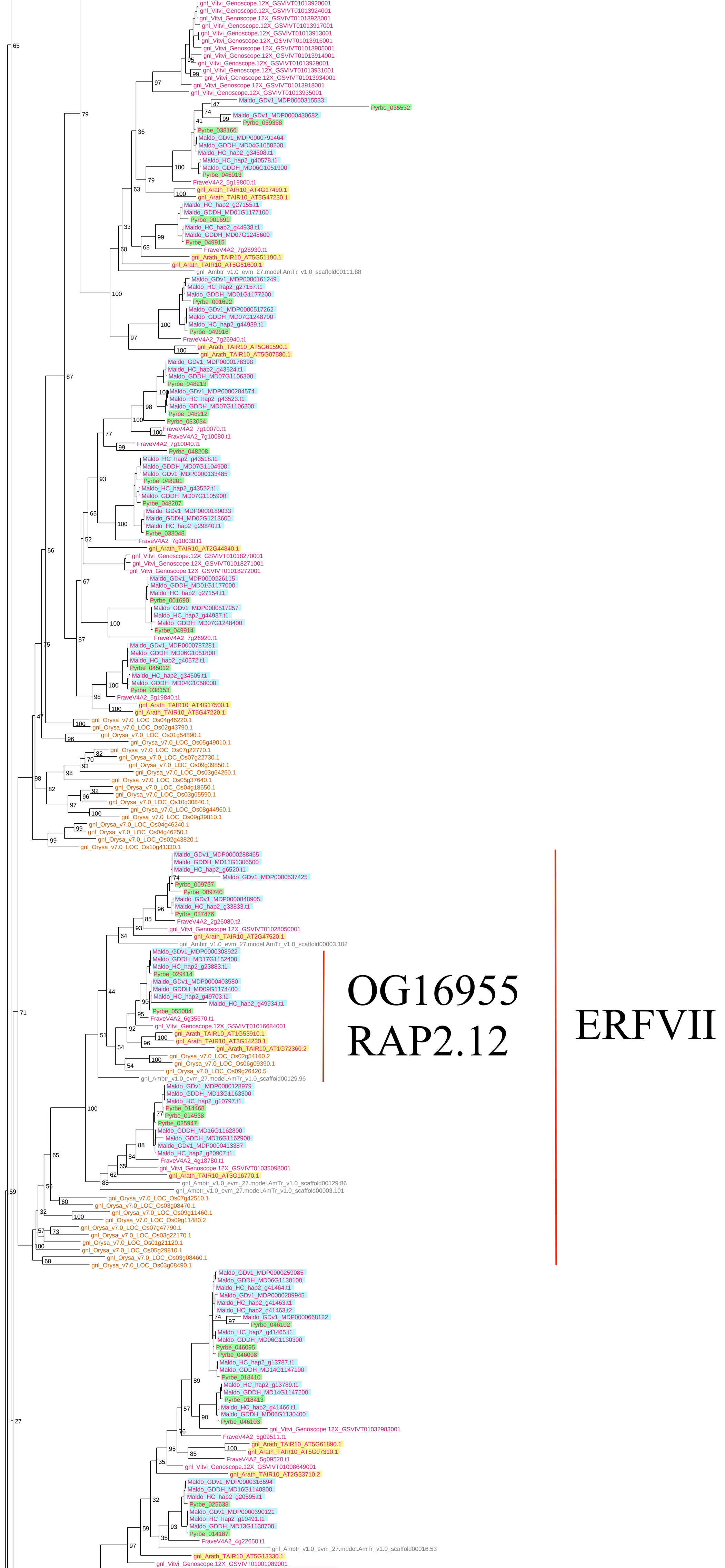
OG16955
RAP2.12
ERFVII

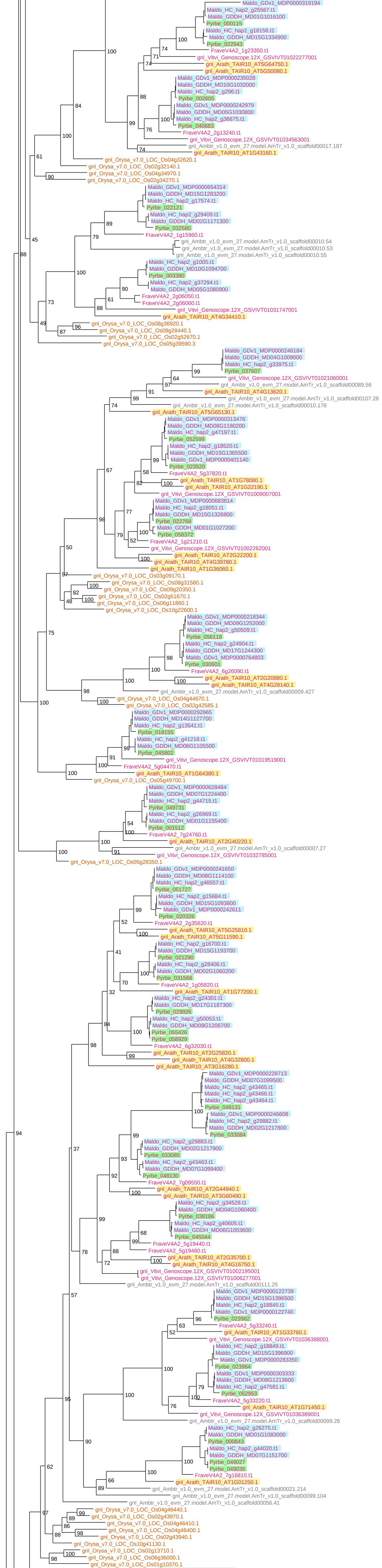
Maldo_GDv1_MDP0000319194
Maldo_HC_hap2_g25567.t1
Maldo_GDDH_MD01G1016100
Pyrbe_000115
Maldo_HC_hap2_g18158.t1
Maldo_GDDH_MD15G1334900
Pyrbe_022943
FraveV4A2_1g23350.t1
gnl_Vitvi_Genoscope.12X_GSVIVT01022277001
gnl_Arath_TAIR10_AT5G64750.1
gnl_Arath_TAIR10_AT5G50080.1
Maldo_GDv1_MDP0000235028
Maldo_GDDH_MD10G1032000
Maldo_HC_hap2_g296.t1
Pyrbe_002605
Maldo_GDv1_MDP0000242979
Maldo_GDDH_MD05G1030800
Maldo_HC_hap2_g36675.t1
Pyrbe_040683
FraveV4A2_2g13240.t1
gnl_Vitvi_Genoscope.12X_GSVIVT01034563001
gnl_Ambtr_v1.0_evm_27.model.AmTr_v1.0_scaffold00017.187
gnl_Arath_TAIR10_AT1G43160.1
gnl_Orysa_v7.0_LOC_Os04g32620.1
gnl_Orysa_v7.0_LOC_Os02g32140.1
gnl_Orysa_v7.0_LOC_Os04g34970.1
gnl_Orysa_v7.0_LOC_Os02g34270.1
Maldo_GDv1_MDP0000654314
Maldo_GDDH_MD15G1283200
Maldo_HC_hap2_g17574.t1
Pyrbe_022121
Maldo_HC_hap2_g29409.t1
Maldo_GDDH_MD02G1171300
Pyrbe_032580
FraveV4A2_1g15960.t1
gnl_Ambtr_v1.0_evm_27.model.AmTr_v1.0_scaffold00010.54
gnl_Ambtr_v1.0_evm_27.model.AmTr_v1.0_scaffold00010.53
gnl_Ambtr_v1.0_evm_27.model.AmTr_v1.0_scaffold00010.55
Maldo_HC_hap2_g1005.t1
Maldo_GDDH_MD10G1094700
Pyrbe_003390
Maldo_HC_hap2_g37294.t1
Maldo_GDDH_MD05G1080900
FraveV4A2_2g06050.t1
FraveV4A2_2g06060.t1
gnl_Vitvi_Genoscope.12X_GSVIVT01031747001
gnl_Arath_TAIR10_AT4G34410.1
gnl_Orysa_v7.0_LOC_Os08g36920.1
gnl_Orysa_v7.0_LOC_Os09g28440.1
gnl_Orysa_v7.0_LOC_Os02g52670.1
gnl_Orysa_v7.0_LOC_Os05g39590.3
Maldo_GDv1_MDP0000246184
Maldo_GDDH_MD04G1009000
Maldo_HC_hap2_g33975.t1
Pyrbe_037607
gnl_Vitvi_Genoscope.12X_GSVIVT01021060001
gnl_Ambtr_v1.0_evm_27.model.AmTr_v1.0_scaffold00089.56
gnl_Arath_TAIR10_AT4G13620.1
gnl_Ambtr_v1.0_evm_27.model.AmTr_v1.0_scaffold00107.26
gnl_Ambtr_v1.0_evm_27.model.AmTr_v1.0_scaffold00010.176
gnl_Arath_TAIR10_AT5G65130.1
Maldo_GDv1_MDP0000313476
Maldo_GDDH_MD08G1180200
Maldo_HC_hap2_g47197.t1
Pyrbe_052599
Maldo_HC_hap2_g18520.t1
Maldo_GDDH_MD15G1365500
Maldo_GDv1_MDP0000401140
Pyrbe_023520
FraveV4A2_5g37820.t1
gnl_Arath_TAIR10_AT1G78080.1
gnl_Arath_TAIR10_AT1G22190.1
gnl_Vitvi_Genoscope.12X_GSVIVT01009007001
Maldo_GDv1_MDP0000683814
Maldo_HC_hap2_g18051.t1
Maldo_GDDH_MD15G1326800
Pyrbe_022768
Maldo_GDDH_MD01G1027200
Pyrbe_058372
FraveV4A2_1g21210.t1
gnl_Vitvi_Genoscope.12X_GSVIVT01002262001
gnl_Arath_TAIR10_AT2G22200.1
gnl_Arath_TAIR10_AT4G39780.1
gnl_Arath_TAIR10_AT1G36060.1
gnl_Orysa_v7.0_LOC_Os03g09170.1
gnl_Orysa_v7.0_LOC_Os08g31580.1
gnl_Orysa_v7.0_LOC_Os09g20350.1
gnl_Orysa_v7.0_LOC_Os02g51670.1
gnl_Orysa_v7.0_LOC_Os06g11860.1
gnl_Orysa_v7.0_LOC_Os10g22600.1
Maldo_GDv1_MDP0000218344
Maldo_GDDH_MD09G1252000
Maldo_HC_hap2_g50509.t1
Pyrbe_056118
Maldo_HC_hap2_g24904.t1
Maldo_GDDH_MD17G1244300
Maldo_GDv1_MDP0000764803
Pyrbe_030601
FraveV4A2_6g26090.t1
gnl_Arath_TAIR10_AT2G20880.1
gnl_Arath_TAIR10_AT4G28140.1
gnl_Ambtr_v1.0_evm_27.model.AmTr_v1.0_scaffold00009.427
gnl_Orysa_v7.0_LOC_Os04g44670.1
gnl_Orysa_v7.0_LOC_Os02g42585.1
Maldo_GDv1_MDP0000292965
Maldo_GDDH_MD14G1127700
Maldo_HC_hap2_g13541.t1
Pyrbe_018155
Maldo_HC_hap2_g41218.t1
Maldo_GDDH_MD06G1105500
Pyrbe_045802
gnl_Vitvi_Genoscope.12X_GSVIVT01019519001
FraveV4A2_5g04470.t1
gnl_Arath_TAIR10_AT1G64380.1
gnl_Orysa_v7.0_LOC_Os05g49700.1
Maldo_GDv1_MDP0000628484
Maldo_GDDH_MD07G1224400
Maldo_HC_hap2_g44715.t1
Pyrbe_049731
Maldo_HC_hap2_g26969.t1
Maldo_GDDH_MD01G1155400
Pyrbe_001512
FraveV4A2_7g24760.t1
gnl_Arath_TAIR10_AT2G40220.1
gnl_Ambtr_v1.0_evm_27.model.AmTr_v1.0_scaffold00007.27
gnl_Vitvi_Genoscope.12X_GSVIVT01032785001
gnl_Orysa_v7.0_LOC_Os05g28350.1
Maldo_GDv1_MDP0000241650
Maldo_GDDH_MD08G1114100
Maldo_HC_hap2_g46557.t1
Pyrbe_051727
Maldo_HC_hap2_g15684.t1
Maldo_GDDH_MD15G1093800
Maldo_GDv1_MDP0000242611
Pyrbe_020326
FraveV4A2_2g35620.t1
gnl_Arath_TAIR10_AT5G25810.1
gnl_Arath_TAIR10_AT5G11590.1
Maldo_HC_hap2_g16700.t1
Maldo_GDDH_MD15G1193700
Pyrbe_021280
Maldo_HC_hap2_g28406.t1
Maldo_GDDH_MD02G1060200
Pyrbe_031568
FraveV4A2_1g05820.t1
gnl_Arath_TAIR10_AT1G77200.1
Maldo_HC_hap2_g24301.t1
Maldo_GDDH_MD17G1187300
Pyrbe_029926
Maldo_HC_hap2_g50053.t1
Maldo_GDDH_MD09G1206700
Pyrbe_055426
Pyrbe_058929
FraveV4A2_6g32030.t1
gnl_Arath_TAIR10_AT2G25820.1
gnl_Arath_TAIR10_AT4G32800.1
gnl_Arath_TAIR10_AT3G16280.1
Maldo_GDv1_MDP0000228713
Maldo_GDDH_MD07G1099500
Maldo_HC_hap2_g43465.t1
Maldo_HC_hap2_g43466.t1
Maldo_HC_hap2_g43464.t1
Pyrbe_048131
Maldo_GDv1_MDP0000246608
Maldo_HC_hap2_g29882.t1
Maldo_GDDH_MD02G1217800
Pyrbe_033084
Maldo_HC_hap2_g29883.t1
Maldo_GDDH_MD02G1217900
Pyrbe_033085
Maldo_HC_hap2_g43463.t1
Maldo_GDDH_MD07G1099400
Pyrbe_048130
FraveV4A2_7g09550.t1
gnl_Arath_TAIR10_AT2G44940.1
gnl_Arath_TAIR10_AT3G60490.1
Maldo_HC_hap2_g34528.t1
Maldo_GDDH_MD04G1060400
Pyrbe_038186
Maldo_HC_hap2_g40605.t1
Maldo_GDDH_MD06G1053600
Pyrbe_045044
FraveV4A2_5g19440.t1
FraveV4A2_5g19460.t1
gnl_Arath_TAIR10_AT2G35700.1
gnl_Arath_TAIR10_AT4G16750.1
gnl_Vitvi_Genoscope.12X_GSVIVT01002195001
gnl_Vitvi_Genoscope.12X_GSVIVT01006277001
gnl_Ambtr_v1.0_evm_27.model.AmTr_v1.0_scaffold00111.25
Maldo_GDv1_MDP0000122739
Maldo_GDDH_MD15G1396500
Maldo_HC_hap2_g18845.t1
Maldo_GDv1_MDP0000122740
Pyrbe_023962
FraveV4A2_5g33240.t1
gnl_Arath_TAIR10_AT1G33760.1
gnl_Vitvi_Genoscope.12X_GSVIVT01036388001
Maldo_HC_hap2_g18849.t1
Maldo_GDDH_MD15G1396900
Maldo_GDv1_MDP0000283350
Pyrbe_023964
Maldo_GDv1_MDP0000303333
Maldo_GDDH_MD08G1213600
Maldo_HC_hap2_g47581.t1
Pyrbe_052953
FraveV4A2_5g33220.t1
gnl_Arath_TAIR10_AT1G71450.1
gnl_Vitvi_Genoscope.12X_GSVIVT01036389001
gnl_Ambtr_v1.0_evm_27.model.AmTr_v1.0_scaffold00099.26
Maldo_HC_hap2_g26275.t1
Maldo_GDDH_MD01G1083000
Pyrbe_000843
Maldo_HC_hap2_g44020.t1
Maldo_GDDH_MD07G1151700
Pyrbe_049027
Pyrbe_049035
FraveV4A2_7g16810.t1
gnl_Arath_TAIR10_AT1G01250.1
gnl_Ambtr_v1.0_evm_27.model.AmTr_v1.0_scaffold00021.214
gnl_Ambtr_v1.0_evm_27.model.AmTr_v1.0_scaffold00099.104
gnl_Ambtr_v1.0_evm_27.model.AmTr_v1.0_scaffold00056.41
gnl_Orysa_v7.0_LOC_Os04g46440.1
gnl_Orysa_v7.0_LOC_Os02g43970.1
gnl_Orysa_v7.0_LOC_Os04g46410.1
gnl_Orysa_v7.0_LOC_Os04g46400.1
gnl_Orysa_v7.0_LOC_Os02g43940.1
gnl_Orysa_v7.0_LOC_Os10g41130.1
gnl_Orysa_v7.0_LOC_Os02g13710.1
gnl_Orysa_v7.0_LOC_Os06g36000.1
gnl_Orysa_v7.0_LOC_Os01g10370.1

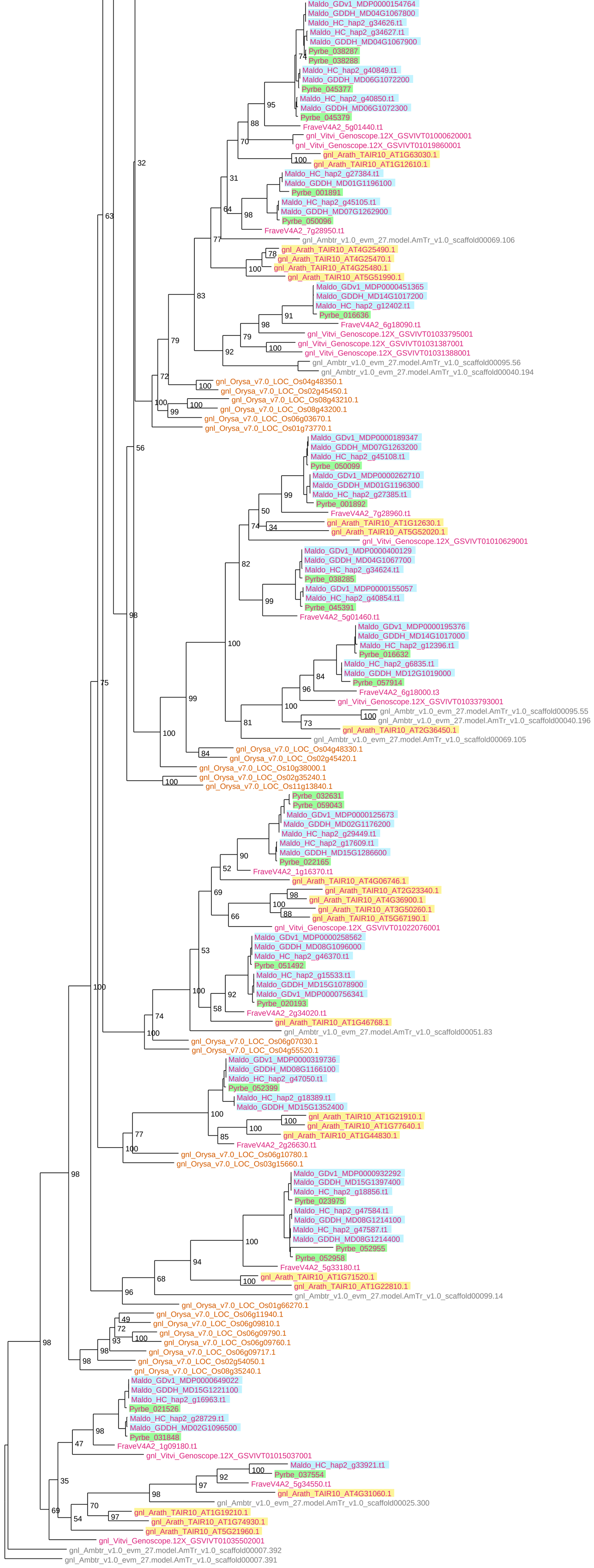

Maldo_GDv1_MDP0000154764
Maldo_GDDH_MD04G1067800
Maldo_HC_hap2_g34626.t1
Maldo_HC_hap2_g34627.t1
Maldo_GDDH_MD04G1067900
Pyrbe_038287
Pyrbe_038288
Maldo_HC_hap2_g40849.t1
Maldo_GDDH_MD06G1072200
Pyrbe_045377
Maldo_HC_hap2_g40850.t1
Maldo_GDDH_MD06G1072300
Pyrbe_045379
FraveV4A2_5g01440.t1
gnl_Vitvi_Genoscope.12X_GSVIVT01000620001
gnl_Vitvi_Genoscope.12X_GSVIVT01019860001
gnl_Arath_TAIR10_AT1G63030.1
gnl_Arath_TAIR10_AT1G12610.1
Maldo_HC_hap2_g27384.t1
Maldo_GDDH_MD01G1196100
Pyrbe_001891
Maldo_HC_hap2_g45105.t1
Maldo_GDDH_MD07G1262900
Pyrbe_050096
FraveV4A2_7g28950.t1
gnl_Ambtr_v1.0_evm_27.model.AmTr_v1.0_scaffold00069.106
gnl_Arath_TAIR10_AT4G25490.1
gnl_Arath_TAIR10_AT4G25470.1
gnl_Arath_TAIR10_AT4G25480.1
gnl_Arath_TAIR10_AT5G51990.1
Maldo_GDv1_MDP0000451365
Maldo_GDDH_MD14G1017200
Maldo_HC_hap2_g12402.t1
Pyrbe_016636
FraveV4A2_6g18090.t1
gnl_Vitvi_Genoscope.12X_GSVIVT01033795001
gnl_Vitvi_Genoscope.12X_GSVIVT01031387001
gnl_Vitvi_Genoscope.12X_GSVIVT01031388001
gnl_Ambtr_v1.0_evm_27.model.AmTr_v1.0_scaffold00095.56
gnl_Ambtr_v1.0_evm_27.model.AmTr_v1.0_scaffold00040.194
gnl_Orysa_v7.0_LOC_Os04g48350.1
gnl_Orysa_v7.0_LOC_Os02g45450.1
gnl_Orysa_v7.0_LOC_Os08g43210.1
gnl_Orysa_v7.0_LOC_Os08g43200.1
gnl_Orysa_v7.0_LOC_Os06g03670.1
gnl_Orysa_v7.0_LOC_Os01g73770.1
Maldo_GDv1_MDP0000189347
Maldo_GDDH_MD07G1263200
Maldo_HC_hap2_g45108.t1
Pyrbe_050099
Maldo_GDv1_MDP0000262710
Maldo_GDDH_MD01G1196300
Maldo_HC_hap2_g27385.t1
Pyrbe_001892
FraveV4A2_7g28960.t1
gnl_Arath_TAIR10_AT1G12630.1
gnl_Arath_TAIR10_AT5G52020.1
gnl_Vitvi_Genoscope.12X_GSVIVT01010629001
Maldo_GDv1_MDP0000400129
Maldo_GDDH_MD04G1067700
Maldo_HC_hap2_g34624.t1
Pyrbe_038285
Maldo_GDv1_MDP0000155057
Maldo_HC_hap2_g40854.t1
Pyrbe_045391
FraveV4A2_5g01460.t1
Maldo_GDv1_MDP0000195376
Maldo_GDDH_MD14G1017000
Maldo_HC_hap2_g12396.t1
Pyrbe_016632
Maldo_HC_hap2_g6835.t1
Maldo_GDDH_MD12G1019000
Pyrbe_057914
FraveV4A2_6g18000.t3
gnl_Vitvi_Genoscope.12X_GSVIVT01033793001
gnl_Ambtr_v1.0_evm_27.model.AmTr_v1.0_scaffold00095.55
gnl_Ambtr_v1.0_evm_27.model.AmTr_v1.0_scaffold00040.196
gnl_Arath_TAIR10_AT2G36450.1
gnl_Ambtr_v1.0_evm_27.model.AmTr_v1.0_scaffold00069.105
gnl_Orysa_v7.0_LOC_Os04g48330.1
gnl_Orysa_v7.0_LOC_Os02g45420.1
gnl_Orysa_v7.0_LOC_Os10g38000.1
gnl_Orysa_v7.0_LOC_Os02g35240.1
gnl_Orysa_v7.0_LOC_Os11g13840.1
Pyrbe_032631
Pyrbe_059043
Maldo_GDv1_MDP0000125673
Maldo_GDDH_MD02G1176200
Maldo_HC_hap2_g29449.t1
Maldo_HC_hap2_g17609.t1
Maldo_GDDH_MD15G1286600
Pyrbe_022165
FraveV4A2_1g16370.t1
gnl_Arath_TAIR10_AT4G06746.1
gnl_Arath_TAIR10_AT2G23340.1
gnl_Arath_TAIR10_AT4G36900.1
gnl_Arath_TAIR10_AT3G50260.1
gnl_Arath_TAIR10_AT5G67190.1
gnl_Vitvi_Genoscope.12X_GSVIVT01022076001
Maldo_GDv1_MDP0000258562
Maldo_GDDH_MD08G1096000
Maldo_HC_hap2_g46370.t1
Pyrbe_051492
Maldo_HC_hap2_g15533.t1
Maldo_GDDH_MD15G1078900
Maldo_GDv1_MDP0000756341
Pyrbe_020193
FraveV4A2_2g34020.t1
gnl_Arath_TAIR10_AT1G46768.1
gnl_Ambtr_v1.0_evm_27.model.AmTr_v1.0_scaffold00051.83
gnl_Orysa_v7.0_LOC_Os06g07030.1
gnl_Orysa_v7.0_LOC_Os04g55520.1
Maldo_GDv1_MDP0000319736
Maldo_GDDH_MD08G1166100
Maldo_HC_hap2_g47050.t1
Pyrbe_052399
Maldo_HC_hap2_g18389.t1
Maldo_GDDH_MD15G1352400
gnl_Arath_TAIR10_AT1G21910.1
gnl_Arath_TAIR10_AT1G77640.1
gnl_Arath_TAIR10_AT1G44830.1
FraveV4A2_2g26630.t1
gnl_Orysa_v7.0_LOC_Os06g10780.1
gnl_Orysa_v7.0_LOC_Os03g15660.1
Maldo_GDv1_MDP0000932292
Maldo_GDDH_MD15G1397400
Maldo_HC_hap2_g18856.t1
Pyrbe_023975
Maldo_HC_hap2_g47584.t1
Maldo_GDDH_MD08G1214100
Maldo_HC_hap2_g47587.t1
Maldo_GDDH_MD08G1214400
Pyrbe_052955
Pyrbe_052958
FraveV4A2_5g33180.t1
gnl_Arath_TAIR10_AT1G71520.1
gnl_Arath_TAIR10_AT1G22810.1
gnl_Ambtr_v1.0_evm_27.model.AmTr_v1.0_scaffold00099.14
gnl_Orysa_v7.0_LOC_Os01g66270.1
gnl_Orysa_v7.0_LOC_Os06g11940.1
gnl_Orysa_v7.0_LOC_Os06g09810.1
gnl_Orysa_v7.0_LOC_Os06g09790.1
gnl_Orysa_v7.0_LOC_Os06g09760.1
gnl_Orysa_v7.0_LOC_Os06g09717.1
gnl_Orysa_v7.0_LOC_Os02g54050.1
gnl_Orysa_v7.0_LOC_Os08g35240.1
Maldo_GDv1_MDP0000649022
Maldo_GDDH_MD15G1221100
Maldo_HC_hap2_g16963.t1
Pyrbe_021526
Maldo_HC_hap2_g28729.t1
Maldo_GDDH_MD02G1096500
Pyrbe_031848
FraveV4A2_1g09180.t1
gnl_Vitvi_Genoscope.12X_GSVIVT01015037001
Maldo_HC_hap2_g33921.t1
Pyrbe_037554
FraveV4A2_5g34550.t1
gnl_Arath_TAIR10_AT4G31060.1
gnl_Ambtr_v1.0_evm_27.model.AmTr_v1.0_scaffold00025.300
gnl_Arath_TAIR10_AT1G19210.1
gnl_Arath_TAIR10_AT1G74930.1
gnl_Arath_TAIR10_AT5G21960.1
gnl_Vitvi_Genoscope.12X_GSVIVT01035502001
gnl_Ambtr_v1.0_evm_27.model.AmTr_v1.0_scaffold00007.392
gnl_Ambtr_v1.0_evm_27.model.AmTr_v1.0_scaffold00007.391
