# Supplementary material for: Transcriptomics of long‐term, low oxygen storage coupled with ethylene signaling interference suggests neofunctionalization of hypoxia response pathways in apple ( Malus domestica )
Source: Plant Direct. 2024 Dec 20;8(12):e70025. doi: 10.1002/pld3.70025 (PMC11660084; doi:10.1002/pld3.70025)
Supplement: Supplementary file 16 — Figure S10: MD16G1162800 and MD16G1162900 Malus and Pyrus gene phylogeny. [file PLD3-8-e70025-s021.pdf]

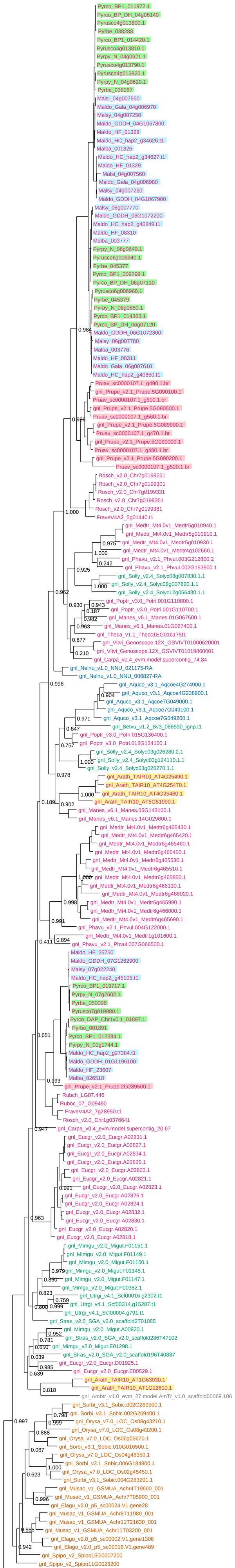

Pyrco_BP1_011972.1
Pyrco_BP_DH_04g06140
Pyrusco4g013800.1
Pyrbe_038288
Pyrco_BP1_014420.1
Pyrusco4g013810.1
Pyrpy_N_04g0621.1
Pyrusco4g013790.1
Pyrusco4g013820.1
Pyrpy_N_04g0620.1
Pyrbe_038287
Malsi_04g007550
Maldo_Gala_04g006970
Malsy_04g007250
Maldo_GDDH_04G1067800
Maldo_HF_01328
Maldo_HC_hap2_g34626.t1
Malba_001826
Maldo_HC_hap2_g34627.t1
Maldo_HF_01329
Malsi_04g007560
Maldo_Gala_04g006980
Malsy_04g007260
Maldo_GDDH_04G1067900
Malsy_06g007770
Maldo_GDDH_06G1072200
Maldo_HC_hap2_g40849.t1
Maldo_HF_08310
Malba_003777
Pyrpy_N_06g0649.1
Pyrusco6g006940.1
Pyrbe_045377
Pyrco_BP1_009299.1
Pyrco_BP_DH_06g07110
Pyrusco6g006960.1
Pyrbe_045379
Pyrpy_N_06g0650.1
Pyrco_BP1_014363.1
Pyrco_BP_DH_06g07120
0.984
Maldo_GDDH_06G1072300
Malsy_06g007780
Malba_003776
Maldo_HF_08311
Maldo_Gala_06g007610
Maldo_HC_hap2_g40850.t1
Pruav_sc0000107.1_g490.1.br
gnl_Prupe_v2.1_Prupe.5G090100.1
Pruav_sc0000107.1_g510.1.br
gnl_Prupe_v2.1_Prupe.5G090500.1
Pruav_sc0000107.1_g560.1.br
0.986
gnl_Prupe_v2.1_Prupe.5G089900.1
Pruav_sc0000107.1_g470.1.br
gnl_Prupe_v2.1_Prupe.5G090000.1
Pruav_sc0000107.1_g480.1.br
gnl_Prupe_v2.1_Prupe.5G090200.1
Pruav_sc0000107.1_g520.1.br
Rosch_v2.0_Chr7g0199251
Rosch_v2.0_Chr7g0199301
Rosch_v2.0_Chr7g0199331
Rosch_v2.0_Chr7g0199351
Rosch_v2.0_Chr7g0199381
1.000
FraveV4A2_5g01440.t1
gnl_Medtr_Mt4.0v1_Medtr5g010940.1
gnl_Medtr_Mt4.0v1_Medtr5g010910.1
0.975
gnl_Medtr_Mt4.0v1_Medtr5g010930.1
gnl_Medtr_Mt4.0v1_Medtr4g102660.1
1.000
gnl_Phavu_v2.1_Phvul.003G212800.2
0.242
gnl_Phavu_v2.1_Phvul.002G153900.1
0.925
gnl_Solly_v2.4_Solyc08g007830.1.1
1.000
gnl_Solly_v2.4_Solyc08g007820.1.1
gnl_Solly_v2.4_Solyc12g056430.1.1
0.962
gnl_Poptr_v3.0_Potri.001G110800.1
0.930
0.943
0.187
gnl_Poptr_v3.0_Potri.001G110700.1
0.982
gnl_Manes_v6.1_Manes.01G067500.1
0.963
gnl_Manes_v6.1_Manes.01G067400.1
gnl_Theca_v1.1_Thecc1EG016175t1
0.877
gnl_Vitvi_Genoscope.12X_GSVIVT01000620001
0.210
gnl_Vitvi_Genoscope.12X_GSVIVT01019860001
gnl_Carpa_v0.4_evm.model.supercontig_74.84
gnl_Nelnu_v1.0_NNU_021175-RA
gnl_Nelnu_v1.0_NNU_006827-RA
0.996
gnl_Aquco_v3.1_Aqcoe4G274900.1
gnl_Aquco_v3.1_Aqcoe4G238900.1
0.904
gnl_Aquco_v3.1_Aqcoe7G049000.1
gnl_Aquco_v3.1_Aqcoe7G049100.1
0.971
gnl_Aquco_v3.1_Aqcoe7G049200.1
gnl_Betvu_v1.2_Bv3_066590_ignp.t1
0.647
gnl_Poptr_v3.0_Potri.015G136400.1
0.757
gnl_Poptr_v3.0_Potri.012G134100.1
gnl_Solly_v2.4_Solyc03g026280.2.1
1.000
gnl_Solly_v2.4_Solyc03g124110.1.1
gnl_Solly_v2.4_Solyc03g026270.1.1
0.978
gnl_Arath_TAIR10_AT4G25490.1
gnl_Arath_TAIR10_AT4G25470.1
1.000
gnl_Arath_TAIR10_AT4G25480.1
0.189
0.902
gnl_Arath_TAIR10_AT5G51990.1
gnl_Manes_v6.1_Manes.06G143100.1
gnl_Manes_v6.1_Manes.14G029600.1
gnl_Medtr_Mt4.0v1_Medtr6g465430.1
gnl_Medtr_Mt4.0v1_Medtr6g465420.1
gnl_Medtr_Mt4.0v1_Medtr6g465460.1
gnl_Medtr_Mt4.0v1_Medtr6g465450.1
gnl_Medtr_Mt4.0v1_Medtr6g465530.1
gnl_Medtr_Mt4.0v1_Medtr6g465510.1
1.000
gnl_Medtr_Mt4.0v1_Medtr6g465850.1
gnl_Medtr_Mt4.0v1_Medtr6g466130.1
gnl_Medtr_Mt4.0v1_Medtr6g466020.1
0.998
gnl_Medtr_Mt4.0v1_Medtr6g465990.1
gnl_Medtr_Mt4.0v1_Medtr6g466000.1
0.991
gnl_Medtr_Mt4.0v1_Medtr6g465690.1
gnl_Phavu_v2.1_Phvul.004G122000.1
gnl_Medtr_Mt4.0v1_Medtr1g101600.1
0.411
0.894
gnl_Phavu_v2.1_Phvul.007G066500.1
Maldo_HF_25750
Maldo_GDDH_07G1262900
Malsy_07g022240
Maldo_HC_hap2_g45105.t1
Pyrco_BP1_019717.1
Pyrpy_N_07g2602.1
Pyrbe_050096
Pyrusco7g019980.1
Pyrco_DAP_Chr1v0.1_01897.1
Pyrbe_001891
0.651
Pyrco_BP1_012284.1
Pyrpy_N_01g1744.1
Maldo_HC_hap2_g27384.t1
Maldo_GDDH_01G1196100
Maldo_HF_23607
Malba_026518
0.993
gnl_Prupe_v2.1_Prupe.2G289500.1
Rubch_LG07.446
Ruboc_07_G09490
FraveV4A2_7g28950.t1
Rosch_v2.0_Chr1g0376641
0.947
gnl_Carpa_v0.4_evm.model.supercontig_20.67
gnl_Eucgr_v2.0_Eucgr.A02831.1
gnl_Eucgr_v2.0_Eucgr.A02827.1
gnl_Eucgr_v2.0_Eucgr.A02834.1
gnl_Eucgr_v2.0_Eucgr.A02825.1
gnl_Eucgr_v2.0_Eucgr.A02822.1
gnl_Eucgr_v2.0_Eucgr.A02821.1
0.991
gnl_Eucgr_v2.0_Eucgr.A02823.1
gnl_Eucgr_v2.0_Eucgr.A02826.1
gnl_Eucgr_v2.0_Eucgr.A02824.1
gnl_Eucgr_v2.0_Eucgr.A02832.1
0.963
gnl_Eucgr_v2.0_Eucgr.A02830.1
gnl_Eucgr_v2.0_Eucgr.A02820.1
gnl_Eucgr_v2.0_Eucgr.A02818.1
gnl_Mimgu_v2.0_Migut.F01151.1
gnl_Mimgu_v2.0_Migut.F01149.1
gnl_Mimgu_v2.0_Migut.F01150.1
0.979
gnl_Mimgu_v2.0_Migut.F01148.1
0.850
gnl_Mimgu_v2.0_Migut.F01147.1
gnl_Mimgu_v2.0_Migut.F00382.1
0.823
gnl_Utrgi_v4.1_Scf00016.g2302.t1
0.759
0.800
0.999
gnl_Utrgi_v4.1_Scf00314.g15287.t1
gnl_Utrgi_v4.1_Scf00004.g791.t1
gnl_Stras_v2.0_SGA_v2.0_scaffold2T01085
gnl_Mimgu_v2.0_Migut.A00920.1
0.952
gnl_Stras_v2.0_SGA_v2.0_scaffold286T47102
0.781
0.650
gnl_Mimgu_v2.0_Migut.E01298.1
0.039
gnl_Stras_v2.0_SGA_v2.0_scaffold196T40887
gnl_Eucgr_v2.0_Eucgr.D01925.1
0.985
0.639
gnl_Eucgr_v2.0_Eucgr.E00529.1
gnl_Arath_TAIR10_AT1G63030.1
gnl_Arath_TAIR10_AT1G12610.1
0.818
gnl_Ambtr_v1.0_evm_27.model.AmTr_v1.0_scaffold00069.106
gnl_Sorbi_v3.1_Sobic.002G269500.1
0.798
gnl_Sorbi_v3.1_Sobic.002G269400.1
0.997
0.999
gnl_Orysa_v7.0_LOC_Os08g43210.1
0.888
gnl_Orysa_v7.0_LOC_Os08g43200.1
gnl_Orysa_v7.0_LOC_Os06g03670.1
gnl_Sorbi_v3.1_Sobic.010G016500.1
0.067
gnl_Orysa_v7.0_LOC_Os04g48350.1
gnl_Sorbi_v3.1_Sobic.006G184800.1
1.000
0.623
gnl_Orysa_v7.0_LOC_Os02g45450.1
gnl_Sorbi_v3.1_Sobic.004G283201.1
gnl_Musac_v1_GSMUA_Achr4T19660_001
0.996
gnl_Musac_v1_GSMUA_Achr7T05900_001
gnl_Elagu_v2.0_p5_sc00024.V1.gene28
gnl_Musac_v1_GSMUA_Achr8T11980_001
gnl_Musac_v1_GSMUA_Achr11T21630_001
0.555
gnl_Musac_v1_GSMUA_Achr11T03200_001
gnl_Elagu_v2.0_p5_sc00002.V1.gene1308
0.942
gnl_Elagu_v2.0_p5_sc00016.V1.gene489
gnl_Spipo_v2_Spipo16G0007200
gnl_Spipo_v2_Spipo11G0028200

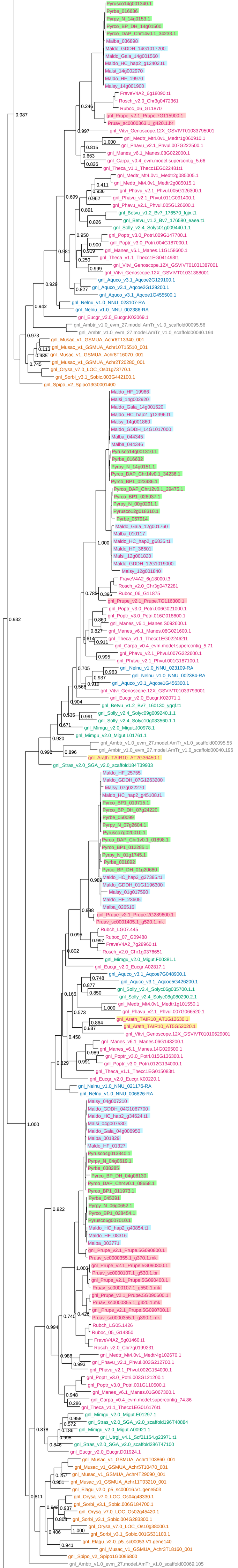

Pyrusco14g001340.1
Pyrbe_016638
Pyrpy_N_14g0153.1
Pyrco_BP_DH_14g01500
Pyrco_DAP_Chr14v0.1_34233.1
Malba_036808
Maldo_GDDH_14G1017200
Maldo_Gala_14g001560
Maldo_HC_hap2_g12402.t1
Malsi_14g002970
Maldo_HF_19970
Malsy_14g001900
FraveV4A2_6g18090.t1
Rosch_v2.0_Chr3g0472361
Ruboc_06_G11870
0.246
gnl_Prupe_v2.1_Prupe.7G115900.1
0.987
Pruav_sc0000363.1_g420.1.br
0.997
gnl_Vitvi_Genoscope.12X_GSVIVT01033795001
gnl_Medtr_Mt4.0v1_Medtr1g060910.1
1.000
0.815
gnl_Phavu_v2.1_Phvul.007G222500.1
gnl_Manes_v6.1_Manes.08G022000.1
0.663
gnl_Carpa_v0.4_evm.model.supercontig_5.66
0.826
gnl_Theca_v1.1_Thecc1EG022481t1
gnl_Medtr_Mt4.0v1_Medtr2g085005.1
0.411
gnl_Medtr_Mt4.0v1_Medtr2g085015.1
0.936
gnl_Phavu_v2.1_Phvul.005G126300.1
0.699
0.962
gnl_Phavu_v2.1_Phvul.011G091400.1
gnl_Phavu_v2.1_Phvul.005G126600.1
0.891
gnl_Betvu_v1.2_Bv7_176570_fgjx.t1
0.826
gnl_Betvu_v1.2_Bv7_176580_eaea.t1
gnl_Solly_v2.4_Solyc01g009440.1.1
0.950
gnl_Poptr_v3.0_Potri.009G147700.1
0.900
gnl_Poptr_v3.0_Potri.004G187000.1
0.981
0.919
gnl_Manes_v6.1_Manes.11G158600.1
0.250
gnl_Theca_v1.1_Thecc1EG041493t1
gnl_Vitvi_Genoscope.12X_GSVIVT01031387001
0.999
gnl_Vitvi_Genoscope.12X_GSVIVT01031388001
0.929
gnl_Aquco_v3.1_Aqcoe2G129100.1
0.827
gnl_Aquco_v3.1_Aqcoe2G129200.1
gnl_Aquco_v3.1_Aqcoe1G455500.1
0.942
gnl_Nelnu_v1.0_NNU_023107-RA
gnl_Nelnu_v1.0_NNU_002386-RA
gnl_Eucgr_v2.0_Eucgr.K02069.1
gnl_Ambtr_v1.0_evm_27.model.AmTr_v1.0_scaffold00095.56
0.973
gnl_Ambtr_v1.0_evm_27.model.AmTr_v1.0_scaffold00040.194
gnl_Musac_v1_GSMUA_Achr6T13340_001
0.113
gnl_Musac_v1_GSMUA_Achr10T15510_001
0.985
gnl_Musac_v1_GSMUA_Achr8T16070_001
0.745
gnl_Musac_v1_GSMUA_Achr2T20280_001
gnl_Orysa_v7.0_LOC_Os01g73770.1
gnl_Sorbi_v3.1_Sobic.003G442100.1
gnl_Spipo_v2_Spipo13G0001400
Maldo_HF_19966
Malsi_14g002920
Maldo_Gala_14g001520
Maldo_HC_hap2_g12396.t1
Malsy_14g001860
Maldo_GDDH_14G1017000
Malba_044345
Malba_044346
Pyrusco14g001310.1
Pyrbe_016632
Pyrpy_N_14g0151.1
Pyrco_DAP_Chr14v0.1_34236.1
Pyrco_BP1_023436.1
Pyrco_DAP_Chr12v0.1_29475.1
Pyrco_BP1_026937.1
Pyrpy_N_00g0291.1
Pyrusco12g018310.1
Pyrbe_057914
Maldo_Gala_12g001760
Malba_010117
1.000
Maldo_HC_hap2_g6835.t1
Maldo_HF_36501
Malsi_12g001820
Maldo_GDDH_12G1019000
Malsy_12g001840
FraveV4A2_6g18000.t3
Rosch_v2.0_Chr3g0472281
0.788
0.395
Ruboc_06_G11875
gnl_Prupe_v2.1_Prupe.7G116300.1
gnl_Poptr_v3.0_Potri.006G021000.1
0.860
gnl_Poptr_v3.0_Potri.016G018600.1
0.932
gnl_Manes_v6.1_Manes.S092600.1
0.827
gnl_Manes_v6.1_Manes.08G021600.1
0.814
0.911
gnl_Theca_v1.1_Thecc1EG022462t1
gnl_Carpa_v0.4_evm.model.supercontig_5.71
0.995
gnl_Phavu_v2.1_Phvul.007G222600.1
gnl_Phavu_v2.1_Phvul.001G187100.1
0.705
gnl_Nelnu_v1.0_NNU_023109-RA
0.963
0.937
gnl_Nelnu_v1.0_NNU_002384-RA
0.566
0.919
gnl_Aquco_v3.1_Aqcoe1G456300.1
gnl_Vitvi_Genoscope.12X_GSVIVT01033793001
gnl_Eucgr_v2.0_Eucgr.K02071.1
0.904
gnl_Betvu_v1.2_Bv7_160130_ygjd.t1
0.535
0.991
gnl_Solly_v2.4_Solyc09g009240.1.1
gnl_Solly_v2.4_Solyc10g083560.1.1
0.671
gnl_Mimgu_v2.0_Migut.J00978.1
0.920
gnl_Mimgu_v2.0_Migut.L01761.1
gnl_Ambtr_v1.0_evm_27.model.AmTr_v1.0_scaffold00095.55
0.998
0.896
gnl_Ambtr_v1.0_evm_27.model.AmTr_v1.0_scaffold00040.196
gnl_Arath_TAIR10_AT2G36450.1
gnl_Stras_v2.0_SGA_v2.0_scaffold184T39933
Maldo_HF_25755
Maldo_GDDH_07G1263200
Malsy_07g022270
Maldo_HC_hap2_g45108.t1
Pyrco_BP1_019715.1
Pyrco_BP_DH_07g24220
Pyrbe_050099
Pyrpy_N_07g2604.1
Pyrusco7g020010.1
Pyrco_DAP_Chr1v0.1_01898.1
Pyrco_BP1_012285.1
Pyrpy_N_01g1745.1
Pyrbe_001882
Pyrco_BP_DH_01g20680
Maldo_HC_hap2_g27385.t1
0.981
Maldo_GDDH_01G1196300
Malsy_01g017590
Maldo_HF_23605
Malba_026516
0.998
gnl_Prupe_v2.1_Prupe.2G289600.1
Pruav_sc0001405.1_g520.1.mk
Rubch_LG07.445
0.095
Ruboc_07_G09488
0.997
FraveV4A2_7g28960.t1
0.802
Rosch_v2.0_Chr1g0376651
gnl_Mimgu_v2.0_Migut.F00381.1
gnl_Eucgr_v2.0_Eucgr.A02817.1
gnl_Aquco_v3.1_Aqcoe7G048900.1
0.748
0.877
gnl_Aquco_v3.1_Aqcoe5G426200.1
0.166
gnl_Solly_v2.4_Solyc06g035700.1.1
0.850
gnl_Solly_v2.4_Solyc08g080290.2.1
1.000
gnl_Medtr_Mt4.0v1_Medtr1g101550.1
0.573
gnl_Phavu_v2.1_Phvul.007G066520.1
gnl_Arath_TAIR10_AT1G12630.1
0.864
0.887
gnl_Arath_TAIR10_AT5G52020.1
0.458
gnl_Vitvi_Genoscope.12X_GSVIVT01010629001
gnl_Manes_v6.1_Manes.06G143200.1
0.989
gnl_Manes_v6.1_Manes.14G029500.1
0.329
gnl_Poptr_v3.0_Potri.015G136300.1
0.991
gnl_Poptr_v3.0_Potri.012G134000.1
gnl_Theca_v1.1_Thecc1EG015083t1
gnl_Eucgr_v2.0_Eucgr.K00220.1
gnl_Nelnu_v1.0_NNU_021176-RA
gnl_Nelnu_v1.0_NNU_006826-RA
1.000
Malsy_04g007210
Maldo_GDDH_04G1067700
Maldo_HC_hap2_g34624.t1
Malsi_04g007530
Maldo_Gala_04g006950
Malba_001829
Maldo_HF_01327
Pyrusco4g013840.1
Pyrpy_N_04g0619.1
Pyrbe_038285
Pyrco_BP_DH_04g06130
Pyrco_DAP_Chr4v0.1_08658.1
Pyrco_BP1_011973.1
Pyrbe_045391
Pyrpy_N_06g0652.1
Pyrco_BP1_028454.1
Pyrusco6g007010.1
0.822
Maldo_HC_hap2_g40854.t1
Maldo_HF_08316
Malba_003771
gnl_Prupe_v2.1_Prupe.5G090800.1
Pruav_sc0000355.1_g370.1.mk
gnl_Prupe_v2.1_Prupe.5G090300.1
1.000
Pruav_sc0000107.1_g530.1.br
gnl_Prupe_v2.1_Prupe.5G090400.1
Pruav_sc0000107.1_g550.1.mk
gnl_Prupe_v2.1_Prupe.5G090600.1
Pruav_sc0000355.1_g420.1.mk
0.740
0.426
gnl_Prupe_v2.1_Prupe.5G090700.1
Pruav_sc0000355.1_g390.1.mk
Rubch_LG05.1426
0.994
Ruboc_05_G14850
FraveV4A2_5g01460.t1
Rosch_v2.0_Chr7g0199231
0.988
gnl_Medtr_Mt4.0v1_Medtr4g102670.1
0.993
gnl_Phavu_v2.1_Phvul.003G212700.1
gnl_Phavu_v2.1_Phvul.002G154000.1
gnl_Poptr_v3.0_Potri.003G121200.1
gnl_Poptr_v3.0_Potri.001G110500.1
0.948
gnl_Manes_v6.1_Manes.01G067300.1
0.286
gnl_Carpa_v0.4_evm.model.supercontig_74.86
gnl_Theca_v1.1_Thecc1EG016176t1
0.958
gnl_Mimgu_v2.0_Migut.E01297.1
0.572
gnl_Stras_v2.0_SGA_v2.0_scaffold196T40884
0.878
0.186
gnl_Mimgu_v2.0_Migut.A00921.1
0.995
gnl_Utrgi_v4.1_Scf01154.g23971.t1
0.846
gnl_Stras_v2.0_SGA_v2.0_scaffold286T47100
gnl_Eucgr_v2.0_Eucgr.D01924.1
gnl_Musac_v1_GSMUA_Achr1T03860_001
gnl_Musac_v1_GSMUA_Achr5T10470_001
0.257
gnl_Musac_v1_GSMUA_Achr4T29090_001
0.951
gnl_Musac_v1_GSMUA_Achr11T03210_001
gnl_Elagu_v2.0_p5_sc00016.V1.gene503
0.811
gnl_Orysa_v7.0_LOC_Os04g48330.1
0.940
0.937
gnl_Sorbi_v3.1_Sobic.006G184700.1
gnl_Orysa_v7.0_LOC_Os02g45420.1
0.803
gnl_Sorbi_v3.1_Sobic.004G283300.1
1.000
gnl_Orysa_v7.0_LOC_Os10g38000.1
0.406
gnl_Sorbi_v3.1_Sobic.001G531100.1
0.941
gnl_Elagu_v2.0_p5_sc00053.V1.gene140
gnl_Musac_v1_GSMUA_Achr3T18160_001
gnl_Spipo_v2_Spipo1G0096800
gnl_Ambtr_v1.0_evm_27.model.AmTr_v1.0_scaffold00069.105

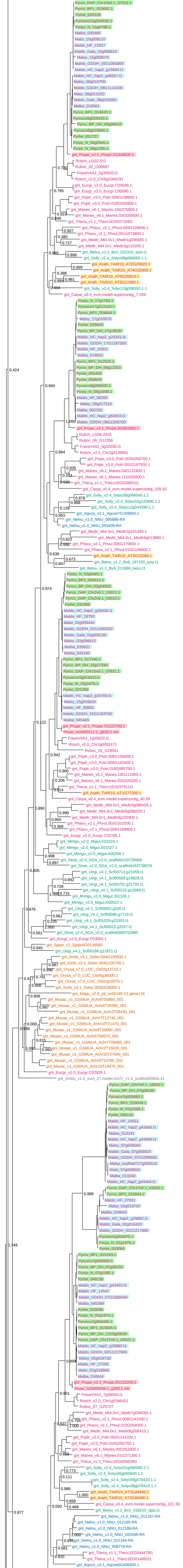

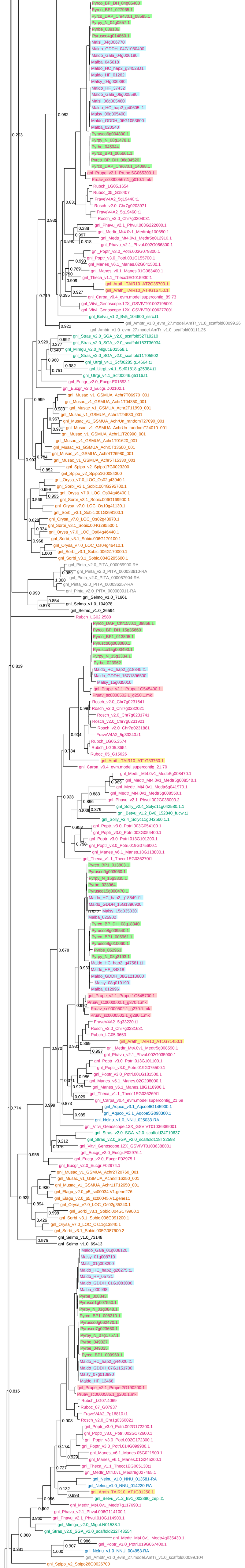

Pyrco_BP_DH_04g05400
Pyrco_BP1_027865.1
Pyrco_DAP_Chr4v0.1_08585.1
Pyrpy_N_04g0557.1
Pyrbe_038186
Pyrusco4g014860.1
Malsi_04g008770
Maldo_GDDH_04G1060400
Maldo_Gala_04g006180
Malba_045616
Maldo_HC_hap2_g34528.t1
Maldo_HF_01262
Malsy_04g006380
Maldo_HF_37432
Maldo_Gala_06g005590
Malsi_06g005460
Maldo_HC_hap2_g40605.t1
Malsy_06g005400
Maldo_GDDH_06G1053600
Malba_020540
Pyrusco6g004800.1
Pyrpy_N_06g0470.1
Pyrbe_045044
Pyrco_BP1_005661.1
Pyrco_BP_DH_06g04520
Pyrco_DAP_Chr6v0.1_14098.1
gnl_Prupe_v2.1_Prupe.5G065300.1
Pruav_sc0000567.1_g010.1.mk
Rubch_LG05.1654
Ruboc_05_G18407
FraveV4A2_5g19440.t1
Rosch_v2.0_Chr7g0203971
FraveV4A2_5g19460.t1
Rosch_v2.0_Chr7g0204031
gnl_Phavu_v2.1_Phvul.003G222600.1
gnl_Medtr_Mt4.0v1_Medtr4g100650.1
gnl_Medtr_Mt4.0v1_Medtr5g012610.1
gnl_Phavu_v2.1_Phvul.002G056800.1
gnl_Poptr_v3.0_Potri.003G079300.1
gnl_Poptr_v3.0_Potri.001G155700.1
gnl_Manes_v6.1_Manes.02G041500.1
gnl_Manes_v6.1_Manes.01G083400.1
gnl_Theca_v1.1_Thecc1EG015930t1
gnl_Arath_TAIR10_AT2G35700.1
gnl_Arath_TAIR10_AT4G16750.1
gnl_Carpa_v0.4_evm.model.supercontig_89.73
gnl_Vitvi_Genoscope.12X_GSVIVT01002195001
gnl_Vitvi_Genoscope.12X_GSVIVT01006277001
gnl_Betvu_v1.2_Bv5_104600_sscr.t1
gnl_Ambtr_v1.0_evm_27.model.AmTr_v1.0_scaffold00099.26
gnl_Ambtr_v1.0_evm_27.model.AmTr_v1.0_scaffold00111.25
gnl_Stras_v2.0_SGA_v2.0_scaffold52T19210
gnl_Stras_v2.0_SGA_v2.0_scaffold153T36934
gnl_Mimgu_v2.0_Migut.B01558.1
gnl_Stras_v2.0_SGA_v2.0_scaffold11T05502
gnl_Utrgi_v4.1_Scf00285.g14664.t1
gnl_Utrgi_v4.1_Scf01828.g25384.t1
gnl_Utrgi_v4.1_Scf00046.g5116.t1
gnl_Eucgr_v2.0_Eucgr.E01593.1
gnl_Eucgr_v2.0_Eucgr.D02102.1
gnl_Musac_v1_GSMUA_Achr7T06970_001
gnl_Musac_v1_GSMUA_Achr1T04350_001
gnl_Musac_v1_GSMUA_Achr2T11990_001
gnl_Musac_v1_GSMUA_Achr4T24580_001
gnl_Musac_v1_GSMUA_AchrUn_randomT27090_001
gnl_Musac_v1_GSMUA_AchrUn_randomT24010_001
gnl_Musac_v1_GSMUA_Achr11T20990_001
gnl_Musac_v1_GSMUA_Achr1T01620_001
gnl_Musac_v1_GSMUA_Achr5T13500_001
gnl_Musac_v1_GSMUA_Achr4T26980_001
gnl_Musac_v1_GSMUA_Achr5T15330_001
gnl_Spipo_v2_Spipo17G0023200
gnl_Spipo_v2_Spipo1G0084300
gnl_Orysa_v7.0_LOC_Os02g43940.1
gnl_Sorbi_v3.1_Sobic.004G295700.1
gnl_Orysa_v7.0_LOC_Os04g46400.1
gnl_Sorbi_v3.1_Sobic.006G169900.1
gnl_Orysa_v7.0_LOC_Os10g41130.1
gnl_Sorbi_v3.1_Sobic.001G298100.1
gnl_Orysa_v7.0_LOC_Os02g43970.1
gnl_Sorbi_v3.1_Sobic.004G295500.1
gnl_Orysa_v7.0_LOC_Os04g46440.1
gnl_Sorbi_v3.1_Sobic.006G170100.1
gnl_Orysa_v7.0_LOC_Os04g46410.1
gnl_Sorbi_v3.1_Sobic.006G170000.1
gnl_Sorbi_v3.1_Sobic.004G295600.1
gnl_Pinta_v2.0_PITA_000069900-RA
gnl_Pinta_v2.0_PITA_000033810-RA
gnl_Pinta_v2.0_PITA_000057904-RA
gnl_Pinta_v2.0_PITA_000036257-RA
gnl_Pinta_v2.0_PITA_000080911-RA
gnl_Selmo_v1.0_71661
gnl_Selmo_v1.0_104978
gnl_Selmo_v1.0_26594
Rubch_LG02.2580
Pyrco_DAP_Chr15v0.1_39868.1
Pyrco_BP_DH_15g35660
Pyrco_BP1_013805.1
Pyrusco0g003080.1
Pyrusco15g000490.1
Pyrpy_N_15g3334.1
Pyrbe_023962
Maldo_HC_hap2_g18845.t1
Maldo_GDDH_15G1396500
Malsy_15g035010
gnl_Prupe_v2.1_Prupe.1G545400.1
Pruav_sc0000502.1_g250.1.mk
Rosch_v2.0_Chr7g0231641
Rosch_v2.0_Chr7g0232021
Rosch_v2.0_Chr7g0231741
Rosch_v2.0_Chr7g0231921
Rosch_v2.0_Chr7g0231881
FraveV4A2_5g33240.t1
Rubch_LG05.3574
Rubch_LG05.3654
Ruboc_05_G15626
gnl_Arath_TAIR10_AT1G33760.1
gnl_Carpa_v0.4_evm.model.supercontig_21.70
gnl_Medtr_Mt4.0v1_Medtr5g008470.1
gnl_Medtr_Mt4.0v1_Medtr5g008540.1
gnl_Medtr_Mt4.0v1_Medtr6g041970.1
gnl_Medtr_Mt4.0v1_Medtr5g008550.1
gnl_Phavu_v2.1_Phvul.002G036000.2
gnl_Solly_v2.4_Solyc11g042580.1.1
gnl_Betvu_v1.2_Bv6_152840_fucw.t1
gnl_Solly_v2.4_Solyc11g042560.1.1
gnl_Poptr_v3.0_Potri.003G054100.1
gnl_Poptr_v3.0_Potri.003G054400.1
gnl_Poptr_v3.0_Potri.013G101200.1
gnl_Poptr_v3.0_Potri.019G075600.1
gnl_Manes_v6.1_Manes.18G118800.1
gnl_Theca_v1.1_Thecc1EG036270t1
Pyrco_BP1_013803.1
Pyrusco0g003060.1
Pyrpy_N_15g3335.1
Pyrbe_023964
Pyrusco15g000470.1
Maldo_HC_hap2_g18849.t1
Maldo_GDDH_15G1396900
Malsy_15g035030
Malba_025802
Pyrco_BP_DH_08g18340
Pyrusco8g009540.1
Pyrco_BP1_005061.1
Pyrusco8g010060.1
Pyrbe_052953
Pyrpy_N_08g2193.1
Maldo_HC_hap2_g47581.t1
Maldo_HF_34818
Maldo_GDDH_08G1213600
Malsy_08g019190
Malba_012996
gnl_Prupe_v2.1_Prupe.1G545700.1
Pruav_sc0000502.1_g370.1.mk
Pruav_sc0000502.1_g270.1.mk
Pruav_sc0000502.1_g280.1.mk
FraveV4A2_5g33220.t1
Rosch_v2.0_Chr7g0231631
Rubch_LG05.3653
gnl_Arath_TAIR10_AT1G71450.1
gnl_Medtr_Mt4.0v1_Medtr5g008590.1
gnl_Phavu_v2.1_Phvul.002G035900.1
gnl_Poptr_v3.0_Potri.013G101100.1
gnl_Poptr_v3.0_Potri.019G075500.1
gnl_Poptr_v3.0_Potri.001G181500.1
gnl_Manes_v6.1_Manes.02G208000.1
gnl_Manes_v6.1_Manes.18G118900.1
gnl_Theca_v1.1_Thecc1EG036269t1
gnl_Carpa_v0.4_evm.model.supercontig_21.69
gnl_Aquco_v3.1_Aqcoe6G145900.1
gnl_Aquco_v3.1_Aqcoe5G098300.1
gnl_Nelnu_v1.0_NNU_025033-RA
gnl_Vitvi_Genoscope.12X_GSVIVT01036389001
gnl_Stras_v2.0_SGA_v2.0_scaffold24T10637
gnl_Stras_v2.0_SGA_v2.0_scaffold118T32598
gnl_Vitvi_Genoscope.12X_GSVIVT01036388001
gnl_Eucgr_v2.0_Eucgr.F02976.1
gnl_Eucgr_v2.0_Eucgr.F02975.1
gnl_Eucgr_v2.0_Eucgr.F02974.1
gnl_Musac_v1_GSMUA_Achr2T20760_001
gnl_Musac_v1_GSMUA_Achr8T16250_001
gnl_Musac_v1_GSMUA_Achr11T12650_001
gnl_Elagu_v2.0_p5_sc00034.V1.gene276
gnl_Elagu_v2.0_p5_sc00045.V1.gene11
gnl_Orysa_v7.0_LOC_Os02g35240.1
gnl_Sorbi_v3.1_Sobic.004G179900.1
gnl_Sorbi_v3.1_Sobic.006G091200.1
gnl_Orysa_v7.0_LOC_Os11g13840.1
gnl_Sorbi_v3.1_Sobic.005G087600.2
gnl_Selmo_v1.0_73148
gnl_Selmo_v1.0_69413
Maldo_Gala_01g008120
Malsy_01g008710
Malsi_01g008200
Maldo_HC_hap2_g26275.t1
Maldo_HF_05721
Maldo_GDDH_01G1083000
Malba_000998
Pyrbe_000843
Pyrusco1g007550.1
Pyrpy_N_01g0848.1
Pyrco_BP1_008210.1
Pyrusco0g062470.1
Pyrusco7g023660.1
Pyrpy_N_07g1757.1
Pyrbe_049027
Pyrbe_049035
Pyrco_BP1_008969.1
Maldo_HC_hap2_g44020.t1
Maldo_GDDH_07G1151700
Malsy_07g013890
Maldo_HF_12468
gnl_Prupe_v2.1_Prupe.2G190200.1
Pruav_sc0000586.1_g200.1.mk
Rubch_LG07.4069
Ruboc_07_G07937
FraveV4A2_7g16810.t1
Rosch_v2.0_Chr1g0360021
gnl_Poptr_v3.0_Potri.002G172200.1
gnl_Poptr_v3.0_Potri.002G172600.1
gnl_Poptr_v3.0_Potri.002G172300.1
gnl_Poptr_v3.0_Potri.014G099900.1
gnl_Manes_v6.1_Manes.05G021900.1
gnl_Manes_v6.1_Manes.01G245200.1
gnl_Theca_v1.1_Thecc1EG005130t1
gnl_Medtr_Mt4.0v1_Medtr8g027465.1
gnl_Nelnu_v1.0_NNU_013581-RA
gnl_Nelnu_v1.0_NNU_014220-RA
gnl_Arath_TAIR10_AT1G01250.1
gnl_Betvu_v1.2_Bv1_002890_zepi.t1
gnl_Medtr_Mt4.0v1_Medtr7g117690.1
gnl_Phavu_v2.1_Phvul.006G114100.1
gnl_Phavu_v2.1_Phvul.010G114800.1
gnl_Mimgu_v2.0_Migut.N01538.1
gnl_Stras_v2.0_SGA_v2.0_scaffold232T43554
gnl_Medtr_Mt4.0v1_Medtr4g035430.1
gnl_Poptr_v3.0_Potri.019G067400.1
gnl_Nelnu_v1.0_NNU_004953-RA
gnl_Ambtr_v1.0_evm_27.model.AmTr_v1.0_scaffold00099.104
gnl_Spipo_v2_Spipo26G0026700

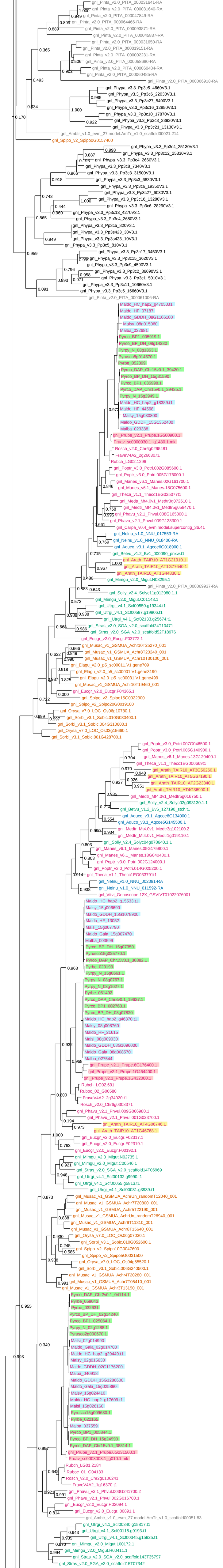

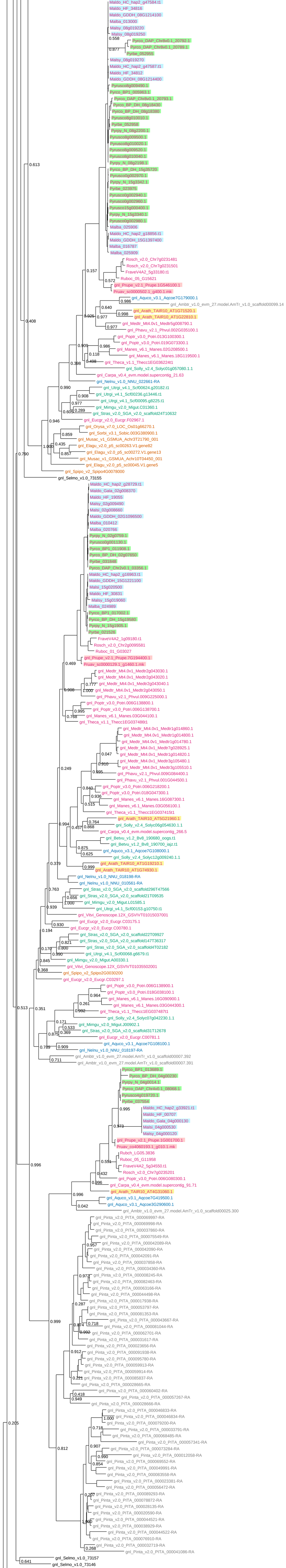
Maldo_HC_hap2_g47584.t1
Maldo_HF_34816
Maldo_GDDH_08G1214100
Malba_013000
Malsy_08g019220
Malsy_08g019250
Pyrco_DAP_Chr8v0.1_20792.1
Pyrco_DAP_Chr8v0.1_20789.1
Pyrbe_052955
Malsy_08g019270
Maldo_HC_hap2_g47587.t1
Maldo_HF_34812
Maldo_GDDH_08G1214400
Pyrusco8g009490.1
Pyrco_BP1_005963.1
Pyrco_DAP_Chr8v0.1_20793.1
Pyrco_BP_DH_08g18430
Pyrco_BP_DH_08g18380
Pyrusco8g010010.1
Pyrbe_052958
Pyrpy_N_08g2200.1
Pyrusco8g009500.1
Pyrusco8g010020.1
Pyrusco8g009520.1
Pyrusco8g010040.1
Pyrpy_N_08g2198.1
Pyrco_BP_DH_15g35720
Pyrusco0g002970.1
Pyrpy_N_15g3342.1
Pyrbe_023975
Pyrusco0g002940.1
Pyrusco0g002960.1
Pyrusco15g000400.1
Pyrpy_N_15g3340.1
Pyrusco0g002980.1
Malba_025906
Maldo_HC_hap2_g18856.t1
Maldo_GDDH_15G1397400
Malba_016787
Malba_025909
Rosch_v2.0_Chr7g0231481
Rosch_v2.0_Chr7g0231501
FraveV4A2_5g33180.t1
Ruboc_05_G15621
gnl_Prupe_v2.1_Prupe.1G546100.1
Pruav_sc0000502.1_g400.1.mk
gnl_Aquco_v3.1_Aqcoe7G179000.1
gnl_Ambtr_v1.0_evm_27.model.AmTr_v1.0_scaffold00099.14
gnl_Arath_TAIR10_AT1G71520.1
gnl_Arath_TAIR10_AT1G22810.1
gnl_Medtr_Mt4.0v1_Medtr5g008790.1
gnl_Phavu_v2.1_Phvul.002G035100.1
gnl_Poptr_v3.0_Potri.013G100300.1
gnl_Poptr_v3.0_Potri.019G073300.1
gnl_Manes_v6.1_Manes.02G208500.1
gnl_Manes_v6.1_Manes.18G119500.1
gnl_Theca_v1.1_Thecc1EG036224t1
gnl_Solly_v2.4_Solyc01g057080.1.1
gnl_Carpa_v0.4_evm.model.supercontig_21.63
gnl_Nelnu_v1.0_NNU_022661-RA
gnl_Utrgi_v4.1_Scf00624.g20182.t1
gnl_Utrgi_v4.1_Scf00236.g13446.t1
gnl_Utrgi_v4.1_Scf00095.g8225.t1
gnl_Mimgu_v2.0_Migut.C01360.1
gnl_Stras_v2.0_SGA_v2.0_scaffold24T10632
gnl_Eucgr_v2.0_Eucgr.F02967.1
gnl_Orysa_v7.0_LOC_Os01g66270.1
gnl_Sorbi_v3.1_Sobic.003G380900.1
gnl_Musac_v1_GSMUA_Achr3T21790_001
gnl_Elagu_v2.0_p5_sc00263.V1.gene82
gnl_Elagu_v2.0_p5_sc00272.V1.gene13
gnl_Musac_v1_GSMUA_Achr10T04450_001
gnl_Elagu_v2.0_p5_sc00045.V1.gene5
gnl_Spipo_v2_Spipo4G0078000
gnl_Selmo_v1.0_73155
Maldo_HC_hap2_g28729.t1
Maldo_Gala_02g008370
Maldo_HF_19055
Malsy_02g009490
Malsi_02g008660
Maldo_GDDH_02G1096500
Malba_010412
Malba_020766
Pyrpy_N_02g0759.1
Pyrusco0g001130.1
Pyrco_BP1_011908.1
Pyrco_BP_DH_02g07650
Pyrbe_031848
Pyrco_DAP_Chr2v0.1_03356.1
Maldo_HC_hap2_g16963.t1
Maldo_GDDH_15G1221100
Malsi_15g020500
Maldo_HF_30831
Malsy_15g019060
Malba_024989
Pyrco_BP1_017002.1
Pyrco_BP_DH_15g19580
Pyrpy_N_15g1905.1
Pyrbe_021526
FraveV4A2_1g09180.t1
Rosch_v2.0_Chr2g0095581
Ruboc_01_G03027
gnl_Prupe_v2.1_Prupe.7G194400.1
Pruav_sc0000129.1_g1460.1.mk
gnl_Medtr_Mt4.0v1_Medtr2g043030.1
gnl_Medtr_Mt4.0v1_Medtr2g043020.1
gnl_Medtr_Mt4.0v1_Medtr2g043040.1
gnl_Medtr_Mt4.0v1_Medtr2g043050.1
gnl_Phavu_v2.1_Phvul.009G225000.1
gnl_Poptr_v3.0_Potri.006G138800.1
gnl_Poptr_v3.0_Potri.006G138700.1
gnl_Manes_v6.1_Manes.03G044100.1
gnl_Theca_v1.1_Thecc1EG037486t1
gnl_Medtr_Mt4.0v1_Medtr1g014860.1
gnl_Medtr_Mt4.0v1_Medtr1g014800.1
gnl_Medtr_Mt4.0v1_Medtr1g014780.1
gnl_Medtr_Mt4.0v1_Medtr7g028925.1
gnl_Medtr_Mt4.0v1_Medtr1g014820.1
gnl_Medtr_Mt4.0v1_Medtr3g105480.1
gnl_Medtr_Mt4.0v1_Medtr3g105510.1
gnl_Phavu_v2.1_Phvul.009G084400.1
gnl_Phavu_v2.1_Phvul.001G044500.1
gnl_Poptr_v3.0_Potri.006G218200.1
gnl_Poptr_v3.0_Potri.018G047300.1
gnl_Manes_v6.1_Manes.16G087300.1
gnl_Manes_v6.1_Manes.03G056100.1
gnl_Theca_v1.1_Thecc1EG037415t1
gnl_Arath_TAIR10_AT5G21960.1
gnl_Solly_v2.4_Solyc06g054630.1.1
gnl_Carpa_v0.4_evm.model.supercontig_266.5
gnl_Betvu_v1.2_Bv8_190680_oogs.t1
gnl_Betvu_v1.2_Bv8_190700_iajz.t1
gnl_Aquco_v3.1_Aqcoe7G108000.1
gnl_Solly_v2.4_Solyc12g009240.1.1
gnl_Arath_TAIR10_AT1G19210.1
gnl_Arath_TAIR10_AT1G74930.1
gnl_Nelnu_v1.0_NNU_018198-RA
gnl_Nelnu_v1.0_NNU_010561-RA
gnl_Stras_v2.0_SGA_v2.0_scaffold296T47566
gnl_Stras_v2.0_SGA_v2.0_scaffold21T09535
gnl_Mimgu_v2.0_Migut.L01585.1
gnl_Utrgi_v4.1_Scf00153.g10750.t1
gnl_Vitvi_Genoscope.12X_GSVIVT01015037001
gnl_Eucgr_v2.0_Eucgr.C03175.1
gnl_Eucgr_v2.0_Eucgr.C00780.1
gnl_Stras_v2.0_SGA_v2.0_scaffold22T09927
gnl_Stras_v2.0_SGA_v2.0_scaffold147T36317
gnl_Stras_v2.0_SGA_v2.0_scaffold4T02182
gnl_Utrgi_v4.1_Scf00068.g6679.t1
gnl_Mimgu_v2.0_Migut.A00330.1
gnl_Vitvi_Genoscope.12X_GSVIVT01035502001
gnl_Spipo_v2_Spipo2G0030200
gnl_Eucgr_v2.0_Eucgr.C03297.1
gnl_Poptr_v3.0_Potri.006G138900.1
gnl_Poptr_v3.0_Potri.018G038100.1
gnl_Manes_v6.1_Manes.16G090900.1
gnl_Manes_v6.1_Manes.03G044300.1
gnl_Theca_v1.1_Thecc1EG037487t1
gnl_Solly_v2.4_Solyc07g042230.1.1
gnl_Mimgu_v2.0_Migut.J00902.1
gnl_Stras_v2.0_SGA_v2.0_scaffold31T12678
gnl_Eucgr_v2.0_Eucgr.C00781.1
gnl_Aquco_v3.1_Aqcoe7G108100.1
gnl_Nelnu_v1.0_NNU_018197-RA
gnl_Ambtr_v1.0_evm_27.model.AmTr_v1.0_scaffold00007.392
gnl_Ambtr_v1.0_evm_27.model.AmTr_v1.0_scaffold00007.391
Pyrco_BP1_013689.1
Pyrco_BP_DH_04g00230
Pyrpy_N_04g0014.1
Pyrco_DAP_Chr4v0.1_08068.1
Pyrusco4g019720.1
Pyrbe_037554
Maldo_HC_hap2_g33921.t1
Maldo_HF_00707
Maldo_Gala_04g000130
Malsi_04g000530
Malsy_04g000120
gnl_Prupe_v2.1_Prupe.1G001700.1
Pruav_co4060193.1_g010.1.mk
Rubch_LG05.3836
Ruboc_05_G11958
FraveV4A2_5g34550.t1
Rosch_v2.0_Chr7g0235201
gnl_Poptr_v3.0_Potri.006G080300.1
gnl_Carpa_v0.4_evm.model.supercontig_91.71
gnl_Arath_TAIR10_AT4G31060.1
gnl_Aquco_v3.1_Aqcoe7G419500.1
gnl_Aquco_v3.1_Aqcoe3G290600.1
gnl_Ambtr_v1.0_evm_27.model.AmTr_v1.0_scaffold00025.300
gnl_Pinta_v2.0_PITA_000069997-RA
gnl_Pinta_v2.0_PITA_000069998-RA
gnl_Pinta_v2.0_PITA_000037860-RA
gnl_Pinta_v2.0_PITA_000075549-RA
gnl_Pinta_v2.0_PITA_000042089-RA
gnl_Pinta_v2.0_PITA_000042090-RA
gnl_Pinta_v2.0_PITA_000042091-RA
gnl_Pinta_v2.0_PITA_000037858-RA
gnl_Pinta_v2.0_PITA_000034360-RA
gnl_Pinta_v2.0_PITA_000008245-RA
gnl_Pinta_v2.0_PITA_000082463-RA
gnl_Pinta_v2.0_PITA_000063166-RA
gnl_Pinta_v2.0_PITA_000044498-RA
gnl_Pinta_v2.0_PITA_000017938-RA
gnl_Pinta_v2.0_PITA_000053797-RA
gnl_Pinta_v2.0_PITA_000081353-RA
gnl_Pinta_v2.0_PITA_000043667-RA
gnl_Pinta_v2.0_PITA_000081044-RA
gnl_Pinta_v2.0_PITA_000062701-RA
gnl_Pinta_v2.0_PITA_000031617-RA
gnl_Pinta_v2.0_PITA_000023656-RA
gnl_Pinta_v2.0_PITA_000091938-RA
gnl_Pinta_v2.0_PITA_000095780-RA
gnl_Pinta_v2.0_PITA_000059913-RA
gnl_Pinta_v2.0_PITA_000059914-RA
gnl_Pinta_v2.0_PITA_000085837-RA
gnl_Pinta_v2.0_PITA_000028665-RA
gnl_Pinta_v2.0_PITA_000060402-RA
gnl_Pinta_v2.0_PITA_000057267-RA
gnl_Pinta_v2.0_PITA_000028666-RA
gnl_Pinta_v2.0_PITA_000046833-RA
gnl_Pinta_v2.0_PITA_000046834-RA
gnl_Pinta_v2.0_PITA_000079200-RA
gnl_Pinta_v2.0_PITA_000033791-RA
gnl_Pinta_v2.0_PITA_000068485-RA
gnl_Pinta_v2.0_PITA_000057341-RA
gnl_Pinta_v2.0_PITA_000073284-RA
gnl_Pinta_v2.0_PITA_000012058-RA
gnl_Pinta_v2.0_PITA_000069552-RA
gnl_Pinta_v2.0_PITA_000049991-RA
gnl_Pinta_v2.0_PITA_000083558-RA
gnl_Pinta_v2.0_PITA_000023381-RA
gnl_Pinta_v2.0_PITA_000056472-RA
gnl_Pinta_v2.0_PITA_000089293-RA
gnl_Pinta_v2.0_PITA_000078872-RA
gnl_Pinta_v2.0_PITA_000028135-RA
gnl_Pinta_v2.0_PITA_000020590-RA
gnl_Pinta_v2.0_PITA_000044521-RA
gnl_Pinta_v2.0_PITA_000038929-RA
gnl_Pinta_v2.0_PITA_000044522-RA
gnl_Pinta_v2.0_PITA_000076910-RA
gnl_Pinta_v2.0_PITA_000032719-RA
gnl_Pinta_v2.0_PITA_000041086-RA
gnl_Selmo_v1.0_73157
gnl_Selmo_v1.0_73146

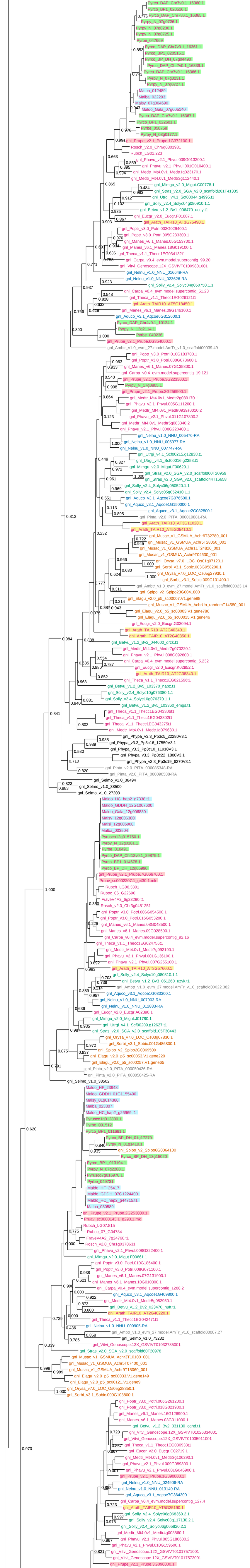

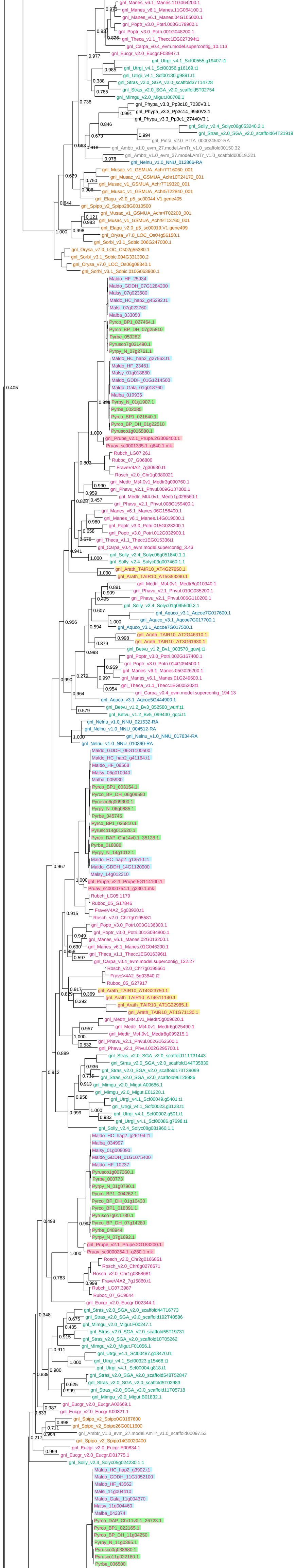
gnl_Manes_v6.1_Manes.11G064200.1
gnl_Manes_v6.1_Manes.11G064100.1
gnl_Manes_v6.1_Manes.04G105000.1
gnl_Poptr_v3.0_Potri.003G179900.1
gnl_Poptr_v3.0_Potri.001G048200.1
gnl_Theca_v1.1_Thecc1EG027394t1
gnl_Carpa_v0.4_evm.model.supercontig_10.113
gnl_Eucgr_v2.0_Eucgr.F03947.1
gnl_Utrgi_v4.1_Scf00555.g19407.t1
gnl_Utrgi_v4.1_Scf00356.g16169.t1
gnl_Utrgi_v4.1_Scf00130.g9891.t1
gnl_Stras_v2.0_SGA_v2.0_scaffold37T14728
gnl_Stras_v2.0_SGA_v2.0_scaffold5T02754
gnl_Mimgu_v2.0_Migut.I00708.1
gnl_Phypa_v3.3_Pp3c10_7030V3.1
gnl_Phypa_v3.3_Pp3c14_9940V3.1
gnl_Phypa_v3.3_Pp3c1_27440V3.1
gnl_Solly_v2.4_Solyc06g053240.2.1
gnl_Stras_v2.0_SGA_v2.0_scaffold64T21919
gnl_Pinta_v2.0_PITA_000024542-RA
gnl_Ambtr_v1.0_evm_27.model.AmTr_v1.0_scaffold00150.32
gnl_Ambtr_v1.0_evm_27.model.AmTr_v1.0_scaffold00019.321
gnl_Nelnu_v1.0_NNU_012866-RA
gnl_Musac_v1_GSMUA_Achr7T16060_001
gnl_Musac_v1_GSMUA_Achr10T24170_001
gnl_Musac_v1_GSMUA_Achr7T19320_001
gnl_Musac_v1_GSMUA_Achr5T22840_001
gnl_Elagu_v2.0_p5_sc00044.V1.gene405
gnl_Spipo_v2_Spipo28G0010500
gnl_Musac_v1_GSMUA_Achr4T02200_001
gnl_Musac_v1_GSMUA_Achr9T13760_001
gnl_Elagu_v2.0_p5_sc00019.V1.gene499
gnl_Orysa_v7.0_LOC_Os04g56150.1
gnl_Sorbi_v3.1_Sobic.006G247000.1
gnl_Orysa_v7.0_LOC_Os02g55380.1
gnl_Sorbi_v3.1_Sobic.004G331300.2
gnl_Orysa_v7.0_LOC_Os06g08340.1
gnl_Sorbi_v3.1_Sobic.010G063900.1
Maldo_HF_25934
Maldo_GDDH_07G1284200
Malsy_07g023680
Maldo_HC_hap2_g45292.t1
Malsi_07g022760
Malba_033050
Pyrco_BP1_027464.1
Pyrco_BP_DH_07g25810
Pyrbe_050262
Pyrusco7g021460.1
Pyrpy_N_07g2761.1
Maldo_HC_hap2_g27563.t1
Maldo_HF_23461
Malsy_01g018880
Maldo_GDDH_01G1214500
Maldo_Gala_01g018760
Malba_019935
Pyrpy_N_01g1907.1
Pyrbe_020085
Pyrco_BP1_021640.1
Pyrco_BP_DH_01g22510
Pyrusco1g016580.1
gnl_Prupe_v2.1_Prupe.2G306400.1
Pruav_sc0001335.1_g640.1.mk
Rubch_LG07.261
Ruboc_07_G06800
FraveV4A2_7g30930.t1
Rosch_v2.0_Chr1g0380021
gnl_Medtr_Mt4.0v1_Medtr3g090760.1
gnl_Phavu_v2.1_Phvul.009G137000.1
gnl_Medtr_Mt4.0v1_Medtr1g028560.1
gnl_Phavu_v2.1_Phvul.008G159400.1
gnl_Manes_v6.1_Manes.06G156400.1
gnl_Manes_v6.1_Manes.14G019000.1
gnl_Poptr_v3.0_Potri.015G023200.1
gnl_Poptr_v3.0_Potri.012G032900.1
gnl_Theca_v1.1_Thecc1EG015336t1
gnl_Carpa_v0.4_evm.model.supercontig_3.43
gnl_Solly_v2.4_Solyc06g051840.1.1
gnl_Solly_v2.4_Solyc03g007460.1.1
gnl_Arath_TAIR10_AT4G27950.1
gnl_Arath_TAIR10_AT5G53290.1
gnl_Medtr_Mt4.0v1_Medtr8g010340.1
gnl_Phavu_v2.1_Phvul.010G035200.1
gnl_Phavu_v2.1_Phvul.008G110200.1
gnl_Solly_v2.4_Solyc01g095500.2.1
gnl_Aquco_v3.1_Aqcoe7G017600.1
gnl_Aquco_v3.1_Aqcoe7G017700.1
gnl_Aquco_v3.1_Aqcoe7G017500.1
gnl_Arath_TAIR10_AT2G46310.1
gnl_Arath_TAIR10_AT3G61630.1
gnl_Betvu_v1.2_Bv1_003570_quwj.t1
gnl_Poptr_v3.0_Potri.002G167400.1
gnl_Poptr_v3.0_Potri.014G094500.1
gnl_Manes_v6.1_Manes.05G026200.1
gnl_Manes_v6.1_Manes.01G249600.1
gnl_Theca_v1.1_Thecc1EG005203t1
gnl_Carpa_v0.4_evm.model.supercontig_194.13
gnl_Aquco_v3.1_Aqcoe5G444900.1
gnl_Betvu_v1.2_Bv3_052580_wurf.t1
gnl_Betvu_v1.2_Bv5_099430_qqci.t1
gnl_Nelnu_v1.0_NNU_021532-RA
gnl_Nelnu_v1.0_NNU_004512-RA
gnl_Nelnu_v1.0_NNU_017634-RA
gnl_Nelnu_v1.0_NNU_010390-RA
Maldo_GDDH_06G1100500
Maldo_HC_hap2_g41164.t1
Maldo_HF_08568
Malsy_06g010040
Malba_005930
Pyrco_BP1_003154.1
Pyrco_BP_DH_06g09580
Pyrusco6g009300.1
Pyrpy_N_06g0885.1
Pyrbe_045740
Pyrco_BP1_026810.1
Pyrusco14g012520.1
Pyrco_DAP_Chr14v0.1_35128.1
Pyrbe_018088
Pyrpy_N_14g1012.1
Maldo_HC_hap2_g13510.t1
Maldo_GDDH_14G1120000
Malsy_14g012310
gnl_Prupe_v2.1_Prupe.5G114100.1
Pruav_sc0000754.1_g230.1.mk
Rubch_LG05.1179
Ruboc_05_G17846
FraveV4A2_5g03920.t1
Rosch_v2.0_Chr7g0195581
gnl_Poptr_v3.0_Potri.003G136300.1
gnl_Poptr_v3.0_Potri.001G094800.1
gnl_Manes_v6.1_Manes.02G013200.1
gnl_Manes_v6.1_Manes.01G046200.1
gnl_Theca_v1.1_Thecc1EG016356t1
gnl_Carpa_v0.4_evm.model.supercontig_122.27
Rosch_v2.0_Chr7g0195661
FraveV4A2_5g03840.t2
Ruboc_05_G27917
gnl_Arath_TAIR10_AT4G23750.1
gnl_Arath_TAIR10_AT4G11140.1
gnl_Arath_TAIR10_AT1G22985.1
gnl_Arath_TAIR10_AT1G71130.1
gnl_Medtr_Mt4.0v1_Medtr5g009620.1
gnl_Medtr_Mt4.0v1_Medtr6g025490.1
gnl_Medtr_Mt4.0v1_Medtr8g099215.1
gnl_Phavu_v2.1_Phvul.002G162500.1
gnl_Phavu_v2.1_Phvul.002G295700.1
gnl_Stras_v2.0_SGA_v2.0_scaffold111T31443
gnl_Stras_v2.0_SGA_v2.0_scaffold144T35839
gnl_Stras_v2.0_SGA_v2.0_scaffold173T39099
gnl_Stras_v2.0_SGA_v2.0_scaffold96T28986
gnl_Mimgu_v2.0_Migut.A00686.1
gnl_Mimgu_v2.0_Migut.E01228.1
gnl_Utrgi_v4.1_Scf00049.g5401.t1
gnl_Utrgi_v4.1_Scf00023.g3128.t1
gnl_Utrgi_v4.1_Scf00002.g501.t1
gnl_Utrgi_v4.1_Scf00086.g7698.t1
gnl_Solly_v2.4_Solyc08g081960.1.1
Maldo_HC_hap2_g26194.t1
Malba_034997
Malsy_01g008090
Maldo_GDDH_01G1075400
Maldo_HF_10237
Pyrusco1g007360.1
Pyrbe_000773
Pyrpy_N_01g0780.1
Pyrco_BP1_004262.1
Pyrco_BP_DH_01g10430
Pyrco_BP1_018391.1
Pyrusco7g011780.1
Pyrco_BP_DH_07g14280
Pyrbe_048944
Pyrpy_N_07g1692.1
gnl_Prupe_v2.1_Prupe.2G183200.1
Pruav_sc0000254.1_g260.1.mk
Rosch_v2.0_Chr2g0166851
Rosch_v2.0_Chr6g0276671
Rosch_v2.0_Chr1g0358681
FraveV4A2_7g15860.t1
Rubch_LG07.3987
Ruboc_07_G19644
gnl_Eucgr_v2.0_Eucgr.D02344.1
gnl_Stras_v2.0_SGA_v2.0_scaffold44T16773
gnl_Stras_v2.0_SGA_v2.0_scaffold192T40586
gnl_Mimgu_v2.0_Migut.F00247.1
gnl_Stras_v2.0_SGA_v2.0_scaffold55T19731
gnl_Stras_v2.0_SGA_v2.0_scaffold10T05262
gnl_Mimgu_v2.0_Migut.F01056.1
gnl_Utrgi_v4.1_Scf00487.g18470.t1
gnl_Utrgi_v4.1_Scf00323.g15468.t1
gnl_Utrgi_v4.1_Scf00004.g818.t1
gnl_Stras_v2.0_SGA_v2.0_scaffold548T52847
gnl_Stras_v2.0_SGA_v2.0_scaffold5T02983
gnl_Stras_v2.0_SGA_v2.0_scaffold11T05718
gnl_Mimgu_v2.0_Migut.B01832.1
gnl_Eucgr_v2.0_Eucgr.A02669.1
gnl_Eucgr_v2.0_Eucgr.K00321.1
gnl_Spipo_v2_Spipo0G0167600
gnl_Spipo_v2_Spipo26G0011600
gnl_Ambtr_v1.0_evm_27.model.AmTr_v1.0_scaffold00097.53
gnl_Spipo_v2_Spipo14G0020400
gnl_Eucgr_v2.0_Eucgr.E00834.1
gnl_Eucgr_v2.0_Eucgr.D01775.1
gnl_Solly_v2.4_Solyc05g024230.1.1
Maldo_HC_hap2_g3902.t1
Maldo_GDDH_11G1052100
Maldo_HF_43562
Malsi_11g004410
Maldo_Gala_11g004370
Malsy_11g004460
Malba_042374
Pyrco_DAP_Chr11v0.1_26723.1
Pyrco_BP1_022165.1
Pyrco_BP_DH_11g04250
Pyrpy_N_11g0395.1
Pyrusco0g038680.1
Pyrusco11g022180.1
Pyrbe_006500

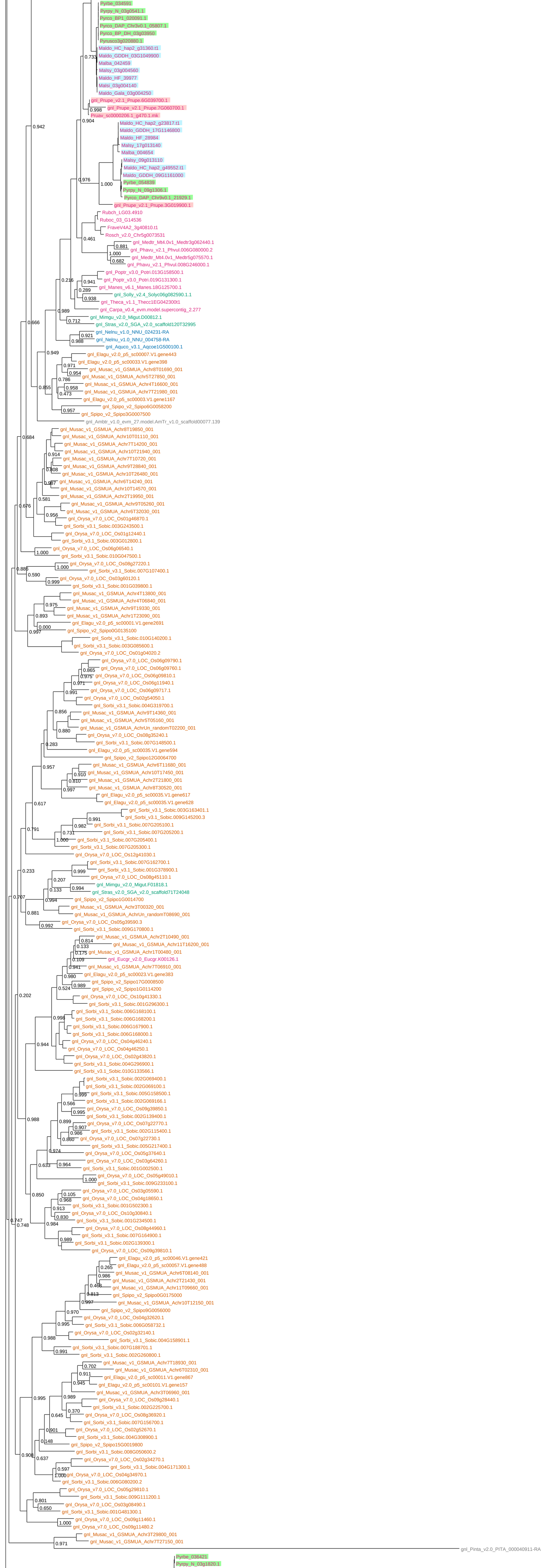
Pyrbe_034591
Pyrpy_N_03g0541.1
Pyrco_BP1_020091.1
Pyrco_DAP_Chr3v0.1_05807.1
Pyrco_BP_DH_03g03950
Pyrus03g020880.1
Maldo_HC_hap2_g31360.t1
Maldo_GDDH_03G1049900
Malba_042459
Malsy_03g004560
Maldo_HF_39977
Malsi_03g004140
Maldo_Gala_03g004250
gnl_Prupe_v2.1_Prupe.6G039700.1
gnl_Prupe_v2.1_Prupe.7G060700.1
Prsav_sc0000206.1_g470.1.mk
Maldo_HC_hap2_g23817.t1
Maldo_GDDH_17G1146800
Maldo_HF_28984
Malsy_17g013140
Malba_004654
Malsy_09g013110
Maldo_HC_hap2_g49552.t1
Maldo_GDDH_09G1161000
Pyrbe_054839
Pyrpy_N_09g1306.1
Pyrco_DAP_Chr9v0.1_21929.1
gnl_Prupe_v2.1_Prupe.3G019900.1
Rubch_LG03.4910
Ruboc_03_G14536
FraveV4A2_3g40810.t1
Rosch_v2.0_Chr5g0073531
gnl_Medtr_Mt4.0v1_Medtr3g062440.1
gnl_Phavu_v2.1_Phvul.006G080000.2
gnl_Medtr_Mt4.0v1_Medtr5g075570.1
gnl_Phavu_v2.1_Phvul.008G246000.1
gnl_Poptr_v3.0_Potri.013G158500.1
gnl_Poptr_v3.0_Potri.019G131300.1
gnl_Manes_v6.1_Manes.18G125700.1
gnl_Solly_v2.4_Solyc06g082590.1.1
gnl_Theca_v1.1_Thecc1EG042300t1
gnl_Carpa_v0.4_evm.model.supercontig_2.277
gnl_Mimgu_v2.0_Migut.D00812.1
gnl_Stras_v2.0_SGA_v2.0_scaffold120T32995
gnl_Nelnu_v1.0_NNU_024231-RA
gnl_Nelnu_v1.0_NNU_004758-RA
gnl_Aquco_v3.1_Aqcoe1G500100.1
gnl_Elagu_v2.0_p5_sc00007.V1.gene443
gnl_Elagu_v2.0_p5_sc00033.V1.gene398
gnl_Musac_v1_GSMUA_Achr8T01690_001
gnl_Musac_v1_GSMUA_Achr5T27850_001
gnl_Musac_v1_GSMUA_Achr4T16600_001
gnl_Musac_v1_GSMUA_Achr7T21980_001
gnl_Elagu_v2.0_p5_sc00003.V1.gene1167
gnl_Spipo_v2_Spipo6G0058200
gnl_Spipo_v2_Spipo3G0007500
gnl_Ambtr_v1.0_evm_27.model.AmTr_v1.0_scaffold00077.139
gnl_Musac_v1_GSMUA_Achr9T19850_001
gnl_Musac_v1_GSMUA_Achr10T01110_001
gnl_Musac_v1_GSMUA_Achr7T14200_001
gnl_Musac_v1_GSMUA_Achr10T21940_001
gnl_Musac_v1_GSMUA_Achr7T10720_001
gnl_Musac_v1_GSMUA_Achr9T28840_001
gnl_Musac_v1_GSMUA_Achr10T26480_001
gnl_Musac_v1_GSMUA_Achr6T14240_001
gnl_Musac_v1_GSMUA_Achr10T14570_001
gnl_Musac_v1_GSMUA_Achr2T19950_001
gnl_Musac_v1_GSMUA_Achr9T05260_001
gnl_Musac_v1_GSMUA_Achr6T32030_001
gnl_Orysa_v7.0_LOC_Os01g46870.1
gnl_Sorbi_v3.1_Sobic.003G243500.1
gnl_Orysa_v7.0_LOC_Os01g12440.1
gnl_Sorbi_v3.1_Sobic.003G012800.1
gnl_Orysa_v7.0_LOC_Os06g06540.1
gnl_Sorbi_v3.1_Sobic.010G047500.1
gnl_Orysa_v7.0_LOC_Os08g27220.1
gnl_Sorbi_v3.1_Sobic.007G107400.1
gnl_Orysa_v7.0_LOC_Os03g60120.1
gnl_Sorbi_v3.1_Sobic.001G039800.1
gnl_Musac_v1_GSMUA_Achr4T13800_001
gnl_Musac_v1_GSMUA_Achr4T06840_001
gnl_Musac_v1_GSMUA_Achr9T19330_001
gnl_Musac_v1_GSMUA_Achr1T23090_001
gnl_Elagu_v2.0_p5_sc00001.V1.gene2691
gnl_Spipo_v2_Spipo0G0135100
gnl_Sorbi_v3.1_Sobic.010G140200.1
gnl_Sorbi_v3.1_Sobic.003G085600.1
gnl_Orysa_v7.0_LOC_Os01g04020.2
gnl_Orysa_v7.0_LOC_Os06g09790.1
gnl_Orysa_v7.0_LOC_Os06g09760.1
gnl_Orysa_v7.0_LOC_Os06g09810.1
gnl_Orysa_v7.0_LOC_Os06g11940.1
gnl_Orysa_v7.0_LOC_Os06g09717.1
gnl_Orysa_v7.0_LOC_Os02g54050.1
gnl_Sorbi_v3.1_Sobic.004G319700.1
gnl_Musac_v1_GSMUA_Achr9T14360_001
gnl_Musac_v1_GSMUA_Achr5T05160_001
gnl_Musac_v1_GSMUA_AchrUn_randomT02200_001
gnl_Orysa_v7.0_LOC_Os08g35240.1
gnl_Sorbi_v3.1_Sobic.007G148500.1
gnl_Elagu_v2.0_p5_sc00035.V1.gene594
gnl_Spipo_v2_Spipo12G0064700
gnl_Musac_v1_GSMUA_Achr6T11680_001
gnl_Musac_v1_GSMUA_Achr10T17450_001
gnl_Musac_v1_GSMUA_Achr2T21800_001
gnl_Musac_v1_GSMUA_Achr8T30520_001
gnl_Elagu_v2.0_p5_sc00035.V1.gene617
gnl_Elagu_v2.0_p5_sc00035.V1.gene628
gnl_Sorbi_v3.1_Sobic.003G163401.1
gnl_Sorbi_v3.1_Sobic.009G145200.3
gnl_Sorbi_v3.1_Sobic.007G205100.1
gnl_Sorbi_v3.1_Sobic.007G205200.1
gnl_Sorbi_v3.1_Sobic.007G205400.1
gnl_Sorbi_v3.1_Sobic.007G205300.1
gnl_Orysa_v7.0_LOC_Os12g41030.1
gnl_Sorbi_v3.1_Sobic.007G162700.1
gnl_Sorbi_v3.1_Sobic.001G378900.1
gnl_Orysa_v7.0_LOC_Os08g45110.1
gnl_Mimgu_v2.0_Migut.F01818.1
gnl_Stras_v2.0_SGA_v2.0_scaffold71T24048
gnl_Spipo_v2_Spipo1G0014700
gnl_Musac_v1_GSMUA_Achr3T00320_001
gnl_Musac_v1_GSMUA_AchrUn_randomT08690_001
gnl_Orysa_v7.0_LOC_Os05g39590.3
gnl_Sorbi_v3.1_Sobic.009G170800.1
gnl_Musac_v1_GSMUA_Achr2T19490_001
gnl_Musac_v1_GSMUA_Achr11T16200_001
gnl_Musac_v1_GSMUA_Achr1T00480_001
gnl_Eucgr_v2.0_Eucgr.K00126.1
gnl_Musac_v1_GSMUA_Achr7T06910_001
gnl_Elagu_v2.0_p5_sc00023.V1.gene383
gnl_Spipo_v2_Spipo17G0008500
gnl_Spipo_v2_Spipo1G0114200
gnl_Orysa_v7.0_LOC_Os10g41330.1
gnl_Sorbi_v3.1_Sobic.001G296300.1
gnl_Sorbi_v3.1_Sobic.006G168100.1
gnl_Sorbi_v3.1_Sobic.006G168200.1
gnl_Sorbi_v3.1_Sobic.006G167900.1
gnl_Sorbi_v3.1_Sobic.006G168000.1
gnl_Orysa_v7.0_LOC_Os04g46240.1
gnl_Orysa_v7.0_LOC_Os04g46250.1
gnl_Orysa_v7.0_LOC_Os02g43820.1
gnl_Sorbi_v3.1_Sobic.004G296900.1
gnl_Sorbi_v3.1_Sobic.010G133566.1
gnl_Sorbi_v3.1_Sobic.002G069400.1
gnl_Sorbi_v3.1_Sobic.002G069100.1
gnl_Sorbi_v3.1_Sobic.005G158500.1
gnl_Sorbi_v3.1_Sobic.002G069166.1
gnl_Orysa_v7.0_LOC_Os09g39850.1
gnl_Sorbi_v3.1_Sobic.002G139400.1
gnl_Orysa_v7.0_LOC_Os07g22770.1
gnl_Sorbi_v3.1_Sobic.002G115400.1
gnl_Orysa_v7.0_LOC_Os07g22730.1
gnl_Sorbi_v3.1_Sobic.005G217400.1
gnl_Orysa_v7.0_LOC_Os05g37640.1
gnl_Orysa_v7.0_LOC_Os03g64260.1
gnl_Sorbi_v3.1_Sobic.001G002500.1
gnl_Orysa_v7.0_LOC_Os05g49010.1
gnl_Sorbi_v3.1_Sobic.009G233100.1
gnl_Orysa_v7.0_LOC_Os03g05590.1
gnl_Orysa_v7.0_LOC_Os04g18650.1
gnl_Sorbi_v3.1_Sobic.001G502300.1
gnl_Orysa_v7.0_LOC_Os10g30840.1
gnl_Sorbi_v3.1_Sobic.001G234500.1
gnl_Orysa_v7.0_LOC_Os08g44960.1
gnl_Sorbi_v3.1_Sobic.007G164900.1
gnl_Sorbi_v3.1_Sobic.002G139300.1
gnl_Orysa_v7.0_LOC_Os09g39810.1
gnl_Elagu_v2.0_p5_sc00046.V1.gene421
gnl_Elagu_v2.0_p5_sc00057.V1.gene488
gnl_Musac_v1_GSMUA_Achr6T08140_001
gnl_Musac_v1_GSMUA_Achr2T21430_001
gnl_Musac_v1_GSMUA_Achr11T09660_001
gnl_Spipo_v2_Spipo0G0175000
gnl_Musac_v1_GSMUA_Achr10T12150_001
gnl_Spipo_v2_Spipo9G0056000
gnl_Orysa_v7.0_LOC_Os04g32620.1
gnl_Sorbi_v3.1_Sobic.006G058732.1
gnl_Orysa_v7.0_LOC_Os02g32140.1
gnl_Sorbi_v3.1_Sobic.004G158901.1
gnl_Sorbi_v3.1_Sobic.007G188701.1
gnl_Sorbi_v3.1_Sobic.002G260800.1
gnl_Musac_v1_GSMUA_Achr7T18930_001
gnl_Musac_v1_GSMUA_Achr6T02310_001
gnl_Elagu_v2.0_p5_sc00011.V1.gene867
gnl_Elagu_v2.0_p5_sc00101.V1.gene157
gnl_Musac_v1_GSMUA_Achr3T06960_001
gnl_Orysa_v7.0_LOC_Os09g28440.1
gnl_Sorbi_v3.1_Sobic.002G225700.1
gnl_Orysa_v7.0_LOC_Os08g36920.1
gnl_Sorbi_v3.1_Sobic.007G156700.1
gnl_Orysa_v7.0_LOC_Os02g52670.1
gnl_Sorbi_v3.1_Sobic.004G308900.1
gnl_Spipo_v2_Spipo15G0019800
gnl_Sorbi_v3.1_Sobic.008G050600.2
gnl_Orysa_v7.0_LOC_Os02g34270.1
gnl_Sorbi_v3.1_Sobic.004G171300.1
gnl_Orysa_v7.0_LOC_Os04g34970.1
gnl_Sorbi_v3.1_Sobic.006G080200.2
gnl_Orysa_v7.0_LOC_Os05g29810.1
gnl_Sorbi_v3.1_Sobic.009G111200.1
gnl_Orysa_v7.0_LOC_Os03g08490.1
gnl_Sorbi_v3.1_Sobic.001G481300.1
gnl_Orysa_v7.0_LOC_Os09g11460.1
gnl_Orysa_v7.0_LOC_Os09g11480.2
gnl_Musac_v1_GSMUA_Achr3T29800_001
gnl_Musac_v1_GSMUA_Achr7T27150_001
gnl_Pinta_v2.0_PITA_000040911-RA
Pyrbe_038421
Pyrpy_N_03g1820.1

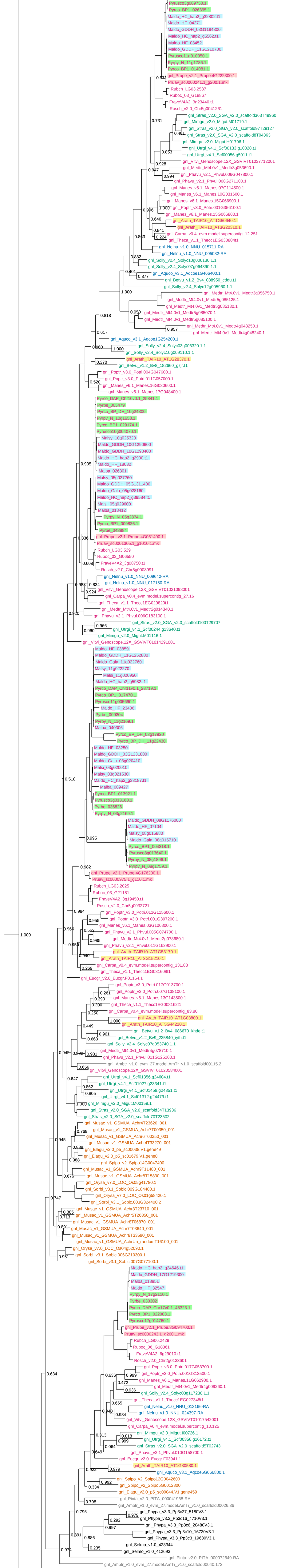

Pyrusco3g009750.1
Pyrco_BP1_026396.1
Maldo_HC_hap2_g32802.t1
Maldo_HF_04271
Maldo_GDDH_03G1194300
Maldo_HC_hap2_g5562.t1
Maldo_HF_03452
Maldo_GDDH_11G1210700
Pyrusco11g010050.1
Pyrpy_N_11g1780.1
Pyrco_BP1_014081.1
gnl_Prupe_v2.1_Prupe.4G222300.1
Pruav_sc0000241.1_g200.1.mk
Rubch_LG03.2587
Ruboc_03_G18867
FraveV4A2_3g23440.t1
Rosch_v2.0_Chr5g0041261
gnl_Stras_v2.0_SGA_v2.0_scaffold363T49960
gnl_Mimgu_v2.0_Migut.M01719.1
gnl_Stras_v2.0_SGA_v2.0_scaffold97T29127
gnl_Stras_v2.0_SGA_v2.0_scaffold8T04363
gnl_Mimgu_v2.0_Migut.H01796.1
gnl_Utrgi_v4.1_Scf00133.g10028.t1
gnl_Utrgi_v4.1_Scf00056.g5911.t1
gnl_Vitvi_Genoscope.12X_GSVIVT01037712001
gnl_Medtr_Mt4.0v1_Medtr3g053690.1
gnl_Phavu_v2.1_Phvul.006G047800.1
gnl_Phavu_v2.1_Phvul.008G271100.1
gnl_Manes_v6.1_Manes.07G114500.1
gnl_Manes_v6.1_Manes.10G031600.1
gnl_Manes_v6.1_Manes.15G066900.1
gnl_Poptr_v3.0_Potri.001G356100.1
gnl_Manes_v6.1_Manes.15G066800.1
gnl_Arath_TAIR10_AT1G50640.1
gnl_Arath_TAIR10_AT3G20310.1
gnl_Carpa_v0.4_evm.model.supercontig_12.251
gnl_Theca_v1.1_Thecc1EG030804t1
gnl_Nelnu_v1.0_NNU_015711-RA
gnl_Nelnu_v1.0_NNU_005082-RA
gnl_Solly_v2.4_Solyc10g006130.1.1
gnl_Solly_v2.4_Solyc07g064890.1.1
gnl_Aquco_v3.1_Aqcoe1G466400.1
gnl_Betvu_v1.2_Bv4_088950_cddu.t1
gnl_Solly_v2.4_Solyc12g005960.1.1
gnl_Medtr_Mt4.0v1_Medtr3g056750.1
gnl_Medtr_Mt4.0v1_Medtr5g085125.1
gnl_Medtr_Mt4.0v1_Medtr5g085130.1
gnl_Medtr_Mt4.0v1_Medtr5g085070.1
gnl_Medtr_Mt4.0v1_Medtr5g085100.1
gnl_Medtr_Mt4.0v1_Medtr4g048250.1
gnl_Medtr_Mt4.0v1_Medtr4g048240.1
gnl_Aquco_v3.1_Aqcoe1G254200.1
gnl_Solly_v2.4_Solyc03g006320.1.1
gnl_Solly_v2.4_Solyc10g009110.1.1
gnl_Arath_TAIR10_AT1G28370.1
gnl_Betvu_v1.2_Bv8_182660_gzjr.t1
gnl_Poptr_v3.0_Potri.004G047600.1
gnl_Poptr_v3.0_Potri.011G057000.1
gnl_Manes_v6.1_Manes.16G030600.1
gnl_Manes_v6.1_Manes.17G048400.1
Pyrco_DAP_Chr10v0.1_25841.1
Pyrbe_005479
Pyrco_BP_DH_10g24300
Pyrpy_N_10g1653.1
Pyrco_BP1_029174.1
Pyrusco10g004070.1
Malsy_10g025320
Maldo_GDDH_10G1290600
Maldo_GDDH_10G1290400
Maldo_HC_hap2_g2900.t1
Maldo_HF_18032
Malba_026301
Malsy_05g027260
Maldo_GDDH_05G1311400
Maldo_Gala_05g028160
Maldo_HC_hap2_g39584.t1
Malsi_05g029600
Malba_013412
Pyrpy_N_05g2874.1
Pyrco_BP1_009836.1
Pyrbe_043884
gnl_Prupe_v2.1_Prupe.4G051400.1
Pruav_sc0001305.1_g1010.1.mk
Rubch_LG03.529
Ruboc_03_G06550
FraveV4A2_3g08750.t1
Rosch_v2.0_Chr5g0008991
gnl_Nelnu_v1.0_NNU_009642-RA
gnl_Nelnu_v1.0_NNU_017150-RA
gnl_Vitvi_Genoscope.12X_GSVIVT01021098001
gnl_Carpa_v0.4_evm.model.supercontig_27.16
gnl_Theca_v1.1_Thecc1EG029820t1
gnl_Medtr_Mt4.0v1_Medtr2g014340.1
gnl_Phavu_v2.1_Phvul.006G183100.1
gnl_Stras_v2.0_SGA_v2.0_scaffold100T29707
gnl_Utrgi_v4.1_Scf00244.g13640.t1
gnl_Mimgu_v2.0_Migut.M01116.1
gnl_Vitvi_Genoscope.12X_GSVIVT01014291001
Maldo_HF_03859
Maldo_GDDH_11G1252900
Maldo_Gala_11g022760
Malsy_11g022270
Malsi_11g020860
Maldo_HC_hap2_g5882.t1
Pyrco_DAP_Chr11v0.1_28719.1
Pyrco_BP1_017470.1
Pyrusco11g005690.1
Maldo_HF_23406
Pyrbe_009204
Pyrpy_N_11g2169.1
Malba_040306
Pyrco_BP_DH_03g17920
Pyrco_BP_DH_11g22430
Maldo_HF_03250
Maldo_GDDH_03G1231800
Maldo_Gala_03g020410
Malsi_03g020010
Malsy_03g021530
Maldo_HC_hap2_g33187.t1
Malba_009427
Pyrco_BP1_013921.1
Pyrusco3g013160.1
Pyrbe_035826
Pyrpy_N_03g2169.1
Maldo_GDDH_08G1176000
Maldo_HF_07104
Malsy_08g015880
Maldo_Gala_08g015710
Pyrco_BP1_004318.1
Pyrusco8g013640.1
Pyrpy_N_08g1856.1
Pyrpy_N_08g1759.1
gnl_Prupe_v2.1_Prupe.4G176200.1
Pruav_sc0000975.1_g110.1.mk
Rubch_LG03.2025
Ruboc_03_G21181
FraveV4A2_3g19450.t1
Rosch_v2.0_Chr5g0032721
gnl_Poptr_v3.0_Potri.011G115600.1
gnl_Poptr_v3.0_Potri.001G397200.1
gnl_Manes_v6.1_Manes.03G106300.1
gnl_Phavu_v2.1_Phvul.005G074700.1
gnl_Medtr_Mt4.0v1_Medtr2g078680.1
gnl_Phavu_v2.1_Phvul.011G182900.1
gnl_Arath_TAIR10_AT1G53170.1
gnl_Arath_TAIR10_AT3G15210.1
gnl_Carpa_v0.4_evm.model.supercontig_131.83
gnl_Theca_v1.1_Thecc1EG031608t1
gnl_Eucgr_v2.0_Eucgr.F01164.1
gnl_Poptr_v3.0_Potri.017G013700.1
gnl_Poptr_v3.0_Potri.007G138100.1
gnl_Manes_v6.1_Manes.13G143500.1
gnl_Theca_v1.1_Thecc1EG008162t1
gnl_Carpa_v0.4_evm.model.supercontig_83.80
gnl_Arath_TAIR10_AT1G03800.1
gnl_Arath_TAIR10_AT5G44210.1
gnl_Betvu_v1.2_Bv4_086670_khde.t1
gnl_Betvu_v1.2_Bv9_225840_tyth.t1
gnl_Solly_v2.4_Solyc07g053740.1.1
gnl_Medtr_Mt4.0v1_Medtr4g078710.1
gnl_Phavu_v2.1_Phvul.011G125200.1
gnl_Ambtr_v1.0_evm_27.model.AmTr_v1.0_scaffold00115.2
gnl_Vitvi_Genoscope.12X_GSVIVT01020584001
gnl_Utrgi_v4.1_Scf01356.g24604.t1
gnl_Utrgi_v4.1_Scf01027.g23341.t1
gnl_Utrgi_v4.1_Scf01458.g24851.t1
gnl_Utrgi_v4.1_Scf01312.g24479.t1
gnl_Mimgu_v2.0_Migut.M00159.1
gnl_Stras_v2.0_SGA_v2.0_scaffold34T13936
gnl_Stras_v2.0_SGA_v2.0_scaffold70T23502
gnl_Musac_v1_GSMUA_Achr4T23620_001
gnl_Musac_v1_GSMUA_Achr7T00350_001
gnl_Musac_v1_GSMUA_Achr6T00250_001
gnl_Musac_v1_GSMUA_Achr4T33270_001
gnl_Elagu_v2.0_p5_sc00038.V1.gene49
gnl_Elagu_v2.0_p5_sc01679.V1.gene8
gnl_Spipo_v2_Spipo14G0047400
gnl_Musac_v1_GSMUA_Achr9T11480_001
gnl_Musac_v1_GSMUA_Achr9T15830_001
gnl_Orysa_v7.0_LOC_Os05g41780.1
gnl_Sorbi_v3.1_Sobic.009G184400.1
gnl_Orysa_v7.0_LOC_Os01g58420.1
gnl_Sorbi_v3.1_Sobic.003G324400.2
gnl_Musac_v1_GSMUA_Achr3T23710_001
gnl_Musac_v1_GSMUA_Achr5T26850_001
gnl_Musac_v1_GSMUA_Achr8T06870_001
gnl_Musac_v1_GSMUA_Achr7T03640_001
gnl_Musac_v1_GSMUA_Achr8T33590_001
gnl_Musac_v1_GSMUA_AchrUn_randomT16100_001
gnl_Orysa_v7.0_LOC_Os04g52090.1
gnl_Sorbi_v3.1_Sobic.006G210300.1
gnl_Sorbi_v3.1_Sobic.007G077100.1
Maldo_HC_hap2_g24646.t1
Maldo_GDDH_17G1219300
Malba_018851
Maldo_HF_32547
Pyrpy_N_17g2110.1
Pyrbe_030302
Pyrco_DAP_Chr17v0.1_45323.1
Pyrco_BP1_022003.1
Pyrusco17g014760.1
gnl_Prupe_v2.1_Prupe.3G094700.1
Pruav_sc0000243.1_g260.1.mk
Rubch_LG06.2429
Ruboc_06_G16361
FraveV4A2_6g29010.t1
Rosch_v2.0_Chr2g0133601
gnl_Poptr_v3.0_Potri.017G053700.1
gnl_Poptr_v3.0_Potri.001G313500.1
gnl_Manes_v6.1_Manes.11G062900.1
gnl_Medtr_Mt4.0v1_Medtr4g009260.1
gnl_Solly_v2.4_Solyc03g117230.1.1
gnl_Theca_v1.1_Thecc1EG027348t1
gnl_Nelnu_v1.0_NNU_013166-RA
gnl_Nelnu_v1.0_NNU_024397-RA
gnl_Vitvi_Genoscope.12X_GSVIVT01017542001
gnl_Carpa_v0.4_evm.model.supercontig_10.125
gnl_Mimgu_v2.0_Migut.I00726.1
gnl_Utrgi_v4.1_Scf00356.g16172.t1
gnl_Stras_v2.0_SGA_v2.0_scaffold5T02743
gnl_Phavu_v2.1_Phvul.010G158700.1
gnl_Eucgr_v2.0_Eucgr.F03941.1
gnl_Arath_TAIR10_AT1G80580.1
gnl_Aquco_v3.1_Aqcoe5G066800.1
gnl_Spipo_v2_Spipo12G0042600
gnl_Spipo_v2_Spipo5G0012800
gnl_Elagu_v2.0_p5_sc00044.V1.gene459
gnl_Pinta_v2.0_PITA_000041968-RA
gnl_Ambtr_v1.0_evm_27.model.AmTr_v1.0_scaffold00026.86
gnl_Phypa_v3.3_Pp3c27_5180V3.1
gnl_Phypa_v3.3_Pp3c16_4710V3.1
gnl_Phypa_v3.3_Pp3c6_20480V3.1
gnl_Phypa_v3.3_Pp3c10_18720V3.1
gnl_Phypa_v3.3_Pp3c3_19630V3.1
gnl_Selmo_v1.0_428344
gnl_Selmo_v1.0_412693
gnl_Pinta_v2.0_PITA_000072649-RA
gnl_Ambtr_v1.0_evm_27.model.AmTr_v1.0_scaffold00040.172

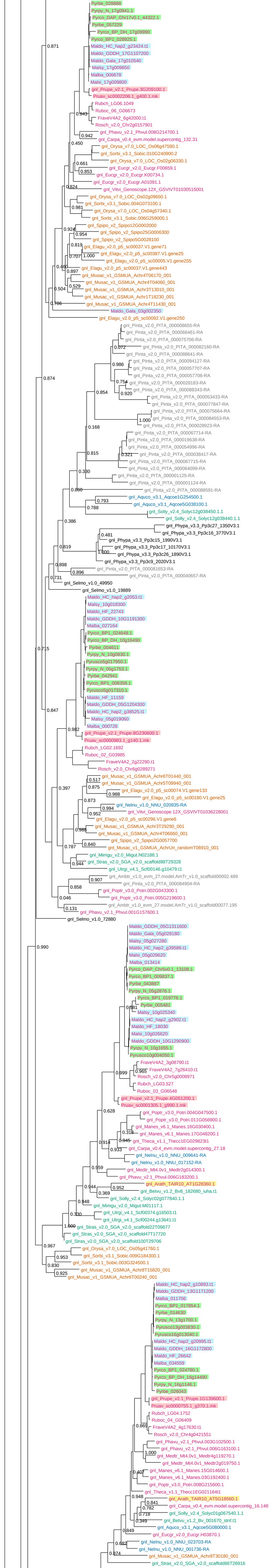
Pyrbe_028888
Pyrpy_N_17g0041.1
Pyrco_DAP_Chr17v0.1_44322.1
Pyrbe_057229
Pyrco_BP_DH_17g09990
Pyrco_BP1_028925.1
0.871
Maldo_HC_hap2_g23424.t1
Maldo_GDDH_17G1107200
Maldo_Gala_17g010540
Malsy_17g009650
Malba_006676
Malsi_17g009800
gnl_Prupe_v2.1_Prupe.3G209100.1
Pruav_sc0002206.1_g400.1.mk
Rubch_LG06.1049
0.943
Ruboc_06_G06673
FraveV4A2_6g42000.t1
Rosch_v2.0_Chr2g0157901
gnl_Phavu_v2.1_Phvul.008G214700.1
0.942
gnl_Carpa_v0.4_evm.model.supercontig_132.31
0.450
gnl_Orysa_v7.0_LOC_Os06g47590.1
gnl_Sorbi_v3.1_Sobic.010G240900.2
0.661
gnl_Orysa_v7.0_LOC_Os02g06330.1
gnl_Eucgr_v2.0_Eucgr.F00659.1
0.853
gnl_Eucgr_v2.0_Eucgr.K00734.1
gnl_Eucgr_v2.0_Eucgr.A01091.1
0.824
gnl_Vitvi_Genoscope.12X_GSVIVT01030515001
gnl_Orysa_v7.0_LOC_Os02g09650.1
gnl_Sorbi_v3.1_Sobic.004G073100.1
0.981
gnl_Orysa_v7.0_LOC_Os04g57340.1
gnl_Sorbi_v3.1_Sobic.006G259000.1
gnl_Spipo_v2_Spipo12G0002000
0.924
gnl_Spipo_v2_Spipo25G0006300
0.954
gnl_Spipo_v2_Spipo5G0028100
0.818
gnl_Elagu_v2.0_p5_sc00037.V1.gene71
gnl_Elagu_v2.0_p5_sc00387.V1.gene25
0.707
1.000
gnl_Elagu_v2.0_p5_sc00005.V1.gene255
0.490
gnl_Elagu_v2.0_p5_sc00037.V1.gene443
0.897
gnl_Musac_v1_GSMUA_Achr4T06170_001
gnl_Musac_v1_GSMUA_Achr4T04060_001
0.529
0.504
gnl_Musac_v1_GSMUA_Achr3T13010_001
gnl_Musac_v1_GSMUA_Achr1T18230_001
0.786
gnl_Musac_v1_GSMUA_Achr4T11430_001
Maldo_Gala_03g002350
gnl_Elagu_v2.0_p5_sc00092.V1.gene250
gnl_Pinta_v2.0_PITA_000008655-RA
gnl_Pinta_v2.0_PITA_000066461-RA
gnl_Pinta_v2.0_PITA_000075706-RA
0.072
gnl_Pinta_v2.0_PITA_000082160-RA
gnl_Pinta_v2.0_PITA_000088641-RA
gnl_Pinta_v2.0_PITA_000094127-RA
0.986
gnl_Pinta_v2.0_PITA_000057707-RA
0.874
gnl_Pinta_v2.0_PITA_000057708-RA
0.754
gnl_Pinta_v2.0_PITA_000028183-RA
gnl_Pinta_v2.0_PITA_000088343-RA
0.854
0.920
gnl_Pinta_v2.0_PITA_000053433-RA
gnl_Pinta_v2.0_PITA_000077847-RA
gnl_Pinta_v2.0_PITA_000075664-RA
1.000
gnl_Pinta_v2.0_PITA_000084553-RA
gnl_Pinta_v2.0_PITA_000028923-RA
0.168
gnl_Pinta_v2.0_PITA_000067714-RA
gnl_Pinta_v2.0_PITA_000019638-RA
gnl_Pinta_v2.0_PITA_000054996-RA
0.815
0.321
gnl_Pinta_v2.0_PITA_000038417-RA
gnl_Pinta_v2.0_PITA_000067715-RA
gnl_Pinta_v2.0_PITA_000064099-RA
0.330
gnl_Pinta_v2.0_PITA_000001125-RA
gnl_Pinta_v2.0_PITA_000001124-RA
0.860
gnl_Pinta_v2.0_PITA_000088591-RA
gnl_Aquco_v3.1_Aqcoe1G254500.1
0.793
gnl_Aquco_v3.1_Aqcoe5G038100.1
0.788
gnl_Solly_v2.4_Solyc12g038450.1.1
gnl_Solly_v2.4_Solyc12g038440.1.1
0.386
gnl_Phypa_v3.3_Pp3c27_1350V3.1
gnl_Phypa_v3.3_Pp3c16_3770V3.1
0.481
gnl_Phypa_v3.3_Pp3c15_1990V3.1
0.819
gnl_Phypa_v3.3_Pp3c17_10170V3.1
1.000
gnl_Phypa_v3.3_Pp3c26_1890V3.1
gnl_Phypa_v3.3_Pp3c9_2020V3.1
0.898
gnl_Pinta_v2.0_PITA_000081653-RA
0.896
0.731
gnl_Pinta_v2.0_PITA_000040657-RA
gnl_Selmo_v1.0_49950
gnl_Selmo_v1.0_19889
Maldo_HC_hap2_g2053.t1
Malsy_10g018300
Maldo_HF_22743
Maldo_GDDH_10G1191300
Malba_027164
Pyrco_BP1_024648.1
Pyrco_BP_DH_10g16450
Pyrbe_004611
0.715
Pyrpy_N_10g0830.1
Pyrusco5g017950.1
Pyrpy_N_05g1753.1
Pyrbe_042941
Pyrco_BP1_008358.1
Pyrusco5g017310.1
Maldo_HF_11159
Maldo_GDDH_05G1204300
0.847
Maldo_HC_hap2_g38525.t1
Malsy_05g019060
Malba_000728
0.982
gnl_Prupe_v2.1_Prupe.8G230600.1
Pruav_sc0000983.1_g140.1.mk
Rubch_LG02.1692
Ruboc_02_G03985
FraveV4A2_2g22290.t1
Rosch_v2.0_Chr6g0289271
0.517
gnl_Musac_v1_GSMUA_Achr6T01440_001
gnl_Musac_v1_GSMUA_Achr5T09940_001
0.397
0.875
0.988
gnl_Elagu_v2.0_p5_sc00074.V1.gene133
gnl_Elagu_v2.0_p5_sc00180.V1.gene25
0.873
gnl_Nelnu_v1.0_NNU_020935-RA
0.994
0.952
gnl_Vitvi_Genoscope.12X_GSVIVT01036228001
gnl_Elagu_v2.0_p5_sc00290.V1.gene6
gnl_Musac_v1_GSMUA_Achr3T29290_001
0.988
gnl_Musac_v1_GSMUA_Achr4T06660_001
gnl_Spipo_v2_Spipo2G0057700
0.840
0.787
gnl_Musac_v1_GSMUA_AchrUn_randomT06910_001
gnl_Mimgu_v2.0_Migut.N02188.1
gnl_Stras_v2.0_SGA_v2.0_scaffold98T29328
0.944
gnl_Utrgi_v4.1_Scf00146.g10479.t1
gnl_Ambtr_v1.0_evm_27.model.AmTr_v1.0_scaffold00002.489
0.907
gnl_Pinta_v2.0_PITA_000084904-RA
0.858
gnl_Poptr_v3.0_Potri.002G043300.1
0.046
gnl_Poptr_v3.0_Potri.005G219600.1
gnl_Ambtr_v1.0_evm_27.model.AmTr_v1.0_scaffold00077.195
0.131
gnl_Phavu_v2.1_Phvul.001G157600.1
gnl_Selmo_v1.0_72880
Maldo_GDDH_05G1311600
Maldo_Gala_05g028180
Malsy_05g027280
0.990
Maldo_HC_hap2_g39586.t1
Malsi_05g029620
Malba_013414
Pyrco_DAP_Chr5v0.1_13108.1
Pyrco_BP1_009837.1
Pyrbe_043887
Pyrpy_N_05g2876.1
Pyrco_BP1_019776.1
Pyrbe_005481
0.981
Malsy_10g025340
Maldo_HC_hap2_g2902.t1
Maldo_HF_18030
Malsi_10g026620
Maldo_GDDH_10G1290900
Pyrpy_N_10g1655.1
Pyrusco10g004050.1
FraveV4A2_3g08790.t1
0.999
0.965
FraveV4A2_7g26410.t1
Rosch_v2.0_Chr5g0008971
Rubch_LG03.527
Ruboc_03_G06548
gnl_Prupe_v2.1_Prupe.4G051200.1
Pruav_sc0001305.1_g990.1.mk
0.628
gnl_Poptr_v3.0_Potri.004G047500.1
gnl_Poptr_v3.0_Potri.011G056900.1
gnl_Manes_v6.1_Manes.16G030400.1
0.318
gnl_Manes_v6.1_Manes.17G048200.1
0.914
0.945
gnl_Theca_v1.1_Thecc1EG029823t1
0.933
gnl_Carpa_v0.4_evm.model.supercontig_27.18
gnl_Nelnu_v1.0_NNU_009641-RA
gnl_Nelnu_v1.0_NNU_017152-RA
0.959
gnl_Medtr_Mt4.0v1_Medtr2g014300.1
gnl_Phavu_v2.1_Phvul.006G183200.1
gnl_Arath_TAIR10_AT1G28360.1
0.944
0.952
0.369
gnl_Betvu_v1.2_Bv8_182680_iuha.t1
0.948
gnl_Solly_v2.4_Solyc02g077840.1.1
gnl_Mimgu_v2.0_Migut.M01117.1
0.330
gnl_Utrgi_v4.1_Scf00374.g16503.t1
gnl_Utrgi_v4.1_Scf00244.g13641.t1
1.000
gnl_Stras_v2.0_SGA_v2.0_scaffold22T09677
gnl_Stras_v2.0_SGA_v2.0_scaffold47T17720
gnl_Stras_v2.0_SGA_v2.0_scaffold100T29706
0.967
gnl_Orysa_v7.0_LOC_Os05g41760.1
0.953
gnl_Sorbi_v3.1_Sobic.009G184300.1
0.830
gnl_Sorbi_v3.1_Sobic.003G324500.1
gnl_Musac_v1_GSMUA_Achr8T15820_001
0.925
gnl_Musac_v1_GSMUA_Achr6T00240_001
Maldo_HC_hap2_g10893.t1
Maldo_GDDH_13G1171200
Malba_011706
Pyrco_BP1_017854.1
Pyrbe_014630
Pyrpy_N_13g1700.1
Pyrusco13g003830.1
Pyrusco16g013040.1
Maldo_HC_hap2_g20995.t1
Maldo_GDDH_16G1172800
Maldo_HF_26642
Malba_034559
Pyrco_BP1_024780.1
Pyrco_BP_DH_16g14490
Pyrpy_N_16g1146.1
Pyrbe_026543
gnl_Prupe_v2.1_Prupe.1G139600.1
Pruav_sc0000755.1_g370.1.mk
Rubch_LG04.1752
Ruboc_04_G06409
0.869
FraveV4A2_4g17630.t1
Rosch_v2.0_Chr4g0421551
gnl_Phavu_v2.1_Phvul.003G102500.1
gnl_Phavu_v2.1_Phvul.006G163100.1
1.000
gnl_Medtr_Mt4.0v1_Medtr4g119270.1
gnl_Medtr_Mt4.0v1_Medtr2g019750.1
0.407
gnl_Manes_v6.1_Manes.15G014600.1
gnl_Manes_v6.1_Manes.03G192400.1
gnl_Poptr_v3.0_Potri.008G215600.1
gnl_Theca_v1.1_Thecc1EG021164t1
0.948
gnl_Arath_TAIR10_AT5G18560.1
0.841
0.782
gnl_Carpa_v0.4_evm.model.supercontig_16.148
0.718
gnl_Solly_v2.4_Solyc01g067540.1.1
0.349
gnl_Betvu_v1.2_Bv_001670_stnf.t1
0.849
gnl_Aquco_v3.1_Aqcoe5G080000.1
gnl_Eucgr_v2.0_Eucgr.H03870.1
0.682
gnl_Nelnu_v1.0_NNU_023703-RA
gnl_Nelnu_v1.0_NNU_001736-RA
0.874
gnl_Musac_v1_GSMUA_Achr8T30180_001
gnl_Stras_v2.0_SGA_v2.0_scaffold86T26916

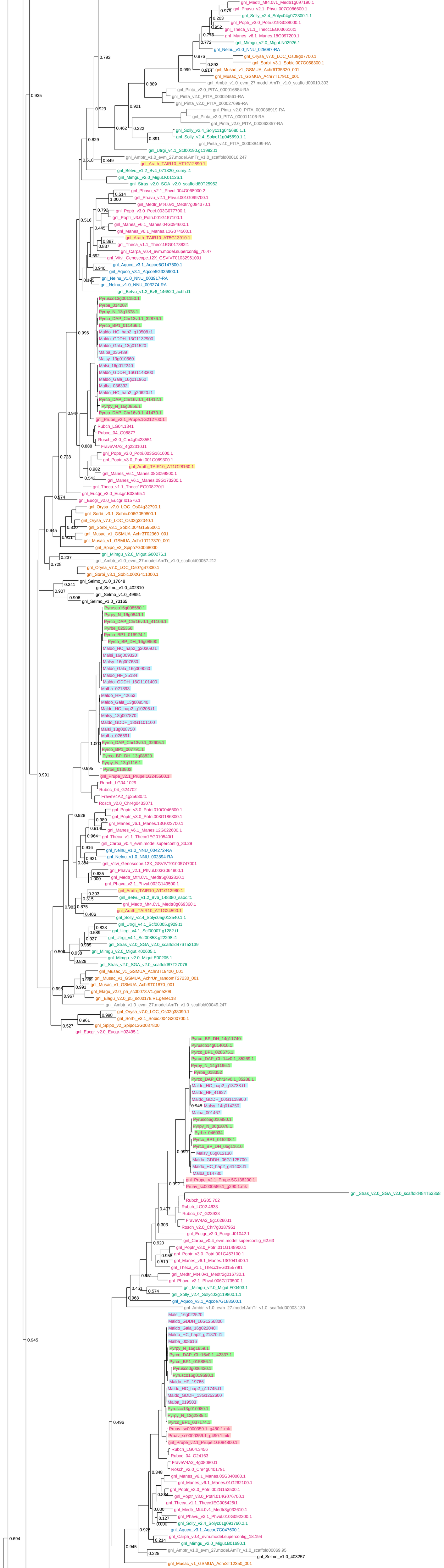

gnl_Medtr_Mt4.0v1_Medtr1g097190.1
gnl_Phavu_v2.1_Phvul.007G086600.1
gnl_Solly_v2.4_Solyc04g072300.1.1
gnl_Poptr_v3.0_Potri.019G088000.1
gnl_Theca_v1.1_Thecc1EG036618t1
gnl_Manes_v6.1_Manes.18G097200.1
gnl_Mimgu_v2.0_Migut.N02926.1
gnl_Nelnu_v1.0_NNU_025087-RA
gnl_Orysa_v7.0_LOC_Os08g07700.1
gnl_Sorbi_v3.1_Sobic.007G058300.1
gnl_Musac_v1_GSMUA_Achr6T35320_001
gnl_Musac_v1_GSMUA_Achr7T17910_001
gnl_Ambtr_v1.0_evm_27.model.AmTr_v1.0_scaffold00010.303
gnl_Pinta_v2.0_PITA_000016884-RA
gnl_Pinta_v2.0_PITA_000024561-RA
gnl_Pinta_v2.0_PITA_000027699-RA
gnl_Pinta_v2.0_PITA_000038919-RA
gnl_Pinta_v2.0_PITA_000011106-RA
gnl_Pinta_v2.0_PITA_000063857-RA
gnl_Solly_v2.4_Solyc11g045680.1.1
gnl_Solly_v2.4_Solyc11g045690.1.1
gnl_Pinta_v2.0_PITA_000038499-RA
gnl_Utrgi_v4.1_Scf00190.g11982.t1
gnl_Ambtr_v1.0_evm_27.model.AmTr_v1.0_scaffold00016.247
gnl_Arath_TAIR10_AT1G12890.1
gnl_Betvu_v1.2_Bv4_071820_sumy.t1
gnl_Mimgu_v2.0_Migut.K01126.1
gnl_Stras_v2.0_SGA_v2.0_scaffold80T25952
gnl_Phavu_v2.1_Phvul.004G068900.2
gnl_Phavu_v2.1_Phvul.001G099700.1
gnl_Medtr_Mt4.0v1_Medtr7g084370.1
gnl_Poptr_v3.0_Potri.003G077700.1
gnl_Poptr_v3.0_Potri.001G157100.1
gnl_Manes_v6.1_Manes.04G094600.1
gnl_Manes_v6.1_Manes.11G074500.1
gnl_Arath_TAIR10_AT5G13910.1
gnl_Theca_v1.1_Thecc1EG017382t1
gnl_Carpa_v0.4_evm.model.supercontig_70.47
gnl_Vitvi_Genoscope.12X_GSVIVT01032961001
gnl_Aquco_v3.1_Aqcoe6G147500.1
gnl_Aquco_v3.1_Aqcoe5G335900.1
gnl_Nelnu_v1.0_NNU_003917-RA
gnl_Nelnu_v1.0_NNU_003274-RA
gnl_Betvu_v1.2_Bv6_146520_achh.t1
Pyrusco13g001150.1
Pyrbe_014207
Pyrpy_N_13g1378.1
Pyrco_DAP_Chr13v0.1_32876.1
Pyrco_BP1_011466.1
Maldo_HC_hap2_g10508.t1
Maldo_GDDH_13G1132900
Maldo_Gala_13g011520
Malba_036439
Malsy_13g010560
Malsi_16g012240
Maldo_GDDH_16G1143300
Maldo_Gala_16g011960
Malba_038392
Maldo_HC_hap2_g20620.t1
Pyrco_DAP_Chr16v0.1_41412.1
Pyrpy_N_16g0856.1
Pyrco_DAP_Chr16v0.1_41470.1
gnl_Prupe_v2.1_Prupe.1G212700.1
Rubch_LG04.1341
Ruboc_04_G08877
Rosch_v2.0_Chr4g0428551
FraveV4A2_4g22310.t1
gnl_Poptr_v3.0_Potri.003G161000.1
gnl_Poptr_v3.0_Potri.001G069300.1
gnl_Arath_TAIR10_AT1G28160.1
gnl_Manes_v6.1_Manes.08G099800.1
gnl_Manes_v6.1_Manes.09G173200.1
gnl_Theca_v1.1_Thecc1EG008270t1
gnl_Eucgr_v2.0_Eucgr.B03565.1
gnl_Eucgr_v2.0_Eucgr.I01576.1
gnl_Orysa_v7.0_LOC_Os04g32790.1
gnl_Sorbi_v3.1_Sobic.006G059800.1
gnl_Orysa_v7.0_LOC_Os02g32040.1
gnl_Sorbi_v3.1_Sobic.004G159500.1
gnl_Musac_v1_GSMUA_Achr3T02360_001
gnl_Musac_v1_GSMUA_Achr10T17370_001
gnl_Spipo_v2_Spipo7G0068000
gnl_Mimgu_v2.0_Migut.G00276.1
gnl_Ambtr_v1.0_evm_27.model.AmTr_v1.0_scaffold00057.212
gnl_Orysa_v7.0_LOC_Os07g47330.1
gnl_Sorbi_v3.1_Sobic.002G411000.1
gnl_Selmo_v1.0_17648
gnl_Selmo_v1.0_402810
gnl_Selmo_v1.0_49951
gnl_Selmo_v1.0_73165
Pyrusco16g008550.1
Pyrpy_N_16g0849.1
Pyrco_DAP_Chr16v0.1_41106.1
Pyrbe_025356
Pyrco_BP1_016924.1
Pyrco_BP_DH_16g08590
Maldo_HC_hap2_g20309.t1
Malsi_16g009320
Malsy_16g007680
Maldo_Gala_16g009060
Maldo_HF_35134
Maldo_GDDH_16G1101400
Malba_021893
Maldo_HF_42652
Maldo_Gala_13g008540
Maldo_HC_hap2_g10206.t1
Malsy_13g007870
Maldo_GDDH_13G1101100
Malsi_13g008750
Malba_026591
Pyrco_DAP_Chr13v0.1_32605.1
Pyrco_BP1_007791.1
Pyrco_BP_DH_13g08820
Pyrpy_N_13g1116.1
Pyrbe_013902
gnl_Prupe_v2.1_Prupe.1G245500.1
Rubch_LG04.1029
Ruboc_04_G24702
FraveV4A2_4g25630.t1
Rosch_v2.0_Chr4g0433071
gnl_Poptr_v3.0_Potri.010G046600.1
gnl_Poptr_v3.0_Potri.008G186300.1
gnl_Manes_v6.1_Manes.13G023700.1
gnl_Manes_v6.1_Manes.12G022600.1
gnl_Theca_v1.1_Thecc1EG010540t1
gnl_Carpa_v0.4_evm.model.supercontig_33.29
gnl_Nelnu_v1.0_NNU_004272-RA
gnl_Nelnu_v1.0_NNU_002894-RA
gnl_Vitvi_Genoscope.12X_GSVIVT01005747001
gnl_Phavu_v2.1_Phvul.003G064800.1
gnl_Medtr_Mt4.0v1_Medtr5g032820.1
gnl_Phavu_v2.1_Phvul.002G149500.1
gnl_Arath_TAIR10_AT1G12980.1
gnl_Betvu_v1.2_Bv6_148360_saoc.t1
gnl_Medtr_Mt4.0v1_Medtr8g069360.1
gnl_Arath_TAIR10_AT1G24590.1
gnl_Solly_v2.4_Solyc05g013540.1.1
gnl_Utrgi_v4.1_Scf00005.g929.t1
gnl_Utrgi_v4.1_Scf00007.g1282.t1
gnl_Utrgi_v4.1_Scf00858.g22298.t1
gnl_Stras_v2.0_SGA_v2.0_scaffold476T52139
gnl_Mimgu_v2.0_Migut.K00605.1
gnl_Mimgu_v2.0_Migut.E00205.1
gnl_Stras_v2.0_SGA_v2.0_scaffold87T27076
gnl_Musac_v1_GSMUA_Achr3T19420_001
gnl_Musac_v1_GSMUA_AchrUn_randomT27230_001
gnl_Musac_v1_GSMUA_Achr9T01870_001
gnl_Elagu_v2.0_p5_sc00073.V1.gene208
gnl_Elagu_v2.0_p5_sc00178.V1.gene118
gnl_Ambtr_v1.0_evm_27.model.AmTr_v1.0_scaffold00049.247
gnl_Orysa_v7.0_LOC_Os02g38090.1
gnl_Sorbi_v3.1_Sobic.004G200700.1
gnl_Spipo_v2_Spipo13G0037800
gnl_Eucgr_v2.0_Eucgr.H02495.1
Pyrco_BP_DH_14g11740
Pyrusco14g014010.1
Pyrco_BP1_028675.1
Pyrco_DAP_Chr14v0.1_35269.1
Pyrpy_N_14g1196.1
Pyrbe_018352
Pyrco_DAP_Chr14v0.1_35288.1
Maldo_HC_hap2_g13738.t1
Maldo_HF_41627
Maldo_GDDH_00G1118900
Malsy_14g014250
Malba_001467
Pyrusco6g010880.1
Pyrpy_N_06g1078.1
Pyrbe_046034
Pyrco_BP1_015238.1
Pyrco_BP_DH_06g11610
Malsy_06g012130
Maldo_GDDH_06G1125700
Maldo_HC_hap2_g41408.t1
Malba_014730
gnl_Prupe_v2.1_Prupe.5G136200.1
Pruav_sc0000589.1_g290.1.mk
gnl_Stras_v2.0_SGA_v2.0_scaffold484T52358
Rubch_LG05.702
Rubch_LG02.4633
Ruboc_07_G23993
FraveV4A2_5g10260.t1
Rosch_v2.0_Chr7g0187951
gnl_Eucgr_v2.0_Eucgr.J01042.1
gnl_Carpa_v0.4_evm.model.supercontig_62.63
gnl_Poptr_v3.0_Potri.011G148900.1
gnl_Poptr_v3.0_Potri.001G453100.1
gnl_Manes_v6.1_Manes.13G041400.1
gnl_Theca_v1.1_Thecc1EG015579t1
gnl_Medtr_Mt4.0v1_Medtr2g016730.1
gnl_Phavu_v2.1_Phvul.006G173500.1
gnl_Mimgu_v2.0_Migut.F00403.1
gnl_Solly_v2.4_Solyc03g119800.1.1
gnl_Aquco_v3.1_Aqcoe7G188500.1
gnl_Ambtr_v1.0_evm_27.model.AmTr_v1.0_scaffold00003.139
Malsi_16g022520
Maldo_GDDH_16G1256800
Maldo_Gala_16g022040
Maldo_HC_hap2_g21870.t1
Malba_008616
Pyrpy_N_16g1859.1
Pyrco_DAP_Chr16v0.1_42337.1
Pyrco_BP1_015886.1
Pyrusco6g006430.1
Pyrusco16g019590.1
Maldo_HF_19766
Maldo_HC_hap2_g11745.t1
Maldo_GDDH_13G1252600
Malba_019503
Pyrusco13g010980.1
Pyrpy_N_13g2095.1
Pyrco_BP1_037174.1
Pruav_sc0000359.1_g480.1.mk
Pruav_sc0000359.1_g490.1.mk
gnl_Prupe_v2.1_Prupe.1G084800.1
Rubch_LG04.3456
Ruboc_04_G24163
FraveV4A2_4g08080.t1
Rosch_v2.0_Chr4g0401791
gnl_Manes_v6.1_Manes.05G040000.1
gnl_Manes_v6.1_Manes.01G262100.1
gnl_Poptr_v3.0_Potri.002G153500.1
gnl_Poptr_v3.0_Potri.014G076700.1
gnl_Theca_v1.1_Thecc1EG005425t1
gnl_Medtr_Mt4.0v1_Medtr8g032610.1
gnl_Phavu_v2.1_Phvul.010G092300.1
gnl_Solly_v2.4_Solyc01g091760.2.1
gnl_Aquco_v3.1_Aqcoe7G047600.1
gnl_Carpa_v0.4_evm.model.supercontig_18.194
gnl_Mimgu_v2.0_Migut.B01690.1
gnl_Ambtr_v1.0_evm_27.model.AmTr_v1.0_scaffold00069.95
gnl_Selmo_v1.0_403257
gnl_Musac_v1_GSMUA_Achr3T12350_001

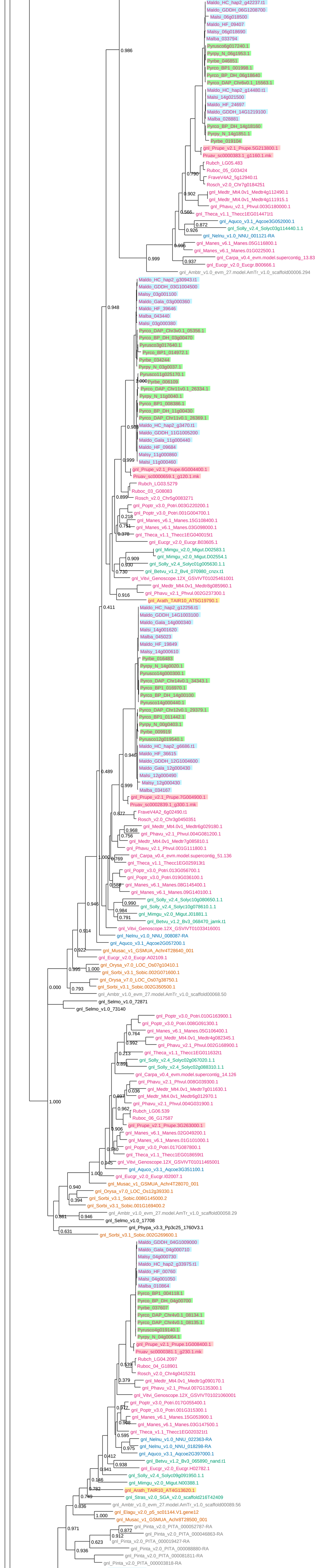
Maldo_HC_hap2_g42237.t1
Maldo_GDDH_06G1208700
Malsi_06g018500
Maldo_HF_09407
Malsy_06g018690
Malba_033794
Pyrusco6g017240.1
Pyrpy_N_06g1953.1
Pyrbe_046851
Pyrco_BP1_001998.1
Pyrco_BP_DH_06g18640
Pyrco_DAP_Chr6v0.1_15563.1
Maldo_HC_hap2_g14480.t1
Malsi_14g021500
Maldo_HF_24697
Maldo_GDDH_14G1219100
Malba_028881
Pyrco_BP_DH_14g18160
Pyrpy_N_14g1851.1
Pyrbe_019104
gnl_Prupe_v2.1_Prupe.5G213800.1
Pruav_sc0000383.1_g1160.1.mk
Rubch_LG05.483
Ruboc_05_G03424
FraveV4A2_5g12940.t1
Rosch_v2.0_Chr7g0184251
gnl_Medtr_Mt4.0v1_Medtr4g112490.1
gnl_Medtr_Mt4.0v1_Medtr4g111915.1
gnl_Phavu_v2.1_Phvul.003G180000.1
gnl_Theca_v1.1_Thecc1EG014471t1
gnl_Aquco_v3.1_Aqcoe3G052000.1
gnl_Solly_v2.4_Solyc03g114440.1.1
gnl_Nelnu_v1.0_NNU_001121-RA
gnl_Manes_v6.1_Manes.05G116800.1
gnl_Manes_v6.1_Manes.01G022500.1
gnl_Carpa_v0.4_evm.model.supercontig_13.83
gnl_Eucgr_v2.0_Eucgr.B00666.1
gnl_Ambtr_v1.0_evm_27.model.AmTr_v1.0_scaffold00006.294
Maldo_HC_hap2_g30943.t1
Maldo_GDDH_03G1004500
Malsy_03g001100
Maldo_Gala_03g000360
Maldo_HF_39646
Malba_043440
Malsi_03g000380
Pyrco_DAP_Chr3v0.1_05356.1
Pyrco_BP_DH_03g00470
Pyrusco3g017640.1
Pyrco_BP1_014972.1
Pyrbe_034244
Pyrpy_N_03g0037.1
Pyrusco11g025170.1
Pyrbe_006109
Pyrco_DAP_Chr11v0.1_26334.1
Pyrpy_N_11g0040.1
Pyrco_BP1_008386.1
Pyrco_BP_DH_11g00430
Pyrco_DAP_Chr11v0.1_26389.1
Maldo_HC_hap2_g3470.t1
Maldo_GDDH_11G1005200
Maldo_Gala_11g000440
Maldo_HF_09684
Malsy_11g000860
Malsi_11g000460
gnl_Prupe_v2.1_Prupe.6G004400.1
Pruav_sc0000659.1_g120.1.mk
Rubch_LG03.5279
Ruboc_03_G08083
Rosch_v2.0_Chr5g0083271
gnl_Poptr_v3.0_Potri.003G220200.1
gnl_Poptr_v3.0_Potri.001G004700.1
gnl_Manes_v6.1_Manes.15G108400.1
gnl_Manes_v6.1_Manes.03G098000.1
gnl_Theca_v1.1_Thecc1EG040015t1
gnl_Eucgr_v2.0_Eucgr.B03505.1
gnl_Mimgu_v2.0_Migut.D02583.1
gnl_Mimgu_v2.0_Migut.D02554.1
gnl_Solly_v2.4_Solyc01g005630.1.1
gnl_Betvu_v1.2_Bv4_070980_cnzx.t1
gnl_Vitvi_Genoscope.12X_GSVIVT01025461001
gnl_Medtr_Mt4.0v1_Medtr8g085960.1
gnl_Phavu_v2.1_Phvul.002G237300.1
gnl_Arath_TAIR10_AT5G19790.1
Maldo_HC_hap2_g12256.t1
Maldo_GDDH_14G1003100
Maldo_Gala_14g000340
Malsi_14g001620
Malba_045023
Maldo_HF_19849
Malsy_14g000610
Pyrbe_016483
Pyrpy_N_14g0020.1
Pyrusco14g000300.1
Pyrco_DAP_Chr14v0.1_34343.1
Pyrco_BP1_016970.1
Pyrco_BP_DH_14g00100
Pyrusco14g000440.1
Pyrco_DAP_Chr12v0.1_29379.1
Pyrco_BP1_011442.1
Pyrpy_N_00g0403.1
Pyrbe_009019
Pyrusco12g019540.1
Maldo_HC_hap2_g6688.t1
Maldo_HF_38615
Maldo_GDDH_12G1004600
Maldo_Gala_12g000430
Malsi_12g000490
Malsy_12g000430
Malba_034167
gnl_Prupe_v2.1_Prupe.7G004900.1
Pruav_sc0002839.1_g300.1.mk
FraveV4A2_6g02490.t1
Rosch_v2.0_Chr3g0450351
gnl_Medtr_Mt4.0v1_Medtr6g029180.1
gnl_Phavu_v2.1_Phvul.004G081200.1
gnl_Medtr_Mt4.0v1_Medtr7g085810.1
gnl_Phavu_v2.1_Phvul.001G111800.1
gnl_Carpa_v0.4_evm.model.supercontig_51.136
gnl_Theca_v1.1_Thecc1EG025913t1
gnl_Poptr_v3.0_Potri.013G056700.1
gnl_Poptr_v3.0_Potri.019G036100.1
gnl_Manes_v6.1_Manes.08G145400.1
gnl_Manes_v6.1_Manes.09G140100.1
gnl_Solly_v2.4_Solyc10g080650.1.1
gnl_Solly_v2.4_Solyc10g078610.1.1
gnl_Mimgu_v2.0_Migut.J01881.1
gnl_Betvu_v1.2_Bv3_068470_jamk.t1
gnl_Vitvi_Genoscope.12X_GSVIVT01033416001
gnl_Nelnu_v1.0_NNU_008087-RA
gnl_Aquco_v3.1_Aqcoe2G057200.1
gnl_Musac_v1_GSMUA_Achr4T28640_001
gnl_Eucgr_v2.0_Eucgr.A02109.1
gnl_Orysa_v7.0_LOC_Os07g10410.1
gnl_Sorbi_v3.1_Sobic.002G071600.1
gnl_Orysa_v7.0_LOC_Os07g38750.1
gnl_Sorbi_v3.1_Sobic.002G350500.1
gnl_Ambtr_v1.0_evm_27.model.AmTr_v1.0_scaffold00068.50
gnl_Selmo_v1.0_72871
gnl_Selmo_v1.0_73140
gnl_Poptr_v3.0_Potri.010G163900.1
gnl_Poptr_v3.0_Potri.008G091300.1
gnl_Manes_v6.1_Manes.05G106400.1
gnl_Medtr_Mt4.0v1_Medtr4g082345.1
gnl_Phavu_v2.1_Phvul.002G168900.1
gnl_Theca_v1.1_Thecc1EG011632t1
gnl_Solly_v2.4_Solyc02g067020.1.1
gnl_Solly_v2.4_Solyc02g088310.1.1
gnl_Carpa_v0.4_evm.model.supercontig_14.126
gnl_Phavu_v2.1_Phvul.008G039300.1
gnl_Medtr_Mt4.0v1_Medtr7g011630.1
gnl_Medtr_Mt4.0v1_Medtr6g012970.1
gnl_Phavu_v2.1_Phvul.004G031900.1
Rubch_LG06.539
Ruboc_06_G17587
gnl_Prupe_v2.1_Prupe.3G263000.1
gnl_Manes_v6.1_Manes.02G049200.1
gnl_Manes_v6.1_Manes.01G101000.1
gnl_Poptr_v3.0_Potri.017G087800.1
gnl_Theca_v1.1_Thecc1EG018659t1
gnl_Vitvi_Genoscope.12X_GSVIVT01011465001
gnl_Aquco_v3.1_Aqcoe3G351100.1
gnl_Eucgr_v2.0_Eucgr.I02007.1
gnl_Musac_v1_GSMUA_Achr4T28070_001
gnl_Orysa_v7.0_LOC_Os12g39330.1
gnl_Sorbi_v3.1_Sobic.008G145000.2
gnl_Sorbi_v3.1_Sobic.001G169400.2
gnl_Ambtr_v1.0_evm_27.model.AmTr_v1.0_scaffold00058.29
gnl_Selmo_v1.0_17708
gnl_Phypa_v3.3_Pp3c25_1760V3.1
gnl_Sorbi_v3.1_Sobic.002G269600.1
Maldo_GDDH_04G1009000
Maldo_Gala_04g000710
Malsy_04g000730
Maldo_HC_hap2_g33975.t1
Maldo_HF_00760
Malsi_04g001050
Malba_010864
Pyrco_BP1_004118.1
Pyrco_BP_DH_04g00700
Pyrbe_037607
Pyrco_DAP_Chr4v0.1_08134.1
Pyrco_DAP_Chr4v0.1_08135.1
Pyrusco4g019140.1
Pyrpy_N_04g0064.1
gnl_Prupe_v2.1_Prupe.1G009400.1
Pruav_sc0000381.1_g230.1.mk
Rubch_LG04.2097
Ruboc_04_G18901
Rosch_v2.0_Chr4g0415231
gnl_Medtr_Mt4.0v1_Medtr1g090170.1
gnl_Phavu_v2.1_Phvul.007G135300.1
gnl_Vitvi_Genoscope.12X_GSVIVT01021060001
gnl_Poptr_v3.0_Potri.017G055400.1
gnl_Poptr_v3.0_Potri.001G315300.1
gnl_Manes_v6.1_Manes.15G053900.1
gnl_Manes_v6.1_Manes.03G147500.1
gnl_Theca_v1.1_Thecc1EG020321t1
gnl_Nelnu_v1.0_NNU_022363-RA
gnl_Nelnu_v1.0_NNU_018298-RA
gnl_Aquco_v3.1_Aqcoe2G397000.1
gnl_Betvu_v1.2_Bv3_065890_nand.t1
gnl_Eucgr_v2.0_Eucgr.H02782.1
gnl_Solly_v2.4_Solyc09g091950.1.1
gnl_Mimgu_v2.0_Migut.N00388.1
gnl_Arath_TAIR10_AT4G13620.1
gnl_Stras_v2.0_SGA_v2.0_scaffold216T42409
gnl_Ambtr_v1.0_evm_27.model.AmTr_v1.0_scaffold00089.56
gnl_Elagu_v2.0_p5_sc01144.V1.gene12
gnl_Musac_v1_GSMUA_Achr8T28500_001
gnl_Pinta_v2.0_PITA_000052787-RA
gnl_Pinta_v2.0_PITA_000046863-RA
gnl_Pinta_v2.0_PITA_000019427-RA
gnl_Pinta_v2.0_PITA_000088880-RA
gnl_Pinta_v2.0_PITA_000081811-RA
gnl_Pinta_v2.0_PITA_000003818-RA

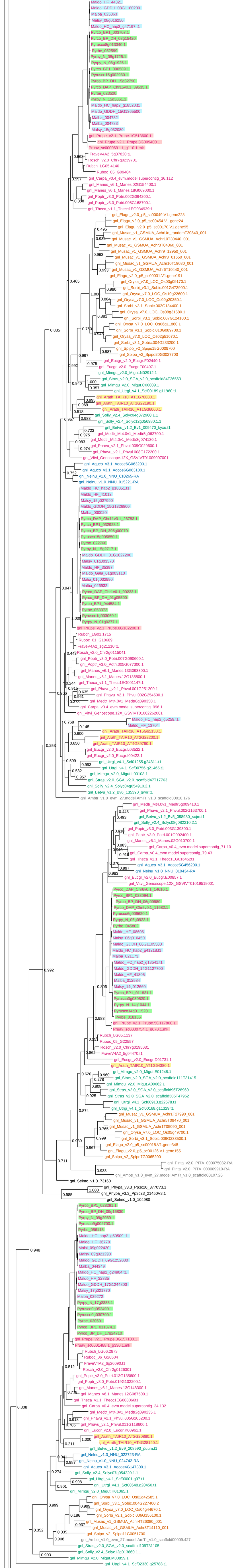

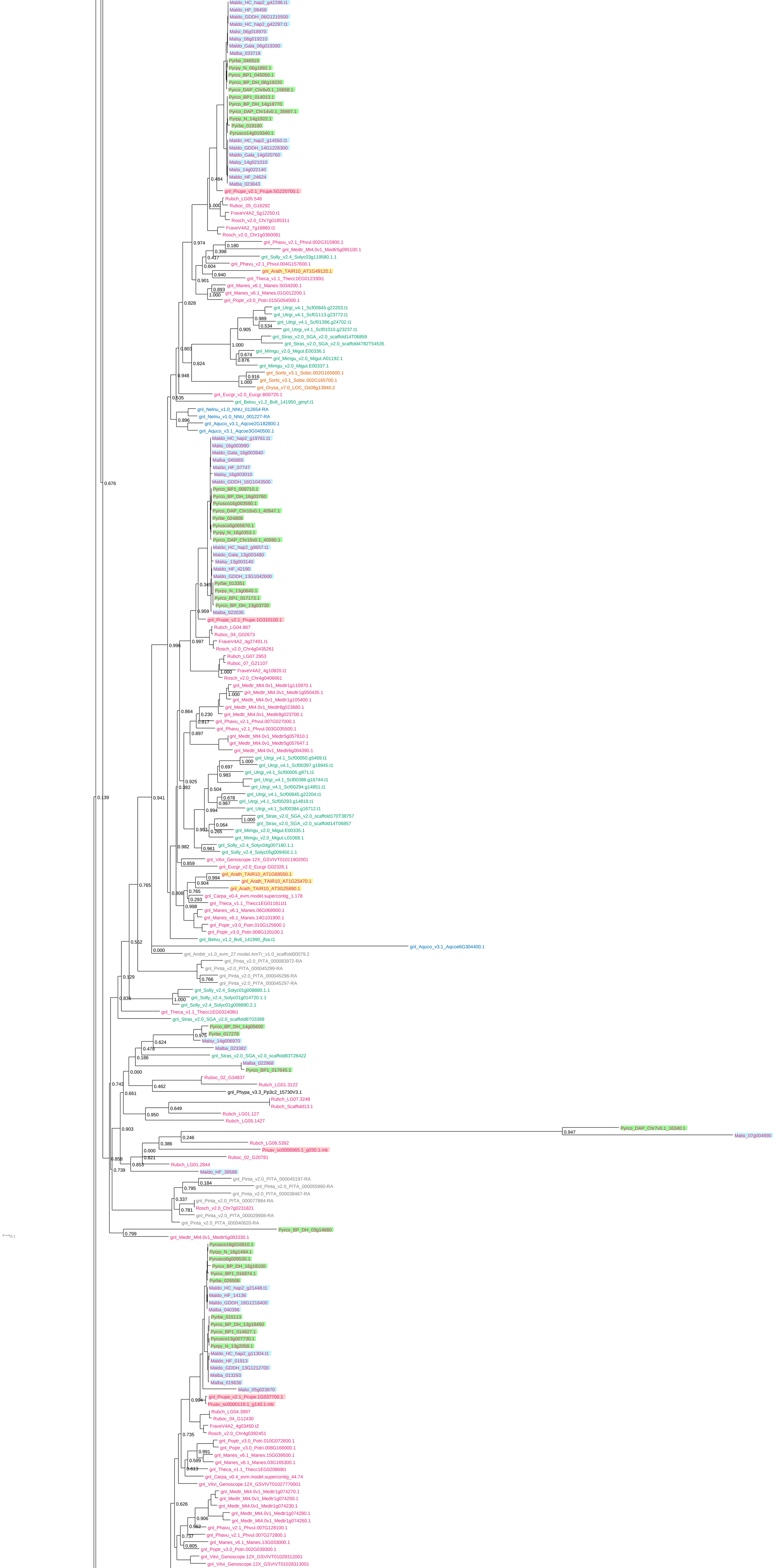

Maldo_HC_hap2_g42296.t1
Maldo_HF_09458
Maldo_GDDH_06G1215500
Maldo_HC_hap2_g42297.t1
Malsi_06g018970
Malsy_06g019210
Maldo_Gala_06g019390
Malba_033718
Pyrbe_046919
Pyrpy_N_06g1892.1
Pyrco_BP1_045050.1
Pyrco_BP_DH_06g19220
Pyrco_DAP_Chr6v0.1_15658.1
Pyrco_BP1_014013.1
Pyrco_BP_DH_14g18770
Pyrco_DAP_Chr14v0.1_35997.1
Pyrpy_N_14g1922.1
Pyrbe_019190
Pyrusco14g019340.1
Maldo_HC_hap2_g14550.t1
Maldo_GDDH_14G1226300
Maldo_Gala_14g020760
Malsy_14g021010
Malsi_14g022140
Maldo_HF_24624
Malba_023843
gnl_Prupe_v2.1_Prupe.5G220700.1
Rubch_LG05.548
Ruboc_05_G16292
FraveV4A2_5g12250.t1
Rosch_v2.0_Chr7g0185311
FraveV4A2_7g16860.t1
Rosch_v2.0_Chr1g0360081
gnl_Phavu_v2.1_Phvul.002G315900.1
gnl_Medtr_Mt4.0v1_Medtr5g095100.1
gnl_Solly_v2.4_Solyc03g119580.1.1
gnl_Phavu_v2.1_Phvul.004G157600.1
gnl_Arath_TAIR10_AT1G49120.1
gnl_Theca_v1.1_Thecc1EG012330t1
gnl_Manes_v6.1_Manes.S034200.1
gnl_Manes_v6.1_Manes.01G012200.1
gnl_Poptr_v3.0_Potri.015G054500.1
gnl_Utrgi_v4.1_Scf00845.g22203.t1
gnl_Utrgi_v4.1_Scf01113.g23772.t1
gnl_Utrgi_v4.1_Scf01396.g24702.t1
gnl_Utrgi_v4.1_Scf01010.g23237.t1
gnl_Stras_v2.0_SGA_v2.0_scaffold14T06659
gnl_Stras_v2.0_SGA_v2.0_scaffold4782T54535
gnl_Mimgu_v2.0_Migut.E00336.1
gnl_Mimgu_v2.0_Migut.A01192.1
gnl_Mimgu_v2.0_Migut.E00337.1
gnl_Sorbi_v3.1_Sobic.002G165600.1
gnl_Sorbi_v3.1_Sobic.002G165700.1
gnl_Orysa_v7.0_LOC_Os09g13940.2
gnl_Eucgr_v2.0_Eucgr.B00720.1
gnl_Betvu_v1.2_Bv6_141950_gmyf.t1
gnl_Nelnu_v1.0_NNU_012654-RA
gnl_Nelnu_v1.0_NNU_001227-RA
gnl_Aquco_v3.1_Aqcoe2G182800.1
gnl_Aquco_v3.1_Aqcoe3G040500.1
Maldo_HC_hap2_g19761.t1
Malsi_16g003990
Maldo_Gala_16g003940
Malba_045665
Maldo_HF_07747
Malsy_16g003010
Maldo_GDDH_16G1043500
Pyrco_BP1_009710.1
Pyrco_BP_DH_15g03760
Pyrusco16g003580.1
Pyrco_DAP_Chr16v0.1_40947.1
Pyrbe_024808
Pyrusco0g065670.1
Pyrpy_N_16g0353.1
Pyrco_DAP_Chr16v0.1_40590.1
Maldo_HC_hap2_g9657.t1
Maldo_Gala_13g003480
Malsy_13g003140
Maldo_HF_42190
Maldo_GDDH_13G1042600
Pyrbe_013351
Pyrpy_N_13g0640.1
Pyrco_BP1_017173.1
Pyrco_BP_DH_13g03730
Malba_022035
gnl_Prupe_v2.1_Prupe.1G310100.1
Rubch_LG04.867
Ruboc_04_G02673
FraveV4A2_4g27491.t1
Rosch_v2.0_Chr4g0435261
Rubch_LG07.2953
Ruboc_07_G21107
FraveV4A2_4g10820.t1
Rosch_v2.0_Chr4g0406661
gnl_Medtr_Mt4.0v1_Medtr1g110970.1
gnl_Medtr_Mt4.0v1_Medtr1g050435.1
gnl_Medtr_Mt4.0v1_Medtr1g105400.1
gnl_Medtr_Mt4.0v1_Medtr8g023680.1
gnl_Medtr_Mt4.0v1_Medtr8g023700.1
gnl_Phavu_v2.1_Phvul.007G027000.1
gnl_Phavu_v2.1_Phvul.003G035500.1
gnl_Medtr_Mt4.0v1_Medtr5g057810.1
gnl_Medtr_Mt4.0v1_Medtr5g057647.1
gnl_Medtr_Mt4.0v1_Medtr6g004390.1
gnl_Utrgi_v4.1_Scf00050.g5469.t1
gnl_Utrgi_v4.1_Scf00397.g16945.t1
gnl_Utrgi_v4.1_Scf00005.g971.t1
gnl_Utrgi_v4.1_Scf00386.g16744.t1
gnl_Utrgi_v4.1_Scf00294.g14851.t1
gnl_Utrgi_v4.1_Scf00845.g22204.t1
gnl_Utrgi_v4.1_Scf00293.g14818.t1
gnl_Utrgi_v4.1_Scf00384.g16712.t1
gnl_Stras_v2.0_SGA_v2.0_scaffold170T38757
gnl_Stras_v2.0_SGA_v2.0_scaffold14T06857
gnl_Mimgu_v2.0_Migut.E00335.1
gnl_Mimgu_v2.0_Migut.L01068.1
gnl_Solly_v2.4_Solyc04g007180.1.1
gnl_Solly_v2.4_Solyc05g009450.1.1
gnl_Vitvi_Genoscope.12X_GSVIVT01011902001
gnl_Eucgr_v2.0_Eucgr.G02326.1
gnl_Arath_TAIR10_AT1G68550.1
gnl_Arath_TAIR10_AT1G25470.1
gnl_Arath_TAIR10_AT3G25890.1
gnl_Carpa_v0.4_evm.model.supercontig_1.178
gnl_Theca_v1.1_Thecc1EG011811t1
gnl_Manes_v6.1_Manes.06G068900.1
gnl_Manes_v6.1_Manes.14G101900.1
gnl_Poptr_v3.0_Potri.010G125600.1
gnl_Poptr_v3.0_Potri.008G120100.1
gnl_Betvu_v1.2_Bv6_141990_jfsa.t1
gnl_Aquco_v3.1_Aqcoe6G304400.1
gnl_Ambtr_v1.0_evm_27.model.AmTr_v1.0_scaffold00079.2
gnl_Pinta_v2.0_PITA_000083972-RA
gnl_Pinta_v2.0_PITA_000045299-RA
gnl_Pinta_v2.0_PITA_000045296-RA
gnl_Pinta_v2.0_PITA_000045297-RA
gnl_Solly_v2.4_Solyc01g008880.1.1
gnl_Solly_v2.4_Solyc01g014720.1.1
gnl_Solly_v2.4_Solyc01g008890.2.1
gnl_Theca_v1.1_Thecc1EG032408t1
gnl_Stras_v2.0_SGA_v2.0_scaffold6T03388
Pyrco_BP_DH_14g05800
Pyrbe_017276
Malsy_14g006970
Malba_023382
gnl_Stras_v2.0_SGA_v2.0_scaffold83T26422
Malba_022968
Pyrco_BP1_017645.1
Ruboc_02_G34837
Rubch_LG01.3122
gnl_Phypa_v3.3_Pp3c2_15730V3.1
Rubch_LG07.3248
Rubch_Scaffold13.1
Rubch_LG01.127
Rubch_LG05.1427
Pyrco_DAP_Chr7v0.1_16340.1
Malsi_07g004990
Rubch_LG06.5392
Pruav_sc0006965.1_g030.1.mk
Ruboc_02_G20781
Rubch_LG01.2844
Maldo_HF_39586
gnl_Pinta_v2.0_PITA_000045197-RA
gnl_Pinta_v2.0_PITA_000055990-RA
gnl_Pinta_v2.0_PITA_000038467-RA
gnl_Pinta_v2.0_PITA_000077884-RA
Rosch_v2.0_Chr7g0231821
gnl_Pinta_v2.0_PITA_000029908-RA
gnl_Pinta_v2.0_PITA_000040620-RA
Pyrco_BP_DH_03g14660
gnl_Medtr_Mt4.0v1_Medtr5g083330.1
Pyrusco16g010910.1
Pyrpy_N_16g1494.1
Pyrusco0g009530.1
Pyrco_BP_DH_16g18100
Pyrco_BP1_016874.1
Pyrbe_026506
Maldo_HC_hap2_g21448.t1
Maldo_HF_14136
Maldo_GDDH_16G1216400
Malba_040396
Pyrbe_015113
Pyrco_BP_DH_13g18450
Pyrco_BP1_014927.1
Pyrusco13g007730.1
Pyrpy_N_13g2058.1
Maldo_HC_hap2_g11304.t1
Maldo_HF_01913
Maldo_GDDH_13G1212700
Malba_013293
Malba_019636
Malsi_05g023670
gnl_Prupe_v2.1_Prupe.1G037700.1
Pruav_sc0000119.1_g140.1.mk
Rubch_LG04.3997
Ruboc_04_G12430
FraveV4A2_4g03450.t2
Rosch_v2.0_Chr4g0392451
gnl_Poptr_v3.0_Potri.010G072600.1
gnl_Poptr_v3.0_Potri.008G166000.1
gnl_Manes_v6.1_Manes.15G039500.1
gnl_Manes_v6.1_Manes.03G165300.1
gnl_Theca_v1.1_Thecc1EG020869t1
gnl_Carpa_v0.4_evm.model.supercontig_44.74
gnl_Vitvi_Genoscope.12X_GSVIVT01027770001
gnl_Medtr_Mt4.0v1_Medtr1g074270.1
gnl_Medtr_Mt4.0v1_Medtr1g074250.1
gnl_Medtr_Mt4.0v1_Medtr1g074230.1
gnl_Medtr_Mt4.0v1_Medtr1g074280.1
gnl_Medtr_Mt4.0v1_Medtr1g074260.1
gnl_Phavu_v2.1_Phvul.007G128100.1
gnl_Phavu_v2.1_Phvul.007G272800.1
gnl_Manes_v6.1_Manes.13G033000.1
gnl_Poptr_v3.0_Potri.002G039300.1
gnl_Vitvi_Genoscope.12X_GSVIVT01028312001
gnl_Vitvi_Genoscope.12X_GSVIVT01028313001
0.2

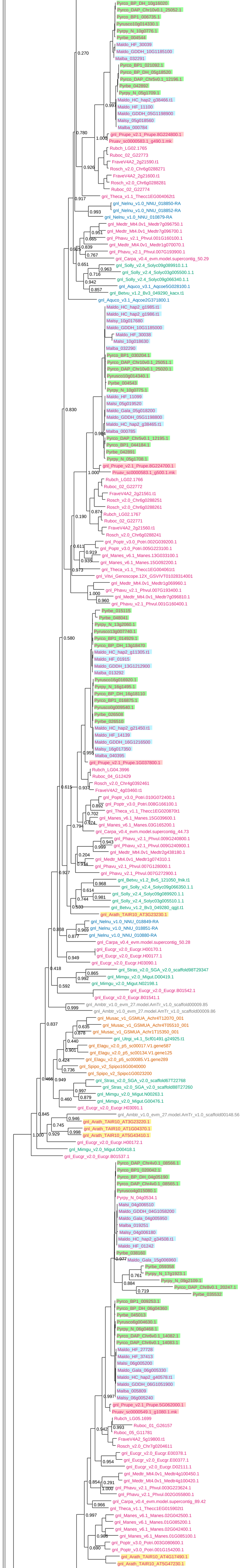

Pyrco_BP_DH_10g16020
Pyrco_DAP_Chr10v0.1_25052.1
Pyrco_BP1_006735.1
Pyrusco10g014330.1
Pyrpy_N_10g0776.1
Pyrbe_004544
Maldo_HF_30039
Maldo_GDDH_10G1185100
Malba_032291
Pyrco_BP1_021092.1
Pyrco_BP_DH_05g18520
Pyrco_DAP_Chr5v0.1_12196.1
Pyrbe_042892
Pyrpy_N_05g1709.1
Maldo_HC_hap2_g38466.t1
Maldo_HF_11100
Maldo_GDDH_05G1198900
Malsy_05g018560
Malba_000784
gnl_Prupe_v2.1_Prupe.8G224800.1
Pruav_sc0000583.1_g490.1.mk
Rubch_LG02.1765
Ruboc_02_G22773
FraveV4A2_2g21590.t1
Rosch_v2.0_Chr6g0288271
FraveV4A2_2g21600.t1
Rosch_v2.0_Chr6g0288281
Ruboc_02_G22774
gnl_Theca_v1.1_Thecc1EG004062t1
gnl_Nelnu_v1.0_NNU_018850-RA
gnl_Nelnu_v1.0_NNU_018852-RA
gnl_Nelnu_v1.0_NNU_010879-RA
gnl_Medtr_Mt4.0v1_Medtr7g096750.1
gnl_Medtr_Mt4.0v1_Medtr7g096700.1
gnl_Phavu_v2.1_Phvul.001G160100.1
gnl_Medtr_Mt4.0v1_Medtr1g070070.1
gnl_Phavu_v2.1_Phvul.007G193900.1
gnl_Carpa_v0.4_evm.model.supercontig_50.29
gnl_Solly_v2.4_Solyc09g089910.1.1
gnl_Solly_v2.4_Solyc03g005500.1.1
gnl_Solly_v2.4_Solyc09g066340.1.1
gnl_Aquco_v3.1_Aqcoe5G028100.1
gnl_Betvu_v1.2_Bv3_049290_kacx.t1
gnl_Aquco_v3.1_Aqcoe2G371800.1
Maldo_HC_hap2_g1985.t1
Maldo_HC_hap2_g1986.t1
Malsy_10g017680
Maldo_GDDH_10G1185000
Maldo_HF_30038
Malsi_10g018630
Malba_032290
Pyrco_BP1_030204.1
Pyrco_DAP_Chr10v0.1_25051.1
Pyrco_DAP_Chr10v0.1_25020.1
Pyrusco10g014340.1
Pyrbe_004543
Pyrpy_N_10g0775.1
Maldo_HF_11099
Malsi_05g019520
Maldo_Gala_05g018200
Maldo_GDDH_05G1198800
Maldo_HC_hap2_g38465.t1
Malba_000785
Pyrco_DAP_Chr5v0.1_12195.1
Pyrco_BP1_044184.1
Pyrbe_042891
Pyrpy_N_05g1708.1
gnl_Prupe_v2.1_Prupe.8G224700.1
Pruav_sc0000583.1_g500.1.mk
Rubch_LG02.1766
Ruboc_02_G22772
FraveV4A2_2g21561.t1
Rosch_v2.0_Chr6g0288251
Rosch_v2.0_Chr6g0288261
Rubch_LG02.1767
Ruboc_02_G22771
FraveV4A2_2g21560.t1
Rosch_v2.0_Chr6g0288241
gnl_Poptr_v3.0_Potri.002G039200.1
gnl_Poptr_v3.0_Potri.005G223100.1
gnl_Manes_v6.1_Manes.13G033100.1
gnl_Manes_v6.1_Manes.15G092200.1
gnl_Theca_v1.1_Thecc1EG004061t1
gnl_Vitvi_Genoscope.12X_GSVIVT01028314001
gnl_Medtr_Mt4.0v1_Medtr1g069960.1
gnl_Phavu_v2.1_Phvul.007G193400.1
gnl_Medtr_Mt4.0v1_Medtr7g096810.1
gnl_Phavu_v2.1_Phvul.001G160400.1
Pyrbe_015115
Pyrbe_048041
Pyrpy_N_13g2060.1
Pyrusco13g007740.1
Pyrco_BP1_014929.1
Pyrco_BP_DH_13g18470
Maldo_HC_hap2_g11305.t1
Maldo_HF_01915
Maldo_GDDH_13G1212900
Malba_013292
Pyrusco16g016920.1
Pyrpy_N_16g1495.1
Pyrco_BP_DH_16g18110
Pyrco_BP1_016875.1
Pyrusco0g009540.1
Pyrbe_026508
Pyrbe_026510
Maldo_HC_hap2_g21450.t1
Maldo_HF_14139
Maldo_GDDH_16G1216500
Malsy_16g017350
Malba_040395
gnl_Prupe_v2.1_Prupe.1G037800.1
Rubch_LG04.3996
Ruboc_04_G12429
Rosch_v2.0_Chr4g0392461
FraveV4A2_4g03460.t1
gnl_Poptr_v3.0_Potri.010G072400.1
gnl_Poptr_v3.0_Potri.008G166100.1
gnl_Theca_v1.1_Thecc1EG020870t1
gnl_Manes_v6.1_Manes.15G039600.1
gnl_Manes_v6.1_Manes.03G165200.1
gnl_Carpa_v0.4_evm.model.supercontig_44.73
gnl_Phavu_v2.1_Phvul.009G240800.1
gnl_Phavu_v2.1_Phvul.009G240900.1
gnl_Medtr_Mt4.0v1_Medtr2g438180.1
gnl_Medtr_Mt4.0v1_Medtr1g074310.1
gnl_Phavu_v2.1_Phvul.007G128000.1
gnl_Phavu_v2.1_Phvul.007G272900.1
gnl_Betvu_v1.2_Bv5_121050_fnik.t1
gnl_Solly_v2.4_Solyc09g066350.1.1
gnl_Solly_v2.4_Solyc09g089920.1.1
gnl_Solly_v2.4_Solyc03g005510.1.1
gnl_Betvu_v1.2_Bv3_049280_qgjt.t1
gnl_Arath_TAIR10_AT3G23230.1
gnl_Nelnu_v1.0_NNU_018849-RA
gnl_Nelnu_v1.0_NNU_018851-RA
gnl_Nelnu_v1.0_NNU_010880-RA
gnl_Carpa_v0.4_evm.model.supercontig_50.28
gnl_Eucgr_v2.0_Eucgr.H00170.1
gnl_Eucgr_v2.0_Eucgr.H00177.1
gnl_Eucgr_v2.0_Eucgr.H03090.1
gnl_Stras_v2.0_SGA_v2.0_scaffold98T29347
gnl_Mimgu_v2.0_Migut.D00419.1
gnl_Mimgu_v2.0_Migut.N02198.1
gnl_Eucgr_v2.0_Eucgr.B01542.1
gnl_Eucgr_v2.0_Eucgr.B01541.1
gnl_Ambtr_v1.0_evm_27.model.AmTr_v1.0_scaffold00009.85
gnl_Ambtr_v1.0_evm_27.model.AmTr_v1.0_scaffold00009.86
gnl_Musac_v1_GSMUA_Achr4T12070_001
gnl_Musac_v1_GSMUA_Achr4T05510_001
gnl_Musac_v1_GSMUA_Achr1T15350_001
gnl_Utrgi_v4.1_Scf01491.g24925.t1
gnl_Elagu_v2.0_p5_sc00017.V1.gene587
gnl_Elagu_v2.0_p5_sc00134.V1.gene125
gnl_Elagu_v2.0_p5_sc00085.V1.gene289
gnl_Spipo_v2_Spipo16G0040000
gnl_Spipo_v2_Spipo1G0023200
gnl_Stras_v2.0_SGA_v2.0_scaffold67T22768
gnl_Stras_v2.0_SGA_v2.0_scaffold88T27260
gnl_Mimgu_v2.0_Migut.N00263.1
gnl_Mimgu_v2.0_Migut.G00476.1
gnl_Eucgr_v2.0_Eucgr.H03091.1
gnl_Ambtr_v1.0_evm_27.model.AmTr_v1.0_scaffold00148.56
gnl_Arath_TAIR10_AT3G23220.1
gnl_Arath_TAIR10_AT1G04370.1
gnl_Arath_TAIR10_AT5G43410.1
gnl_Eucgr_v2.0_Eucgr.H00172.1
gnl_Mimgu_v2.0_Migut.D00418.1
gnl_Eucgr_v2.0_Eucgr.B01537.1
Pyrco_DAP_Chr4v0.1_08566.1
Pyrco_BP1_020042.1
Pyrco_BP_DH_04g05190
Pyrco_DAP_Chr4v0.1_08565.1
Pyrusco4g015080.1
Pyrpy_N_04g0534.1
Malsi_04g006510
Maldo_GDDH_04G1058200
Maldo_Gala_04g005950
Malba_019251
Malsy_04g006180
Maldo_HC_hap2_g34508.t1
Maldo_HF_01242
Pyrbe_038160
Maldo_Gala_15g006960
Pyrbe_059358
Pyrpy_N_17g1923.1
Pyrpy_N_09g2109.1
Pyrco_DAP_Chr8v0.1_20247.1
Pyrbe_035532
Pyrco_BP1_009253.1
Pyrco_BP_DH_06g04360
Pyrbe_045013
Pyrusco6g004630.1
Pyrpy_N_06g0468.1
Pyrco_DAP_Chr6v0.1_14082.1
Pyrco_DAP_Chr6v0.1_14083.1
Maldo_HF_27728
Maldo_HF_37413
Malsi_06g005200
Maldo_Gala_06g005330
Maldo_HC_hap2_g40578.t1
Maldo_GDDH_06G1051900
Malba_005809
Malsy_06g005240
gnl_Prupe_v2.1_Prupe.5G062000.1
Pruav_sc0000549.1_g1080.1.mk
Rubch_LG05.1699
Ruboc_01_G26157
Ruboc_05_G11781
FraveV4A2_5g19800.t1
Rosch_v2.0_Chr7g0204611
gnl_Eucgr_v2.0_Eucgr.E00378.1
gnl_Eucgr_v2.0_Eucgr.E00377.1
gnl_Eucgr_v2.0_Eucgr.D02111.1
gnl_Medtr_Mt4.0v1_Medtr4g100450.1
gnl_Medtr_Mt4.0v1_Medtr4g100420.1
gnl_Phavu_v2.1_Phvul.003G223624.1
gnl_Phavu_v2.1_Phvul.002G055800.1
gnl_Carpa_v0.4_evm.model.supercontig_89.42
gnl_Theca_v1.1_Thecc1EG015902t1
gnl_Manes_v6.1_Manes.02G042500.1
gnl_Manes_v6.1_Manes.01G085200.1
gnl_Manes_v6.1_Manes.02G042400.1
gnl_Manes_v6.1_Manes.01G085100.1
gnl_Poptr_v3.0_Potri.003G080600.1
gnl_Poptr_v3.0_Potri.001G154200.1
gnl_Arath_TAIR10_AT4G17490.1
gnl_Arath_TAIR10_AT5G47230.1

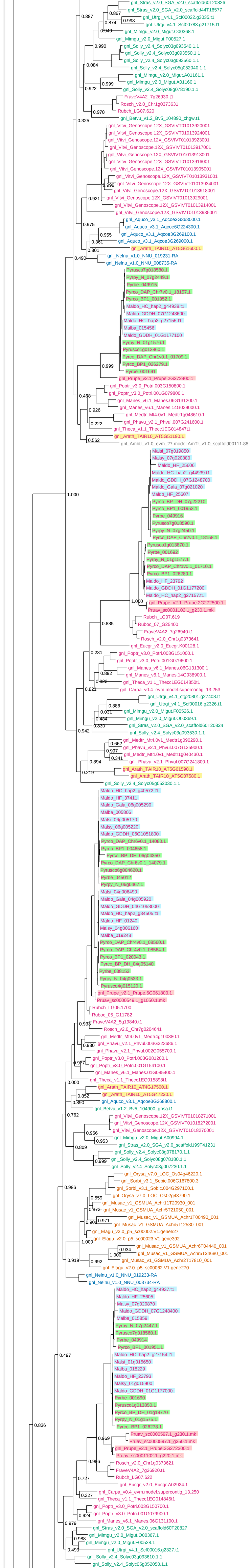
gnl_Stras_v2.0_SGA_v2.0_scaffold60T20826
gnl_Stras_v2.0_SGA_v2.0_scaffold44T16577
gnl_Utrgi_v4.1_Scf00022.g3035.t1
gnl_Utrgi_v4.1_Scf00783.g21715.t1
gnl_Mimgu_v2.0_Migut.O00368.1
gnl_Mimgu_v2.0_Migut.F00527.1
gnl_Solly_v2.4_Solyc03g093540.1.1
gnl_Solly_v2.4_Solyc03g093550.1.1
gnl_Solly_v2.4_Solyc03g093560.1.1
gnl_Solly_v2.4_Solyc05g052040.1.1
gnl_Mimgu_v2.0_Migut.A01161.1
gnl_Mimgu_v2.0_Migut.A01160.1
gnl_Solly_v2.4_Solyc08g078190.1.1
FraveV4A2_7g26930.t1
Rosch_v2.0_Chr1g0373631
Rubch_LG07.620
gnl_Betvu_v1.2_Bv5_104890_chgw.t1
gnl_Vitvi_Genoscope.12X_GSVIVT01013920001
gnl_Vitvi_Genoscope.12X_GSVIVT01013924001
gnl_Vitvi_Genoscope.12X_GSVIVT01013923001
gnl_Vitvi_Genoscope.12X_GSVIVT01013917001
gnl_Vitvi_Genoscope.12X_GSVIVT01013913001
gnl_Vitvi_Genoscope.12X_GSVIVT01013916001
gnl_Vitvi_Genoscope.12X_GSVIVT01013905001
gnl_Vitvi_Genoscope.12X_GSVIVT01013931001
gnl_Vitvi_Genoscope.12X_GSVIVT01013934001
gnl_Vitvi_Genoscope.12X_GSVIVT01013918001
gnl_Vitvi_Genoscope.12X_GSVIVT01013929001
gnl_Vitvi_Genoscope.12X_GSVIVT01013914001
gnl_Vitvi_Genoscope.12X_GSVIVT01013935001
gnl_Aquco_v3.1_Aqcoe2G363000.1
gnl_Aquco_v3.1_Aqcoe6G224300.1
gnl_Aquco_v3.1_Aqcoe3G269100.1
gnl_Aquco_v3.1_Aqcoe3G269000.1
gnl_Arath_TAIR10_AT5G61600.1
gnl_Nelnu_v1.0_NNU_019231-RA
gnl_Nelnu_v1.0_NNU_008735-RA
Pyrusco7g018580.1
Pyrpy_N_07g2449.1
Pyrbe_049915
Pyrco_DAP_Chr7v0.1_18157.1
Pyrco_BP1_001952.1
Maldo_HC_hap2_g44938.t1
Maldo_GDDH_07G1248600
Maldo_HC_hap2_g27155.t1
Malba_015456
Maldo_GDDH_01G1177100
Pyrpy_N_01g1576.1
Pyrusco1g013860.1
Pyrco_DAP_Chr1v0.1_01709.1
Pyrco_BP1_026279.1
Pyrbe_001691
gnl_Prupe_v2.1_Prupe.2G272400.1
gnl_Poptr_v3.0_Potri.003G150800.1
gnl_Poptr_v3.0_Potri.001G079800.1
gnl_Manes_v6.1_Manes.06G131200.1
gnl_Manes_v6.1_Manes.14G039000.1
gnl_Medtr_Mt4.0v1_Medtr1g048610.1
gnl_Phavu_v2.1_Phvul.007G241600.1
gnl_Theca_v1.1_Thecc1EG014847t1
gnl_Arath_TAIR10_AT5G51190.1
gnl_Ambtr_v1.0_evm_27.model.AmTr_v1.0_scaffold00111.88
Malsi_07g019850
Malsy_07g020880
Maldo_HF_25606
Maldo_HC_hap2_g44939.t1
Maldo_GDDH_07G1248700
Maldo_Gala_07g021020
Maldo_HF_25607
Pyrco_BP_DH_07g22210
Pyrco_BP1_001953.1
Pyrbe_049916
Pyrusco7g018590.1
Pyrpy_N_07g2450.1
Pyrco_DAP_Chr7v0.1_18158.1
Pyrusco1g013870.1
Pyrbe_001692
Pyrpy_N_01g1577.1
Pyrco_DAP_Chr1v0.1_01710.1
Pyrco_BP1_026280.1
Maldo_HF_23792
Maldo_GDDH_01G1177200
Maldo_HC_hap2_g27157.t1
gnl_Prupe_v2.1_Prupe.2G272500.1
Pruav_sc0001102.1_g230.1.mk
Rubch_LG07.619
Ruboc_07_G25400
FraveV4A2_7g26940.t1
Rosch_v2.0_Chr1g0373641
gnl_Eucgr_v2.0_Eucgr.K00128.1
gnl_Poptr_v3.0_Potri.003G151000.1
gnl_Poptr_v3.0_Potri.001G079600.1
gnl_Manes_v6.1_Manes.06G131300.1
gnl_Manes_v6.1_Manes.14G038900.1
gnl_Theca_v1.1_Thecc1EG014850t1
gnl_Carpa_v0.4_evm.model.supercontig_13.253
gnl_Utrgi_v4.1_ctg20801.g27408.t1
gnl_Utrgi_v4.1_Scf00016.g2326.t1
gnl_Mimgu_v2.0_Migut.F00526.1
gnl_Mimgu_v2.0_Migut.O00369.1
gnl_Stras_v2.0_SGA_v2.0_scaffold60T20824
gnl_Solly_v2.4_Solyc03g093530.1.1
gnl_Medtr_Mt4.0v1_Medtr1g090290.1
gnl_Phavu_v2.1_Phvul.007G135900.1
gnl_Medtr_Mt4.0v1_Medtr1g040430.1
gnl_Phavu_v2.1_Phvul.007G241800.1
gnl_Arath_TAIR10_AT5G61590.1
gnl_Arath_TAIR10_AT5G07580.1
gnl_Solly_v2.4_Solyc05g052030.1.1
Maldo_HC_hap2_g40572.t1
Maldo_HF_37411
Maldo_Gala_06g005290
Malba_005806
Malsi_06g005170
Malsy_06g005220
Maldo_GDDH_06G1051800
Pyrco_DAP_Chr6v0.1_14080.1
Pyrco_BP1_004658.1
Pyrco_BP_DH_06g04350
Pyrco_DAP_Chr6v0.1_14079.1
Pyrusco6g004620.1
Pyrbe_045012
Pyrpy_N_06g0467.1
Malsi_04g006490
Maldo_Gala_04g005920
Maldo_GDDH_04G1058000
Maldo_HC_hap2_g34505.t1
Maldo_HF_01240
Malsy_04g006160
Malba_019248
Pyrco_DAP_Chr4v0.1_08560.1
Pyrco_DAP_Chr4v0.1_08564.1
Pyrco_BP1_020043.1
Pyrco_BP_DH_04g05140
Pyrbe_038153
Pyrpy_N_04g0533.1
Pyrusco4g015120.1
gnl_Prupe_v2.1_Prupe.5G061800.1
Pruav_sc0000549.1_g1050.1.mk
Rubch_LG05.1700
Ruboc_05_G11782
FraveV4A2_5g19840.t1
Rosch_v2.0_Chr7g0204641
gnl_Medtr_Mt4.0v1_Medtr4g100380.1
gnl_Phavu_v2.1_Phvul.003G223686.1
gnl_Phavu_v2.1_Phvul.002G055700.1
gnl_Poptr_v3.0_Potri.003G081200.1
gnl_Poptr_v3.0_Potri.001G154100.1
gnl_Manes_v6.1_Manes.01G085400.1
gnl_Theca_v1.1_Thecc1EG015898t1
gnl_Arath_TAIR10_AT4G17500.1
gnl_Arath_TAIR10_AT5G47220.1
gnl_Aquco_v3.1_Aqcoe3G268800.1
gnl_Betvu_v1.2_Bv5_104900_ghsa.t1
gnl_Vitvi_Genoscope.12X_GSVIVT01018271001
gnl_Vitvi_Genoscope.12X_GSVIVT01018272001
gnl_Vitvi_Genoscope.12X_GSVIVT01018270001
gnl_Mimgu_v2.0_Migut.A00994.1
gnl_Stras_v2.0_SGA_v2.0_scaffold199T41231
gnl_Solly_v2.4_Solyc08g078170.1.1
gnl_Solly_v2.4_Solyc08g078180.1.1
gnl_Solly_v2.4_Solyc08g007230.1.1
gnl_Orysa_v7.0_LOC_Os04g46220.1
gnl_Sorbi_v3.1_Sobic.006G167800.3
gnl_Sorbi_v3.1_Sobic.004G297100.1
gnl_Orysa_v7.0_LOC_Os02g43790.1
gnl_Musac_v1_GSMUA_Achr11T20930_001
gnl_Musac_v1_GSMUA_Achr5T21050_001
gnl_Musac_v1_GSMUA_Achr1T00490_001
gnl_Musac_v1_GSMUA_Achr5T12530_001
gnl_Elagu_v2.0_p5_sc00002.V1.gene527
gnl_Elagu_v2.0_p5_sc00023.V1.gene392
gnl_Musac_v1_GSMUA_Achr6T04440_001
gnl_Musac_v1_GSMUA_Achr5T24680_001
gnl_Musac_v1_GSMUA_Achr2T17810_001
gnl_Elagu_v2.0_p5_sc00062.V1.gene270
gnl_Nelnu_v1.0_NNU_019233-RA
gnl_Nelnu_v1.0_NNU_008734-RA
Maldo_HC_hap2_g44937.t1
Maldo_HF_25605
Malsy_07g020870
Maldo_GDDH_07G1248400
Malba_015859
Pyrpy_N_07g2447.1
Pyrusco7g018560.1
Pyrbe_049914
Pyrco_BP1_001951.1
Maldo_HC_hap2_g27154.t1
Malsi_01g015650
Malba_018229
Maldo_HF_23793
Malsy_01g015900
Maldo_GDDH_01G1177000
Pyrbe_001690
Pyrusco1g013850.1
Pyrco_BP_DH_01g18770
Pyrpy_N_01g1575.1
Pyrco_BP1_026278.1
Pruav_sc0000597.1_g230.1.mk
Pruav_sc0000597.1_g250.1.mk
gnl_Prupe_v2.1_Prupe.2G272300.1
Pruav_sc0001102.1_g220.1.mk
Rosch_v2.0_Chr1g0373621
FraveV4A2_7g26920.t1
Rubch_LG07.622
gnl_Eucgr_v2.0_Eucgr.A02924.1
gnl_Carpa_v0.4_evm.model.supercontig_13.250
gnl_Theca_v1.1_Thecc1EG014845t1
gnl_Poptr_v3.0_Potri.003G150700.1
gnl_Poptr_v3.0_Potri.001G079900.1
gnl_Manes_v6.1_Manes.06G131100.1
gnl_Stras_v2.0_SGA_v2.0_scaffold60T20827
gnl_Mimgu_v2.0_Migut.O00367.1
gnl_Mimgu_v2.0_Migut.F00528.1
gnl_Utrgi_v4.1_Scf00016.g2327.t1
gnl_Solly_v2.4_Solyc03g093610.1.1
gnl_Solly_v2.4_Solyc05g052050.1.1

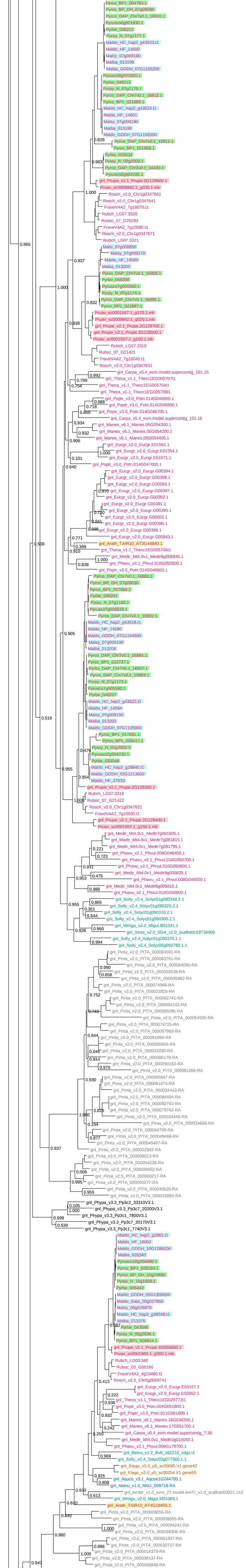

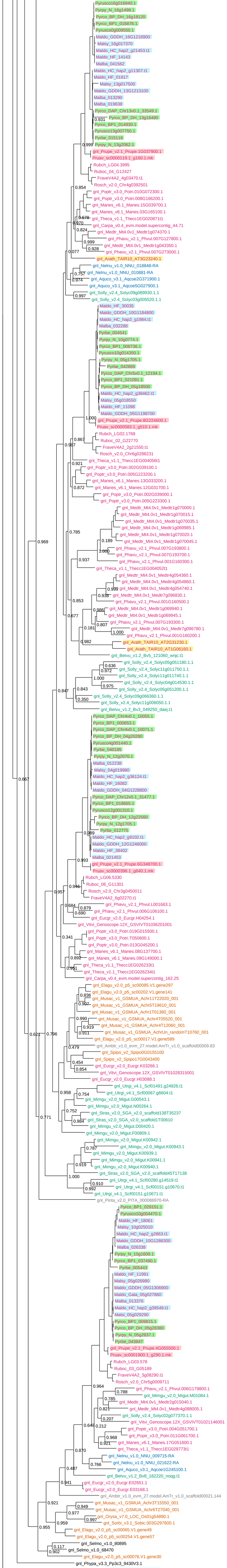

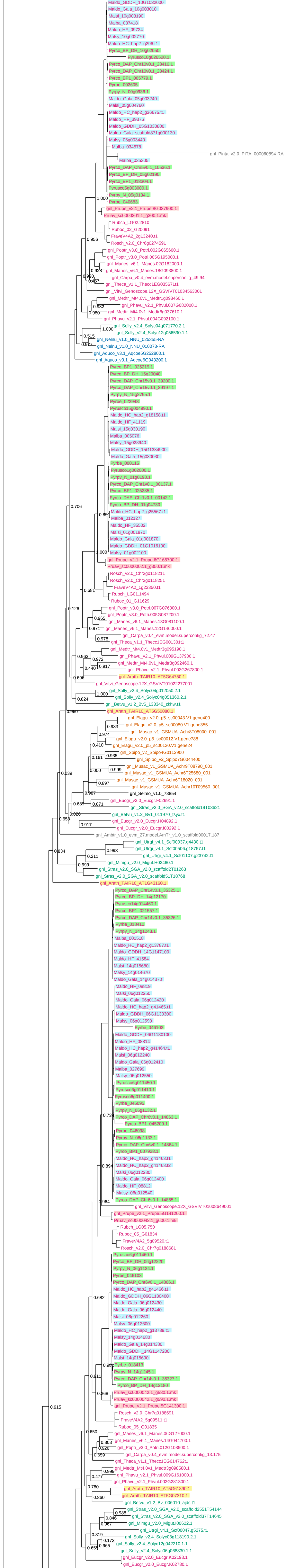

Maldo_GDDH_10G1032000
Maldo_Gala_10g003010
Malsi_10g003190
Malba_037418
Maldo_HF_09724
Malsy_10g002770
Maldo_HC_hap2_g296.t1
Pyrco_BP_DH_10g02050
Pyrusco10g026520.1
Pyrco_DAP_Chr10v0.1_23418.1
Pyrco_DAP_Chr10v0.1_23424.1
Pyrco_BP1_005779.1
Pyrbe_002605
Pyrpy_N_00g0936.1
Maldo_Gala_05g003240
Malsi_05g004760
Maldo_HC_hap2_g36675.t1
Maldo_HF_39376
Maldo_GDDH_05G1030800
Maldo_Gala_scaffold571g000130
Malsy_05g003440
Malba_034578
gnl_Pinta_v2.0_PITA_000060894-RA
Malba_035305
Pyrco_DAP_Chr5v0.1_10536.1
Pyrco_BP_DH_05g02590
Pyrco_BP1_018304.1
Pyrusco5g003000.1
Pyrpy_N_05g0134.1
Pyrbe_040683
gnl_Prupe_v2.1_Prupe.8G037900.1
Pruav_sc0000201.1_g300.1.mk
Rubch_LG02.2810
Ruboc_02_G20091
FraveV4A2_2g13240.t1
Rosch_v2.0_Chr6g0274591
gnl_Poptr_v3.0_Potri.002G065600.1
gnl_Poptr_v3.0_Potri.005G195000.1
gnl_Manes_v6.1_Manes.02G182000.1
gnl_Manes_v6.1_Manes.18G093800.1
gnl_Carpa_v0.4_evm.model.supercontig_49.94
gnl_Theca_v1.1_Thecc1EG035671t1
gnl_Vitvi_Genoscope.12X_GSVIVT01034563001
gnl_Medtr_Mt4.0v1_Medtr1g098460.1
gnl_Phavu_v2.1_Phvul.007G082000.1
gnl_Medtr_Mt4.0v1_Medtr6g037610.1
gnl_Phavu_v2.1_Phvul.004G092100.1
gnl_Solly_v2.4_Solyc04g071770.2.1
gnl_Solly_v2.4_Solyc12g056590.1.1
gnl_Nelnu_v1.0_NNU_025355-RA
gnl_Nelnu_v1.0_NNU_010073-RA
gnl_Aquco_v3.1_Aqcoe5G252800.1
gnl_Aquco_v3.1_Aqcoe6G043200.1
Pyrco_BP1_025219.1
Pyrco_BP_DH_15g29040
Pyrco_DAP_Chr15v0.1_39200.1
Pyrco_DAP_Chr15v0.1_39197.1
Pyrpy_N_15g2795.1
Pyrbe_022943
Pyrusco15g004990.1
Maldo_HC_hap2_g18158.t1
Maldo_HF_41119
Malsi_15g030190
Malba_005076
Malsy_15g028940
Maldo_GDDH_15G1334900
Maldo_Gala_15g030030
Pyrbe_000115
Pyrusco1g002000.1
Pyrpy_N_01g0190.1
Pyrco_DAP_Chr1v0.1_00137.1
Pyrco_BP1_025235.1
Pyrco_DAP_Chr1v0.1_00142.1
Pyrco_BP_DH_01g04730
Maldo_HC_hap2_g25567.t1
Malba_012127
Maldo_HF_35502
Malsi_01g001870
Maldo_Gala_01g001870
Maldo_GDDH_01G1016100
Malsy_01g002100
gnl_Prupe_v2.1_Prupe.6G155700.1
Pruav_sc0000002.1_g350.1.mk
Rosch_v2.0_Chr2g0118211
Rosch_v2.0_Chr2g0118251
FraveV4A2_1g23350.t1
Rubch_LG01.1494
Ruboc_01_G11629
gnl_Poptr_v3.0_Potri.007G076800.1
gnl_Poptr_v3.0_Potri.005G087200.1
gnl_Manes_v6.1_Manes.13G081100.1
gnl_Manes_v6.1_Manes.12G146000.1
gnl_Carpa_v0.4_evm.model.supercontig_72.47
gnl_Theca_v1.1_Thecc1EG001301t1
gnl_Medtr_Mt4.0v1_Medtr3g095190.1
gnl_Phavu_v2.1_Phvul.009G137900.1
gnl_Medtr_Mt4.0v1_Medtr8g092460.1
gnl_Phavu_v2.1_Phvul.002G267800.1
gnl_Arath_TAIR10_AT5G64750.1
gnl_Vitvi_Genoscope.12X_GSVIVT01022277001
gnl_Solly_v2.4_Solyc04g012050.2.1
gnl_Solly_v2.4_Solyc04g051360.2.1
gnl_Betvu_v1.2_Bv6_133340_zkhw.t1
gnl_Arath_TAIR10_AT5G50080.1
gnl_Elagu_v2.0_p5_sc00043.V1.gene400
gnl_Elagu_v2.0_p5_sc00080.V1.gene355
gnl_Musac_v1_GSMUA_Achr8T08000_001
gnl_Elagu_v2.0_p5_sc00012.V1.gene788
gnl_Elagu_v2.0_p5_sc00120.V1.gene24
gnl_Spipo_v2_Spipo4G0112900
gnl_Spipo_v2_Spipo7G0044400
gnl_Musac_v1_GSMUA_Achr9T08790_001
gnl_Musac_v1_GSMUA_Achr6T25680_001
gnl_Musac_v1_GSMUA_Achr6T18020_001
gnl_Musac_v1_GSMUA_Achr10T09560_001
gnl_Selmo_v1.0_73854
gnl_Eucgr_v2.0_Eucgr.F02691.1
gnl_Stras_v2.0_SGA_v2.0_scaffold19T08621
gnl_Betvu_v1.2_Bv1_011970_tsyx.t1
gnl_Eucgr_v2.0_Eucgr.H04892.1
gnl_Eucgr_v2.0_Eucgr.I00292.1
gnl_Ambtr_v1.0_evm_27.model.AmTr_v1.0_scaffold00017.187
gnl_Utrgi_v4.1_Scf00037.g4430.t1
gnl_Utrgi_v4.1_Scf00506.g18757.t1
gnl_Utrgi_v4.1_Scf01107.g23742.t1
gnl_Mimgu_v2.0_Migut.H02460.1
gnl_Stras_v2.0_SGA_v2.0_scaffold2T01263
gnl_Stras_v2.0_SGA_v2.0_scaffold51T18768
gnl_Arath_TAIR10_AT1G43160.1
Pyrco_DAP_Chr14v0.1_35325.1
Pyrco_BP_DH_14g12170
Pyrusco14g014460.1
Pyrco_BP1_021557.1
Pyrco_DAP_Chr14v0.1_35326.1
Pyrbe_018410
Pyrpy_N_14g1243.1
Malba_001518
Maldo_HC_hap2_g13787.t1
Maldo_GDDH_14G1147100
Maldo_HF_41584
Malsi_14g015680
Malsy_14g014670
Maldo_Gala_14g014370
Maldo_HF_08819
Malsi_06g012250
Maldo_Gala_06g012420
Maldo_HC_hap2_g41465.t1
Maldo_GDDH_06G1130300
Malsy_06g012590
Pyrbe_046102
Maldo_GDDH_06G1130100
Maldo_HF_08814
Maldo_HC_hap2_g41464.t1
Malsi_06g012240
Maldo_Gala_06g012410
Malba_027699
Malsy_06g012550
Pyrusco6g011450.1
Pyrusco6g011410.1
Pyrusco6g011400.1
Pyrbe_046095
Pyrpy_N_06g1132.1
Pyrco_DAP_Chr6v0.1_14863.1
Pyrco_BP1_045209.1
Pyrbe_046098
Pyrpy_N_06g1133.1
Pyrco_DAP_Chr6v0.1_14864.1
Pyrco_BP1_007928.1
Maldo_HC_hap2_g41463.t1
Maldo_HC_hap2_g41463.t2
Malsi_06g012230
Maldo_Gala_06g012400
Maldo_HF_08812
Malsy_06g012540
Pyrco_DAP_Chr6v0.1_14865.1
gnl_Vitvi_Genoscope.12X_GSVIVT01008649001
gnl_Prupe_v2.1_Prupe.5G141200.1
Pruav_sc0000042.1_g600.1.mk
Rubch_LG05.750
Ruboc_05_G01834
FraveV4A2_5g09520.t1
Rosch_v2.0_Chr7g0188681
Pyrusco6g011460.1
Pyrco_BP_DH_06g12220
Pyrpy_N_06g1134.1
Pyrbe_046103
Pyrco_DAP_Chr6v0.1_14866.1
Maldo_HC_hap2_g41466.t1
Maldo_GDDH_06G1130400
Maldo_Gala_06g012430
Maldo_Gala_06g012440
Malsi_06g012260
Malsy_06g012600
Maldo_HC_hap2_g13789.t1
Malsy_14g014680
Maldo_Gala_14g014380
Maldo_GDDH_14G1147200
Malsi_14g015690
Pyrbe_018413
Pyrpy_N_14g1245.1
Pyrco_DAP_Chr14v0.1_35327.1
Pyrco_BP_DH_14g12180
Pruav_sc0000042.1_g580.1.mk
Pruav_sc0000042.1_g590.1.mk
gnl_Prupe_v2.1_Prupe.5G141300.1
Rosch_v2.0_Chr7g0188691
FraveV4A2_5g09511.t1
Ruboc_05_G01835
gnl_Manes_v6.1_Manes.06G127000.1
gnl_Manes_v6.1_Manes.14G044700.1
gnl_Poptr_v3.0_Potri.012G108500.1
gnl_Carpa_v0.4_evm.model.supercontig_13.175
gnl_Theca_v1.1_Thecc1EG014762t1
gnl_Medtr_Mt4.0v1_Medtr3g098580.1
gnl_Phavu_v2.1_Phvul.009G161000.1
gnl_Phavu_v2.1_Phvul.002G281300.1
gnl_Arath_TAIR10_AT5G61890.1
gnl_Arath_TAIR10_AT5G07310.1
gnl_Betvu_v1.2_Bv_006010_ajds.t1
gnl_Stras_v2.0_SGA_v2.0_scaffold2551T54144
gnl_Stras_v2.0_SGA_v2.0_scaffold37T14645
gnl_Mimgu_v2.0_Migut.I00622.1
gnl_Utrgi_v4.1_Scf00047.g5275.t1
gnl_Solly_v2.4_Solyc03g118190.2.1
gnl_Solly_v2.4_Solyc12g042210.1.1
gnl_Solly_v2.4_Solyc06g068830.1.1
gnl_Eucgr_v2.0_Eucgr.K02193.1
gnl_Eucgr_v2.0_Eucgr.K02780.1

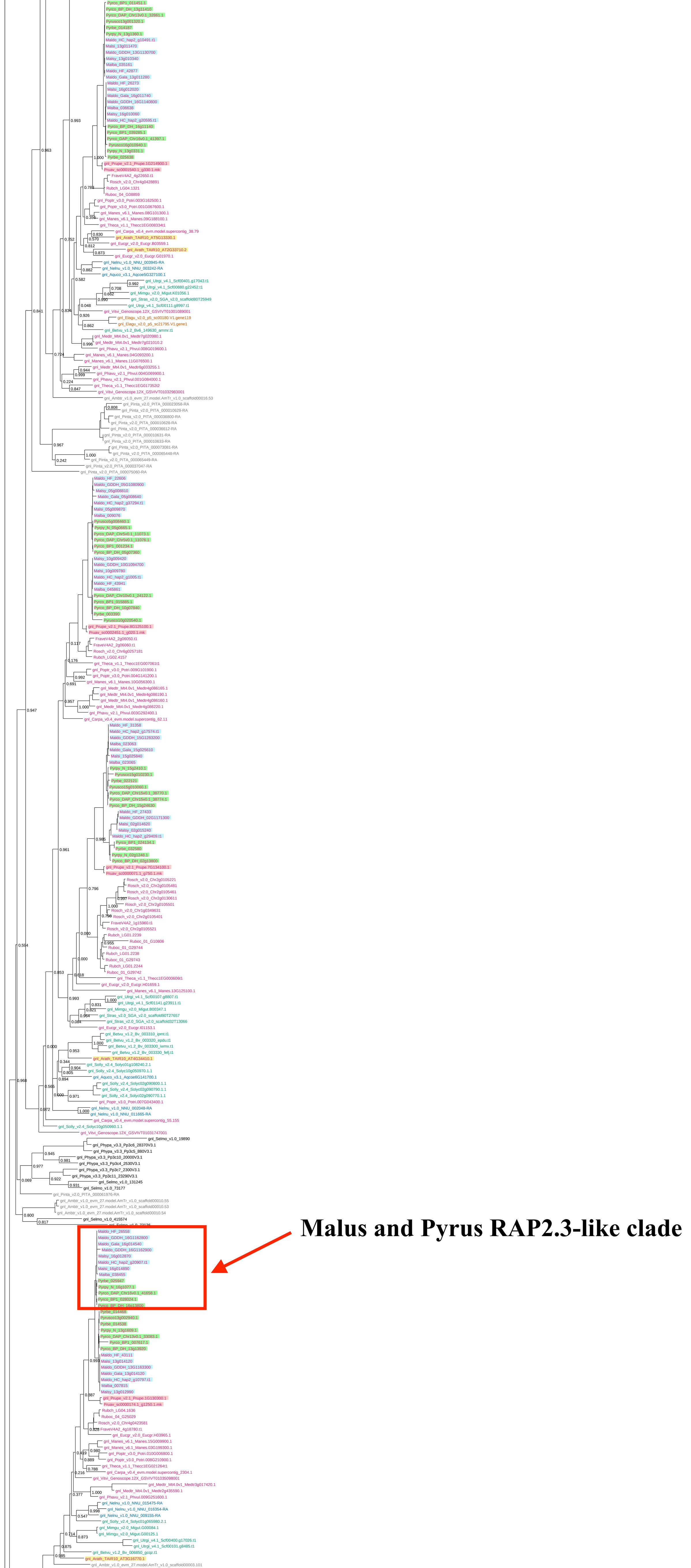
Malus and Pyrus RAP2.3-like clade

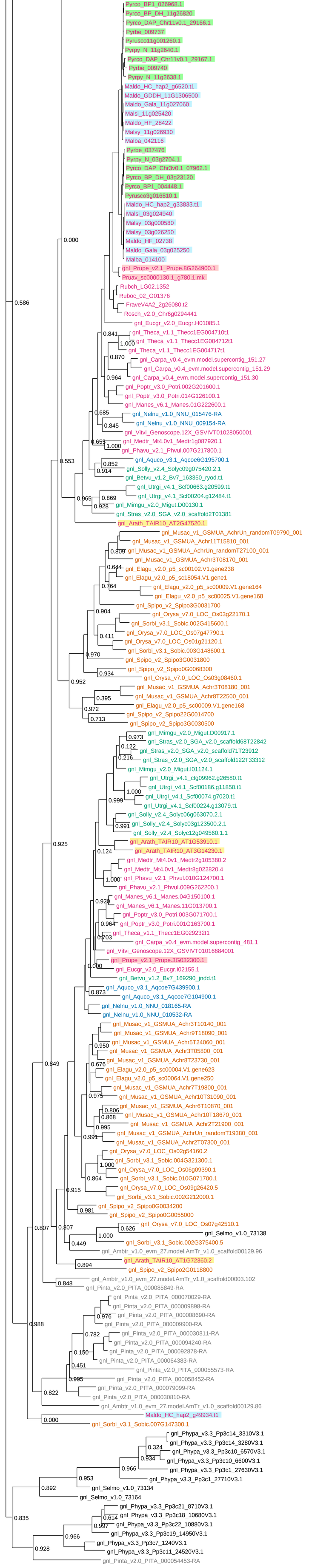
Pyrco_BP1_026968.1
Pyrco_BP_DH_11g26820
Pyrco_DAP_Chr11v0.1_29166.1
Pyrbe_009737
Pyrusco11g001260.1
Pyrpy_N_11g2640.1
Pyrco_DAP_Chr11v0.1_29167.1
Pyrbe_009740
Pyrpy_N_11g2638.1
Maldo_HC_hap2_g6520.t1
Maldo_GDDH_11G1306500
Maldo_Gala_11g027060
Malsi_11g025420
Maldo_HF_28422
Malsy_11g026930
Malba_042116
Pyrbe_037476
Pyrpy_N_03g2704.1
Pyrco_DAP_Chr3v0.1_07962.1
Pyrco_BP_DH_03g23120
Pyrco_BP1_004448.1
Pyrusco3g016810.1
Maldo_HC_hap2_g33833.t1
Malsi_03g024940
Malsy_03g000580
Malsy_03g026250
0.000
Maldo_HF_02738
Maldo_Gala_03g025250
Malba_014100
gnl_Prupe_v2.1_Prupe.8G264900.1
Pruav_sc0000130.1_g780.1.mk
0.586
Rubch_LG02.1352
Ruboc_02_G01376
FraveV4A2_2g26080.t2
Rosch_v2.0_Chr6g0294441
gnl_Eucgr_v2.0_Eucgr.H01085.1
0.841
gnl_Theca_v1.1_Thecc1EG004710t1
1.000
gnl_Theca_v1.1_Thecc1EG004712t1
gnl_Theca_v1.1_Thecc1EG004717t1
0.870
gnl_Carpa_v0.4_evm.model.supercontig_151.27
gnl_Carpa_v0.4_evm.model.supercontig_151.29
0.964
gnl_Carpa_v0.4_evm.model.supercontig_151.30
gnl_Poptr_v3.0_Potri.002G201600.1
gnl_Poptr_v3.0_Potri.014G126100.1
gnl_Manes_v6.1_Manes.01G222600.1
0.685
gnl_Nelnu_v1.0_NNU_015476-RA
0.845
gnl_Nelnu_v1.0_NNU_009154-RA
gnl_Vitvi_Genoscope.12X_GSVIVT01028050001
0.655
gnl_Medtr_Mt4.0v1_Medtr1g087920.1
1.000
gnl_Phavu_v2.1_Phvul.007G217800.1
0.553
gnl_Aquco_v3.1_Aqcoe6G195700.1
0.852
0.914
gnl_Solly_v2.4_Solyc09g075420.2.1
gnl_Betvu_v1.2_Bv7_163350_ryod.t1
gnl_Utrgi_v4.1_Scf00663.g20599.t1
gnl_Utrgi_v4.1_Scf00204.g12484.t1
0.965
0.869
gnl_Mimgu_v2.0_Migut.D00130.1
0.928
gnl_Stras_v2.0_SGA_v2.0_scaffold2T01381
gnl_Arath_TAIR10_AT2G47520.1
gnl_Musac_v1_GSMUA_AchrUn_randomT09790_001
gnl_Musac_v1_GSMUA_Achr11T15810_001
0.809
gnl_Musac_v1_GSMUA_AchrUn_randomT27100_001
gnl_Musac_v1_GSMUA_Achr3T08170_001
0.644
gnl_Elagu_v2.0_p5_sc00102.V1.gene238
gnl_Elagu_v2.0_p5_sc18054.V1.gene1
0.764
gnl_Elagu_v2.0_p5_sc00009.V1.gene164
gnl_Elagu_v2.0_p5_sc00025.V1.gene168
gnl_Spipo_v2_Spipo3G0031700
0.904
gnl_Orysa_v7.0_LOC_Os03g22170.1
gnl_Sorbi_v3.1_Sobic.002G415600.1
gnl_Orysa_v7.0_LOC_Os07g47790.1
0.411
gnl_Orysa_v7.0_LOC_Os01g21120.1
gnl_Sorbi_v3.1_Sobic.003G148600.1
0.970
gnl_Spipo_v2_Spipo3G0031800
gnl_Spipo_v2_Spipo0G0068300
0.934
gnl_Orysa_v7.0_LOC_Os03g08460.1
0.952
gnl_Musac_v1_GSMUA_Achr3T08180_001
gnl_Musac_v1_GSMUA_Achr8T22500_001
0.395
gnl_Elagu_v2.0_p5_sc00009.V1.gene168
0.972
gnl_Spipo_v2_Spipo22G0014700
0.713
gnl_Spipo_v2_Spipo3G0030500
gnl_Mimgu_v2.0_Migut.D00917.1
0.973
gnl_Stras_v2.0_SGA_v2.0_scaffold68T22842
0.122
gnl_Stras_v2.0_SGA_v2.0_scaffold71T23912
0.216
gnl_Stras_v2.0_SGA_v2.0_scaffold122T33312
gnl_Mimgu_v2.0_Migut.I01124.1
gnl_Utrgi_v4.1_ctg09962.g26580.t1
gnl_Utrgi_v4.1_Scf00186.g11850.t1
1.000
gnl_Utrgi_v4.1_Scf00074.g7020.t1
0.999
gnl_Utrgi_v4.1_Scf00224.g13079.t1
gnl_Solly_v2.4_Solyc06g063070.2.1
0.991
gnl_Solly_v2.4_Solyc03g123500.2.1
gnl_Solly_v2.4_Solyc12g049560.1.1
0.925
gnl_Arath_TAIR10_AT1G53910.1
0.124
gnl_Arath_TAIR10_AT3G14230.1
gnl_Medtr_Mt4.0v1_Medtr2g105380.2
gnl_Medtr_Mt4.0v1_Medtr8g022820.4
1.000
gnl_Phavu_v2.1_Phvul.010G124700.1
gnl_Phavu_v2.1_Phvul.009G262200.1
gnl_Manes_v6.1_Manes.04G150100.1
0.920
gnl_Manes_v6.1_Manes.11G013700.1
gnl_Poptr_v3.0_Potri.003G071700.1
0.964
gnl_Poptr_v3.0_Potri.001G163700.1
gnl_Theca_v1.1_Thecc1EG029232t1
0.703
gnl_Carpa_v0.4_evm.model.supercontig_481.1
gnl_Vitvi_Genoscope.12X_GSVIVT01016684001
gnl_Prupe_v2.1_Prupe.3G032300.1
0.000
gnl_Eucgr_v2.0_Eucgr.I02155.1
gnl_Betvu_v1.2_Bv7_169290_jndd.t1
gnl_Aquco_v3.1_Aqcoe7G439900.1
0.873
gnl_Aquco_v3.1_Aqcoe7G104900.1
gnl_Nelnu_v1.0_NNU_018165-RA
gnl_Nelnu_v1.0_NNU_010532-RA
gnl_Musac_v1_GSMUA_Achr3T10140_001
gnl_Musac_v1_GSMUA_Achr9T18090_001
gnl_Musac_v1_GSMUA_Achr5T24060_001
0.950
gnl_Musac_v1_GSMUA_Achr3T05800_001
0.849
gnl_Musac_v1_GSMUA_Achr8T23730_001
0.676
gnl_Elagu_v2.0_p5_sc00004.V1.gene623
gnl_Elagu_v2.0_p5_sc00064.V1.gene250
gnl_Musac_v1_GSMUA_Achr7T19800_001
0.975
gnl_Musac_v1_GSMUA_Achr10T31090_001
gnl_Musac_v1_GSMUA_Achr6T10870_001
0.806
gnl_Musac_v1_GSMUA_Achr10T18670_001
0.868
gnl_Musac_v1_GSMUA_Achr2T21900_001
0.995
gnl_Musac_v1_GSMUA_AchrUn_randomT19380_001
0.991
gnl_Musac_v1_GSMUA_Achr2T07300_001
gnl_Orysa_v7.0_LOC_Os02g54160.2
gnl_Sorbi_v3.1_Sobic.004G321300.1
1.000
gnl_Orysa_v7.0_LOC_Os06g09390.1
0.864
gnl_Sorbi_v3.1_Sobic.010G071700.1
0.915
gnl_Orysa_v7.0_LOC_Os09g26420.5
gnl_Sorbi_v3.1_Sobic.002G212000.1
gnl_Spipo_v2_Spipo0G0034200
0.981
gnl_Spipo_v2_Spipo0G0055000
0.807
0.807
gnl_Orysa_v7.0_LOC_Os07g42510.1
0.626
gnl_Selmo_v1.0_73138
1.000
0.449
gnl_Sorbi_v3.1_Sobic.002G375400.5
gnl_Ambtr_v1.0_evm_27.model.AmTr_v1.0_scaffold00129.96
gnl_Arath_TAIR10_AT1G72360.2
0.894
gnl_Spipo_v2_Spipo2G0118800
gnl_Ambtr_v1.0_evm_27.model.AmTr_v1.0_scaffold00003.102
0.848
gnl_Pinta_v2.0_PITA_000085849-RA
gnl_Pinta_v2.0_PITA_000070029-RA
gnl_Pinta_v2.0_PITA_000009898-RA
0.976
gnl_Pinta_v2.0_PITA_000008690-RA
0.988
gnl_Pinta_v2.0_PITA_000009900-RA
0.782
gnl_Pinta_v2.0_PITA_000030811-RA
gnl_Pinta_v2.0_PITA_000094240-RA
0.150
gnl_Pinta_v2.0_PITA_000092878-RA
gnl_Pinta_v2.0_PITA_000064383-RA
0.451
gnl_Pinta_v2.0_PITA_000055573-RA
0.995
gnl_Pinta_v2.0_PITA_000058452-RA
gnl_Pinta_v2.0_PITA_000079099-RA
0.822
gnl_Pinta_v2.0_PITA_000030810-RA
gnl_Ambtr_v1.0_evm_27.model.AmTr_v1.0_scaffold00129.86
Maldo_HC_hap2_g49934.t1
0.000
gnl_Sorbi_v3.1_Sobic.007G147300.1
gnl_Phypa_v3.3_Pp3c14_3310V3.1
gnl_Phypa_v3.3_Pp3c14_3280V3.1
0.324
gnl_Phypa_v3.3_Pp3c10_6570V3.1
0.934
gnl_Phypa_v3.3_Pp3c10_6600V3.1
0.966
gnl_Phypa_v3.3_Pp3c1_27630V3.1
gnl_Phypa_v3.3_Pp3c1_27710V3.1
0.953
0.892
gnl_Selmo_v1.0_73134
gnl_Selmo_v1.0_73164
gnl_Phypa_v3.3_Pp3c21_8710V3.1
0.614
gnl_Phypa_v3.3_Pp3c18_10680V3.1
0.835
0.997
gnl_Phypa_v3.3_Pp3c22_10880V3.1
gnl_Phypa_v3.3_Pp3c19_14950V3.1
0.966
gnl_Phypa_v3.3_Pp3c7_1240V3.1
0.928
gnl_Phypa_v3.3_Pp3c11_24520V3.1
gnl_Pinta_v2.0_PITA_000054453-RA
