# Supplementary material for: Transcriptomics of long‐term, low oxygen storage coupled with ethylene signaling interference suggests neofunctionalization of hypoxia response pathways in apple ( Malus domestica )
Source: Plant Direct. 2024 Dec 20;8(12):e70025. doi: 10.1002/pld3.70025 (PMC11660084; doi:10.1002/pld3.70025)
Supplement: Supplementary file 6 — Data S6. Peer Review. [file PLD3-8-e70025-s015.pdf]

Plant Direct
Open Access

Transcriptomics of long-term, low oxygen storage coupled with ethylene signaling interference suggests neofunctionalization of hypoxia response pathways in apple (*Malus domestica*).

Authors

John A. Hadish[1,2,A], Heidi L. Hargarten[3,A], Huiting Zhang[2,3], James P. Mattheis[3], Stephen P. Ficklin[1,2*], Loren A. Honaas[3*]

Decision Letter Round 1:

October 1, 2024

Heidi Hargarten
USDA ARS
Wenatchee, Washington

RE: Transcriptomics of long-term, low oxygen storage coupled with ethylene signaling interference suggests neofunctionalization of hypoxia response pathways in apple (Malus domestica).

Dear Dr. Hargarten:

Thank you for submitting to Plant Direct. I have had a hard time to find an academic editor for this manuscript. In the end, I decided to handle it myself. Please look at the editor's comments below and make revisions as suggested.

When uploading the revised version of this article, please be sure to include the following:

-A word document that contains your response to the reviewers. You should respond to each reviewer comment and note the changes made to the manuscript. If you do not agree with a reviewer's comment and choose not to make a suggested revision, please explain why. Please try to provide as complete an answer as possible to each reviewer's criticisms.
-A tracked changes document with each change highlighted
- A clean version of the latest version of the manuscript

To upload your revision, please click the link below.

https://plantdirect.msubmit.net/cgi-bin/main.plex?el=A1Lr2jy4A6olZ3I6A9ftdn8FUH9EA0K12LZzUxArjgZ

In order to provide as timely a service as possible, we ask that your revision is resubmitted within three months after receipt of this request. If an extension is needed, please send a request, along with a brief explanation, to the editorial office at plantdirect@wiley.com .

Please note that, in addition to publishing reviewer comments, the author's responses to review comments will also be published alongside the final version of the paper. If you would not like the author's responses to be published, please contact the editorial office at plantdirect@wiley.com .

Thank you very much for giving us an opportunity to review your work. I look forward to receiving the next version.

Sincerely,

Feng Chen

Supervising Editor, Plant Direct

--- Editor comments:
This work is generally well conducted and reveals important new insights into the mechanisms for long-term storage of apples under low oxygen and ethylene signaling interference conditions. I have two comments for its improvement. First, the introduction is too long. I suggestion to shorten it and make it more relevant to the work conducted. Second, in Results, present the data for overall pattens of global gene expression before intruding specific genes families.

Decision Letter Round 2:

October 29, 2024
Heidi Hargarten

Plant Direct
Open Access

USDA ARS
Wenatchee, Washington

MSID: 2024-01597-TWR1
MS TITLE: Transcriptomics of long-term, low oxygen storage coupled with ethylene signaling interference suggests neofunctionalization of hypoxia response pathways in apple (Malus domestica).

Dear Dr. Heidi Hargarten:

I am pleased to inform you that your manuscript "Transcriptomics of long-term, low oxygen storage coupled with ethylene signaling interference suggests neofunctionalization of hypoxia response pathways in apple (Malus domestica)." has been accepted for publication in Plant Direct.

Your article will appear online in the next available issue of Plant Direct. To ensure your article gets published as quickly as possible, please pay attention to the steps detailed below. We have found that most of the delays happen at this stage, especially at the payment stage, so please respond as quickly as possible when prompted.

License Agreement: Once your article has been accepted it will move to Production and undergo admin and file checking - you may receive an email with any queries we have at this stage. When all required items are received by the publisher and queries resolved, the corresponding author will receive an email from Wiley's Author Services system which will ask them to log on at https://authorservices.wiley.com/bauthor and will present them with the appropriate license for completion. Your article cannot be published until both the signed license agreement and payment of the article fee have been received.

Payment of the Open Access Article Publication Fee: All articles published in Plant Direct are fully open access: immediately and freely available to read, download and share. Plant Direct charges a publication fee to cover the publication costs. The corresponding author for this manuscript should have already received a quote from the payments team (cs-openaccess@wiley.com) with the estimated article publication fee; please email cs-openaccess@wiley.com if this has not been received. The corresponding author should log on to the Wiley Author Services site, where the publication fee can be paid by credit card or an invoice. Pro Forma can also be requested. Payment of the publication charge must be received before the article will be published online.

Proofs: You will have the opportunity to look over your paper once more when you receive the author proofs for your article. The proofs will be with you in approximately two weeks. Please note that, in addition to publishing reviewer comments, the author's responses to review comments will also be published alongside the final version of the paper. If you would not like the author's responses to be published, please contact the editorial office at plantdirect@wiley.com .

Promotion of your article: You can help your research get the attention it deserves! Check out Wiley's free Promotion Guide for best-practice recommendations for promoting your work at www.wileyauthors.com/eeo/guide. And learn more about Wiley Editing Services which offers professional video, design, and writing services to create shareable video abstracts, infographics, conference posters, lay summaries, and research news stories for your research at www.wileyauthors.com/eeo/promotion.

Thank you again for your contribution to Plant Direct. If you have any questions, feel free to contact the editorial office at plantdirect@wiley.com .

Sincerely,

Feng Chen

Editor, Plant Direct

Response to Reviewer:

We thank the editor for their comments and the opportunity to improve our manuscript. We have provided responses to their comments below, response text colored in **blue.**

First, the introduction is too long. I suggestion to shorten it and make it more relevant to the work conducted.

The introduction has been shortened by removing sections not directly related to the work we conducted. The introduction now spans lines 41-111, the original submission spanned 41-147.

Second, in Results, present the data for overall pattens of global gene expression before intruding specific genes families.

The Results section has been restructured so that the presentation of global gene expression results now comes before the presentation of specific gene families. Global gene expression presentation is now sections 4.1 and 4.2, while gene family specific presentation is now sections 4.3 and 4.4. The numbering of figures and supplemental figures within those sections has been reordered accordingly to reflect this restructuring.

See below for original comments (provided in email):

RE: Transcriptomics of long-term, low oxygen storage coupled with ethylene signaling interference suggests neofunctionalization of hypoxia response pathways in apple (Malus domestica).

Dear Dr. Hargarten:

Thank you for submitting to Plant Direct. I have had a hard time to find an academic editor for this manuscript. In the end, I decided to handle it myself. Please look at the editor's comments below and make revisions as suggested.

When uploading the revised version of this article, please be sure to include the following:

-A word document that contains your response to the reviewers. You should respond to each reviewer comment and note the changes made to the manuscript. If you do not agree with a reviewer's comment and choose not to make a suggested revision, please explain why. Please try to provide as complete an answer as possible to each reviewer's criticisms.
-A tracked changes document with each change highlighted
- A clean version of the latest version of the manuscript

To upload your revision, please click the link below.
https://plantdirect.msubmit.net/cgi-bin/main.plex?el=A1Lr2jy4A6olZ3I6A9ftdn8FUH9EA0K12LZzUxArjgZ

In order to provide as timely a service as possible, we ask that your revision is resubmitted

within three months after receipt of this request. If an extension is needed, please send a request, along with a brief explanation, to the editorial office at plantdirect@wiley.com .

Please note that, in addition to publishing reviewer comments, the author's responses to review comments will also be published alongside the final version of the paper. If you would not like the author's responses to be published, please contact the editorial office at plantdirect@wiley.com .

Thank you very much for giving us an opportunity to review your work. I look forward to receiving the next version.

Sincerely,

Feng Chen

Supervising Editor, Plant Direct

-- Editor comments:
This work is generally well conducted and reveals important new insights into the mechanisms for long-term storage of apples under low oxygen and ethylene signaling interference conditions. I have two comments for its improvement. First, the introduction is too long. I suggestion to shorten it and make it more relevant to the work conducted. Second, in Results, present the data for overall pattens of global gene expression before intruding specific genes families.
